# Microbial dynamics in gastric cancer: insights from full-length 16S rRNA nanopore sequencing in the MAGIC cohort

**DOI:** 10.3389/fmicb.2026.1841026

**Published:** 2026-06-09

**Authors:** Daniela Zapata-Contreras, Jacqueline Aldridge, Jorge González-Puelma, Marcelo Navarrete, Claudio Urrea, Fernando Orellana, María José Iriarte, Carlos Delgado, Marcela Puente, Lissette Leiva, Luis Godoy, Alejandro Altamirano, Stanko Karelovic, Yolanda Espinosa-Parrilla

**Affiliations:** 1 School of Medicine, Magallanes University, Punta Arenas, Chile; 2 Evolutionary and Medical Genomics in Magallanes (GEMMa), Center for Education, Healthcare and Investigation (CADI -UMAG), Magallanes University, Punta Arenas, Chile; 3 Chilean Hereditary Cancer Group (GCCH), Punta Arenas, Chile; 4 Centro Austral de Tecnología Genómica (cATG), Center for Education, Healthcare and Investigation (CADI -UMAG), Magallanes University, Punta Arenas, Chile; 5 Department of Computer Engineering, Magallanes University, Punta Arenas, Chile; 6 Hospital Clínico Magallanes (HCM), Punta Arenas, Chile; 7 Interuniversitary Center for Healthy Aging (CIES), Punta Arenas, Chile

**Keywords:** 16S rRNA sequencing, carcinogenesis, dysbiosis, gastric cancer, *Helicobacter pylori*, microbiome

## Abstract

Gastric dysbiosis, characterized by shifts in the microbial composition, has been increasingly associated with the development of gastric cancer, the fifth leading cause of cancer-related deaths worldwide and the second in Chile, yet its characterization through disease stages remains limited and its study in Latin American populations almost non-existent. While *Helicobacter pylori* is a well-established risk factor, recent evidence supports the involvement of non-*Helicobacter pylori* bacteria associated with disease progression, emphasizing the need to characterize the gastric microbiome in diverse populations and through cancer stages. In this study, 162 endoscopic biopsy tissues and gastrectomy tissues from 83 Chilean individuals enrolled in the Magellanic gastric cohort MAGIC and the BTUCH cohort were analyzed using high throughput full-length 16S rRNA sequencing based on Nanopore technology. Diversity analysis demonstrated significant differences among disease progression and histological subtypes. Spearman correlation identified 34 genera significantly associated with gastric cancer progression, including enrichment of *Lactobacillus* and *Limosilactobacillus*. *Helicobacter* stratification analysis revealed lower diversity and distinct community structure at early stages of disease. Declining *Helicobacter* abundance was associated with shifts toward degradation and biosynthetic/energy metabolism pathways suggesting potential metabolic adaptation in carcinogenesis. These findings reveal stage-specific restructuring of the gastric microbiota along disease progression and identify non-*Helicobacter* taxa as part of microbial signatures associated with different stages of gastric carcinogenesis.

## 1 Introduction

Microbiome dysbiosis has been implicated in multiple pathological conditions, including gastric cancer (GC). GC continues to be one of the leading cancers worldwide, being the 5th cancer in terms of global incidence and mortality according to GLOBOCAN 2022 (Bray et al., 2024). In Chile, GC represented the fourth place in incidence among cancer diagnoses in 2022, with 4,955 new cases, but ranked second in cancer-related mortality. GC is a complex disease involving many inherited and environmental factors including changes in the microbiota composition. The human microbiota is critical for maintenance of human health and plays an integral role in energy metabolism, absorption of nutrients, and defense against invading pathogens (Zhang et al., 2025). However, this microbiota exists within a delicate balance that, if altered, becomes dysbiotic and contributes to aberrant proinflammatory immune responses and initiation of disease processes, including cancer (Schwabe and Jobin, 2013).

From the histopathological perspective, GC can be classified in different subtypes, one of the most known is the Lauren’s classification, which distinguishes the diffuse, the intestinal, and the mixed subtypes (Lauren, 1965). These subtypes present different characteristics, including clinical features, genetics, morphology, epidemiology and expansion properties where the diffuse subtype displays poorly cohesive single cells without gland formation and the intestinal subtype encompasses tubular and glandular elements, with multiple degrees of differentiation (Machlowska et al., 2020). A model of gastric carcinogenesis is related to the intestinal type, where GC is preceded by a prolonged precancerous process described as Correa cascade (Correa, 1992). This model explains the following consecutive steps: normal gastric mucosa, superficial gastritis or non-atrophic gastritis (SG), multifocal atrophic gastritis (AG) without intestinal metaplasia, intestinal metaplasia (IM), dysplasia (DY; low and high grade) and invasive adenocarcinoma (Correa and Piazuelo, 2012). SG is characterized by an acute inflammation, and it may either remain as non-atrophic or progress in severity, presenting damage in the gastric glands, which may eventually disappear, leading to AG. IM represents a phenotypic change from the normal epithelial cell of gastric mucosa to an intestinal phenotype and is considered an advanced stage of atrophy because the metaplastic glands replace the original glands. DY is characterized by a neoplastic phenotype, where the architecture of the dysplastic tissue no longer preserves well-organized glands. Next stage in the cascade requires the penetration of neoplastic cells into the surrounding stroma, known as invasive carcinoma (Correa and Piazuelo, 2012). All steps of the precancerous cascade have been associated with changes in the microbiota, precisely the establishment of *Helicobacter pylori* (*H. pylori*) infection is accepted as a major risk factor for GC (Robin Warren and Marshall, 1983). The correlation between microbiota and cancer has previously been investigated (Bray et al., 2024), and it has been related to the pathogenesis of gastrointestinal carcinomas (Yamamura et al., 2016; Sears and Garrett, 2014; Wang et al., 2014). *H. pylori* infection, first identified by Marshall and Warren, has been recognized as one of the main etiological factors associated with GC (Robin Warren and Marshall, 1983) and it is critically involved in the early stages of gastric carcinogenesis by promoting sustained inflammation and gradual disruption of the structure and function of the gastric epithelium (Correa, 1992). *H. pylori* initially survives the acidic gastric environment by locally increasing pH through urease-mediated ammonia production (Ohno and Satoh-Takayama, 2020). Prolonged inflammation leads to the development of chronic gastritis, which is characterized by reduced gastric acidity, and the resulting hypochlorhydric environment promotes the growth of other bacterial species that are able to generate reactive oxygen and nitrogen compounds, which may further modulate inflammation (Sheh and Fox, 2013; Engstrand and Lindberg, 2013). In addition, it has been reported *H. pylori* as a major modulator of the gastric microbiome (Spiegelhauer et al., 2020). Despite the fact that the most frequent cause of gastritis is *H. pylori* infection (Correa and Piazuelo, 2012), not all infected individuals develop GC indicating that other contributing factors are necessary to fully account for the development of the disease (Noto and RM, 2017).

For many years, technical limitations limited the study of the human gastrointestinal microbiota. The development of culture-independent methods, particularly 16S rRNA gene sequencing, has greatly improved microbial characterization (Wang et al., 2025). Illumina technology is the most common sequencing platform for 16S rRNA gene metabarcoding, but advances in high throughput sequencing technologies, such as Pacific Biosciences (PacBio) and Oxford Nanopore Technologies (ONT) allow for long-read 16S rRNA sequencing (Shin et al., 2016; Heikema et al., 2020; Pollock et al., 2018). Unlike short-read approaches that target selected hypervariable regions (e.g., V3-V4), full-length 16S rRNA sequencing covers V1-V9 regions, improving taxonomic resolution and enabling more accurate genus-level and potential species-level discrimination (Matsuo et al., 2021).

To date, several studies have highlighted a role for the gastric microbiota in GC, demonstrating that the microbiome composition in GC is significantly different to stages of carcinogenesis (Kupcinskas and Hold, 2018). Bacterial genera such as *Streptococcus* have been repeatedly observed in premalignant lesions and in GC, while *Fusobacterium* abundance is reported to increase in GC compared with precancerous stages (Zeng et al., 2024; Jeong et al., 2024; Lehr et al., 2023). *Fusobacterium* has been reported to stimulate gastric epithelial cells, activating proinflammatory and pro-oncogenic pathways, and to induce DNA double-strand breaks via metabolite secretion (Niikura et al., 2023). Supporting these observations, a meta-analysis including 2,198 individuals identified *Fusobacterium*, *Leptotrichia*, and *Streptococcus anginosus* as significantly enriched in GC patients compared with patients with gastritis (Li et al., 2023). Also, a marked reduction in *α*-diversity and a decreased abundance of *H. pylori*, alongside enrichment of oral and intestinal commensal species, have been described in GC (Kupcinskas and Hold, 2018; Hsieh et al., 2018). Therefore, Coker et al., (2018) identified coordinated microbial changes during GC progression, highlighting several oral-associated taxa, such as *Peptostreptococcus stomatis* and *Streptococcus anginosus*, as central nodes in GC-related interaction networks. Notably, microbial interactions were stronger in *H. pylori*-negative precancerous samples, underscoring the influence of *H. pylori* on gastric microbial dynamics (Coker et al., 2018). Other studies have consistently reported higher proportions of *Lactobacillus* and *Lactococcus* in GC patients compared with controls (Aviles-Jimenez et al., 2014; Eun et al., 2014; Castaño-Rodríguez et al., 2017), which have been proposed to produce lactic acid, which can serve as an energy source for tumor growth and angiogenesis (Sonveaux et al., 2012). Additional genera enriched in GC include *Prevotella, Selenomonas, Stomatobaculum* and *Lachnospiraceae* (Lehr et al., 2023; Booth et al., 2025). Despite these findings, the results from different studies are highly heterogeneous, indicating the large variations of gastric microbiota among and through distinct population (Zeng et al., 2024). Most studies have been mainly focused in European and Asian cohorts, highlighting a critical lack of data from underrepresented populations such as those in Latin America (Coker et al., 2018; Abdill et al., 2025). For this reason, studies incorporating diverse populations, disease stages, and histological subtypes are essential to better define population-specific microbial signatures and clarify their role in gastric carcinogenesis.

The aim of this study was to characterize the gastric microbial community through GC and in previous stages of the disease to explore its potential associations with the development and progression of GC. To address existing knowledge gaps, this work includes representative tissues from different histological subtypes and stages of disease progression in a Chilean population, which remains largely underrepresented in microbiome research.

## 2 Materials and methods

### 2.1 Sample collection

A prospective cohort of 551 individuals (MAGIC Cohort) was recruited from November 2018 to November 2022 (with a pause from March 2020 to April 2021 due to COVID-19 pandemics) at the gastroenterology unit of the “Hospital Clínico Magallanes” (HCM), in Punta Arenas, the capital of the Magallanes and Chilean Antarctic region, the southernmost region of Chile. The main inclusion criteria were referral for upper endoscopy for suspected gastric disease according to standard criteria (i.e., symptoms such as abdominal pain, difficulty swallowing, and prolonged nausea and vomiting). Cases with histopathological features consistent with autoimmune gastritis as well as cases presenting recent exposure to antibiotics were excluded from the study. Five samples from three different regions of the stomach (two from the body, two from the pyloric antrum and one from the angular notch) were examined according to the Sydney protocol and recommendations from the Chilean association of digestive endoscopy (ACHED). The diagnosis of GC was performed according to the histological examination of these endoscopic samples in the department of pathology of the hospital. The subtype of GC was assessed according to standard criteria. Endoscopic biopsy tissues from the antrum and body of stomach were collected during endoscopy and frozen immediately at −80° C. Gastrectomy tissues were obtained from sites of tumoral and adjacent to tumor mucosa and frozen immediately at −80 °C. A maximum of two biopsy tissues and two gastrectomy tissues were collected per individual. Written informed consent was obtained from all the participants of the study, which was approved by the Ethics Committee of the University of Magallanes. From MAGIC Cohort, 52 individuals with collected tissues were selected. This comprised 78 biopsy tissues obtained from 40 individuals and 24 gastrectomy tissues from 12 individuals. Additional 60 gastrectomy tissues from 31 individuals were obtained from the Biobank of Tissue and Fluids of the University of Chile (BTUCH). The study data was collected and managed using REDCap (Research Electronic Data Capture).

### 2.2 Genomic DNA extraction

DNA extractions from tissues were performed using QIAamp PowerFecal Pro DNA Kit (QIAGEN) following manufacturer’s instructions, with an additional 30 min incubation at 60 °C with Proteinase K (20 mg/mL) during the lysis step. Quality assessment of the DNA was conducted using the Nanodrop One spectrophotometer (Thermo Fisher Scientific).

### 2.3 16S rRNA gene amplification

Amplification of a 16S rRNA gene fragment of ~1,500 bp was performed using 20 ng of DNA per sample. Universal primers 8F, 5′ – AGA GTT TGA TCC TGG CTC AG – 3′ and 1492R, 5′ - GGT TAC CTT GTT ACG ACT T – 3′ were used for PCR amplification with Phusion HF DNA Polymerase (Thermo Fisher Scientific). Thermal cycling was carried out under the following cycling conditions: an initial denaturation step at 98 °C for 1 min, followed by 35 cycles of denaturation at 98 °C for 10 s, annealing at 59 °C for 30 s, and extension at 72 °C for 1 min, with a final extension at 72 °C for 10 min. Amplicon size and quality assessment was conducted using the TapeStation 4,200 system (Agilent) with the D5000 ScreenTape assay. Negative PCR controls were included during 16S rRNA amplification to monitor potential reagent or amplification-related contamination.

### 2.4 Oxford nanopore sequencing

Library preparation for full-length 16S rRNA gene amplicon sequencing was performed using the Ligation Sequencing Amplicons – Native Barcoding 96 v14 kit (SQK-NBD114.96, ONT), enabling multiplexing of up to 96 samples per run, according to the manufacturer’s instructions, including DNA repair, end-prep, native barcoding, adapter ligation and clean-up steps. Final library was quantified using the Qubit dsDNA BR Assay Kit on a Qubit fluorometer (Thermo Fisher Scientific). Sequencing was performed in a FLO-PRO114M flow cell, using a PromethION 2 Solo device (ONT). Sequencing experiments were performed in flow cells with >6,000 active pores and the run was carried out for 26 h. The sequencing coverage and quality statistics for each sample are detailed in the Supplementary Table 1. Information regarding data availability can be found in the Data Availability section.

### 2.5 Sequencing analysis and 16S rRNA data analysis

Raw full-length 16S rRNA gene sequencing data were processed using different bioinformatics tools and in-house scripts. Basecalling was performed using Dorado (Oxford Nanopore Technologies, 2026) with the super accuracy (SUP) model, and demultiplexing was carried out according to the barcoding kits used during library preparation. Reads with a quality score below 20 were discarded, and only those ranging from 1,000 to 2,000 base pairs in length were retained using Nanoq (Steinig and Coin, 2022). Taxonomic classification was performed by direct read alignment with MMSeqs2 (Steinegger and Söding, 2017) against the GenBank 16S rRNA reference database (Bethesda (MD), 1988), because of its broad taxonomic coverage and compatibility with full-length alignment-based classification. Only alignments with a percentage identity greater than 95% and an alignment length >1,000 base pairs were considered for downstream analyses. After taxonomic assignment, a total of 138 tissues were included in the study with a technical criterion of approximately 5,000 assigned reads to phylum per sample and were retained for downstream analyses (Supplementary Figure 1 and Supplementary Table 1). The final dataset included 46 individuals from MAGIC cohort and 29 individuals from the BTUCH cohort. To ensure comparison between cohorts, all analyses were performed using a consistent preprocessing, taxonomic assignment, and normalization framework. To reduce biases introduced by the heterogeneity in the sequencing depth, read counts from retained tissues were transformed into relative abundances prior to diversity and compositional analyses. Given the exploratory nature of the study and the limited sample availability in some diagnostic groups, analyses were performed at the tissue-sample level.

Taxa annotated as “(mixed species)” indicate ambiguous taxonomic assignments that could not be confidently resolved to a single genus. According to NCBI Taxonomy conventions, taxa with bracketed genus names (e.g., *“[Eubacterium] nodatum*”) denote provisional genus-level assignments whose phylogenetic placement is unresolved and pending formal reclassification (NLM Customer Support Center, 2026).

### 2.6 Statistical analyses

Microbial diversity was assessed using both *α*- and *β*-diversity metrics. *α*-diversity indices, including observed Richness, Shannon, and Simpson indices, were calculated to evaluate within-sample diversity, and pairwise Wilcoxon rank-sum tests were applied to assess differences among groups. *β*-diversity was used to evaluate variation in microbial community composition among tissues and was calculated based on Bray–Curtis dissimilarity (Bray and Curtis, 1957). Community-level differences were visualized using non-metric multidimensional scaling (NMDS) (Oksanen et al., 2025), and statistical significance of compositional differences among groups was assessed using PERMANOVA (Anderson, 2001). Differential abundance analysis at the genus level was performed using DESeq2 in R Studio (Love et al., 2014). Fold-Change (FC) results were visualized using volcano plots to highlight significantly enriched or depleted taxa among comparison groups. Spearman rank correlation analysis was conducted to evaluate associations between genus-level relative abundances and either disease progression stages or *Helicobacter* relative abundance. Correlation coefficients (*ρ*) were calculated, and multiple-testing correction was applied using the false discovery rate (FDR) to identify statistically significant associations. Predicted functional profiles were generated using PICRUSt2 (Douglas et al., 2020) and analyzed at the pathway level based on the MetaCyc database. Differential pathway enrichment was assessed using LEfSe (Segata et al., 2011), combining non-parametric statistical testing with linear discriminant analysis to identify pathways discriminating between groups.

### 2.7 Bioethics

The study was conducted under ethical and scientific standards and was approved by the relevant institutional ethical review boards. Written informed consent was obtained from all the participants of the study, which was approved by the Ethics Committee of the University of Magallanes.

## 3 Results

### 3.1 Sequencing performance and quality metrics

Full-length 16S rRNA nanopore sequencing generated high-depth datasets throughout endoscopic biopsy tissues and gastrectomy tissues. Sequencing yielded a mean of 643,102 reads per sample (median 538,512; range 45,569–2,491,797). The mean read length was 1,157 bp (median 1,213 bp), with a length-weighted median (N50) of 1,534 bp, consistent with successful recovery of near full-length 16S amplicons. The mean quality score was 17.7. Detailed per-sample sequencing coverage and quality metrics are summarized in Supplementary Table 1.

### 3.2 Gastric tissues collection

**Figure 1:**
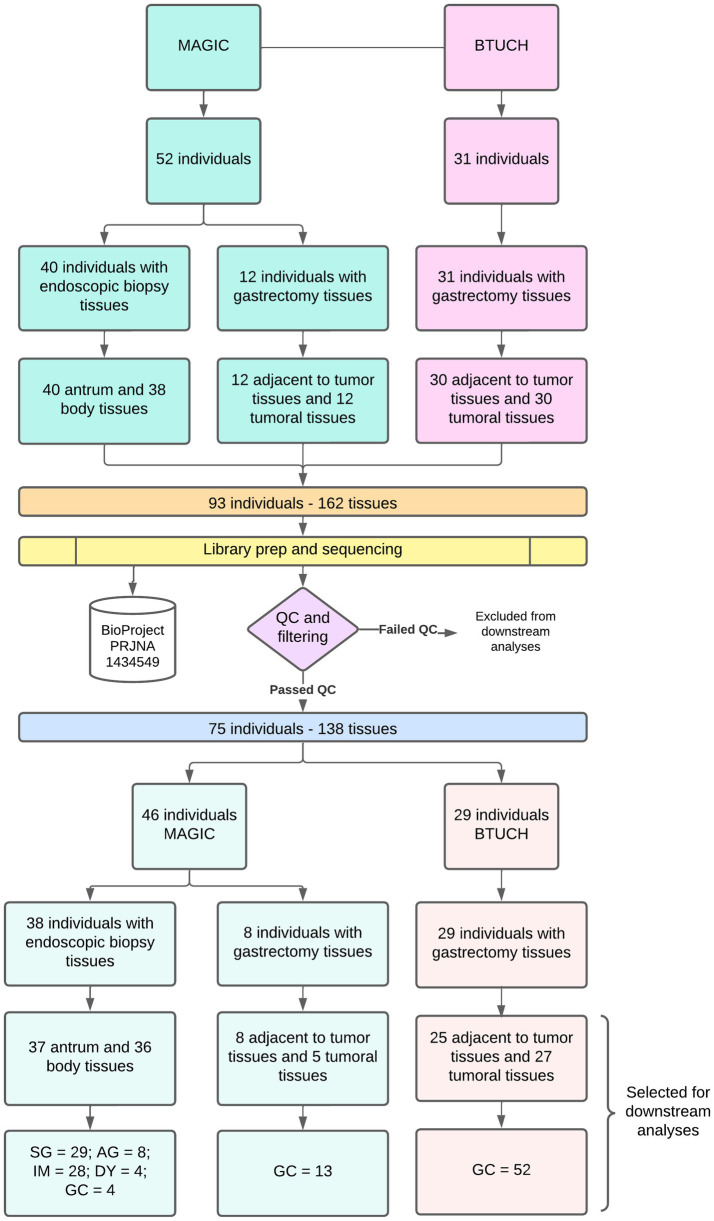
Cohort composition, sample processing, and analytical workflow of the study. Flowchart summarizing cohort composition, tissue distribution, sequencing workflow, quality-control filtering, and downstream analyses included in the study. Samples from the MAGIC and BTUCH cohorts comprised endoscopic biopsy tissues and gastrectomy adjacent and tumoral tissues representing different stages along the Correa cascade. After library preparation and sequencing, samples underwent quality-control filtering prior to downstream microbiome analyses. SG, Superficial gastritis; AG, Atrophic gastritis; IM, Intestinal Metaplasia, DY, Dysplasia; GC, Gastric Cancer.

The study considered a total of 83 individuals, distributed among two independent cohorts: MAGIC (*n* = 52) and BTUCH (*n* = 31). After full-length 16S rRNA sequencing and taxonomic assignment, according to the technical criteria detailed in Material and Methods section, 73 endoscopic biopsy tissues (antrum and body) from 38 individuals and 13 gastrectomy tissues (adjacent to tumor and tumoral) from 8 individuals were retained for downstream analyses in the MAGIC cohort. The biopsy tissues represented different precancerous and cancerous progression stages, including SG (*n* = 29), AG (*n* = 8), IM (*n* = 28), DY (*n* = 4), and GC (*n* = 4). The 13 gastrectomy tissues from MAGIC corresponded exclusively to advanced GC (AGC) and were further classified into histological subtypes as diffuse (*n* = 3), intestinal (*n* = 6), and mixed (*n* = 4). In the BTUCH cohort, 52 gastrectomy tissues (adjacent and tumoral) from 29 individuals were analyzed. Of these, 10 gastrectomy tissues were classified as early GC (EGC), all with diffuse subtype, and 42 gastrectomy tissues were classified as AGC, comprising diffuse (*n* = 24), intestinal (*n* = 16), and mixed (*n* = 2) subtypes. A detailed overview of sample selection and classification is provided in the study flowchart (Figure 1), while cohort composition and characteristics are summarized in Supplementary Tables 2 and 3.

### 3.3 Microbiome along GC progression in biopsy tissues

**Figure 2:**
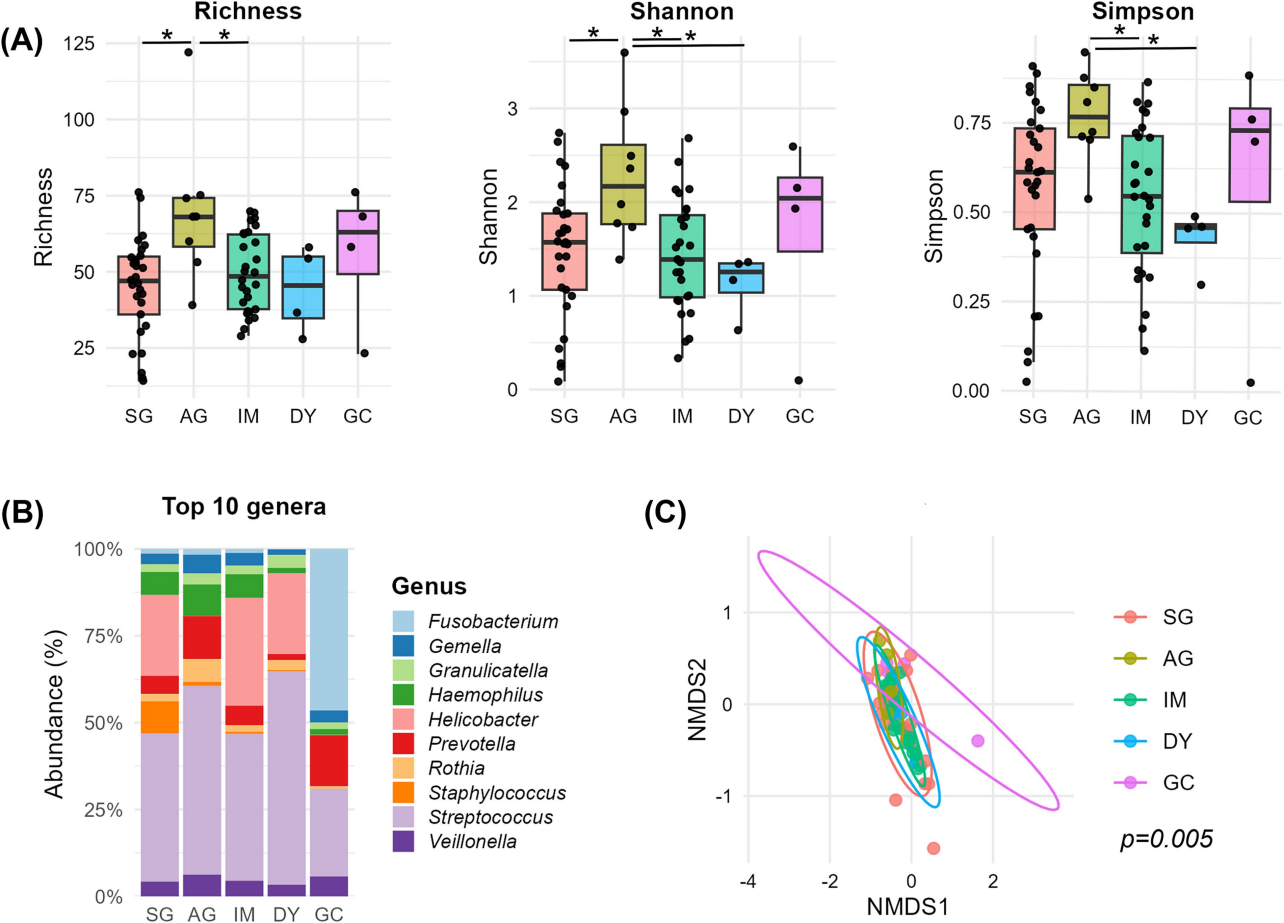
*α*- and *β*-diversity metrics in biopsy samples from the MAGIC cohort grouped by progression stages. **(A)** α-diversity was assessed using Richness, Shannon and Simpson indices; statistical significance was determined using Pairwise Wilcoxon test (*p* < 0.05); **(B)** Top 10 most abundant genera in biopsies grouped by progression stage; **(C)** Non-metric Multidimensional Scaling (NMDS) plot illustrating the clustering patterns of the microbial communities in biopsies grouped by progression stage. Statistical significance was determined using PERMANOVA (*p* < 0.05). SG, Superficial gastritis; AG, Atrophic gastritis; IM, Intestinal Metaplasia; DY, Dysplasia; GC, Gastric Cancer.

To explore the role of gastric microbiome along the Correa cascade (SG, AG, IM and DY), the bacterial community composition and diversity was analyzed in 73 biopsy tissues from the MAGIC cohort. A higher microbiome diversity in the AG group compared with any of the other diagnostic groups was found, being the difference statistically significant when AG was compared with the SG, IM and DY groups (Figure 2A). The GC group did not show statistically significant differences relative to the remaining groups (Figure 2A).

Next, the microbiome composition was characterized by identifying the topmost abundant bacterial genera. The most common genera present in all biopsy tissues were *Streptococcus, Gemella, Granulicatella, Haemophilus, Helicobacter, Prevotella, Rothia, Staphylococcus,* and *Veillonella*, with an important presence of *Fusobacterium* and a decrease of *Helicobacter* in the GC group (Figure 2B). Further, NMDS ordination based on Bray–Curtis dissimilarities were used to visualize differences in genus-level community composition; PERMANOVA analysis indicated significant differences among diagnostic groups (*p* = 0.005), supporting compositional shifts in disease progression (Figure 2C). No statistically significant differences were found between the antrum and body tissues (Supplementary Figure 2).

**Figure 3:**
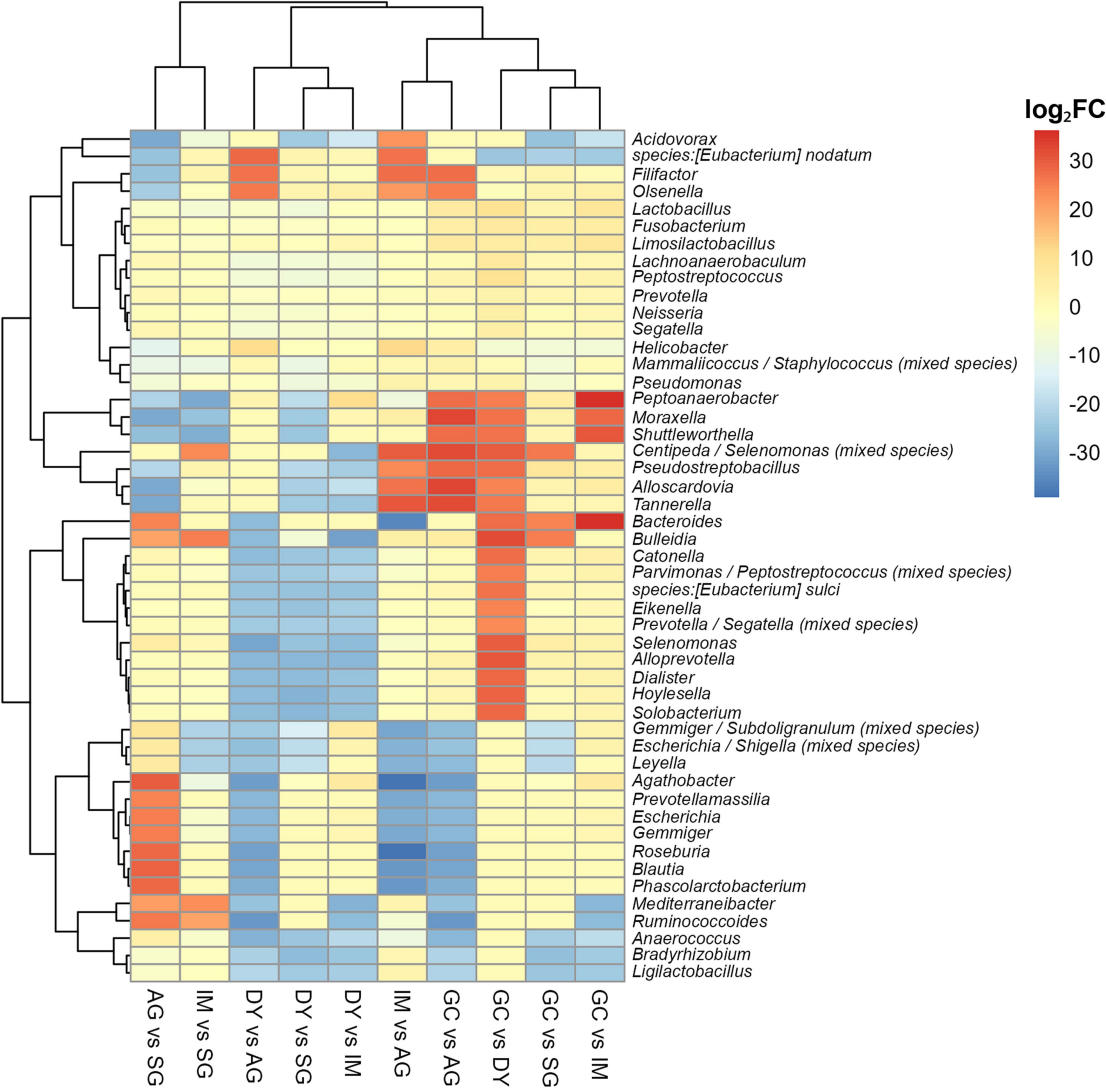
Heatmap of log₂FC for differentially abundant genera in pairwise comparisons in biopsy samples from the MAGIC cohort. Genus-level count data from biopsy samples were analyzed using DESeq2. Only genera with at least one statistically significant difference (adj. *p*-value <0.05) in any comparison were included in the heatmap. Rows represent genera, columns represent pairwise comparisons between progression groups, and colors indicate log₂FC values. Positive log₂FC values indicate enrichment in the first group.

To further characterize microbial shifts along gastric disease progression, a differential abundance analysis was performed in biopsy tissues using DESeq2 revealing statistically significant differences in the bacterial composition among progression stages (Supplementary Table 4, 5). A heatmap of significant changes (adj. *p*-value <0.05) highlights microbial signatures associated with GC progression in the sequential stages (Figure 3). According to this, as disease progressed from SG to AG, 13 genera were enriched, including *Blautia* and *Roseburia,* and 13 genera were decreased, including *Helicobacter*, *Tannerella* and *Alloscardovi.* From AG to IM, 11 genera were enriched including *Tannerella* and *Centipeda/Selenomonas,* and 12 genera were decreased, including *Roseburia* and *Agathobacter.* Further along the cascade, from IM to DY, 21 genera were decreased, including *Bulleidia* and *Mediterraneibacter.* Finally, from DY to GC, 22 genera were markedly enriched, including *Bulleidia* and *Alloprevotella* and only *species*: *[Eubacterium] nodatum*, was decreased.

**Figure 4:**
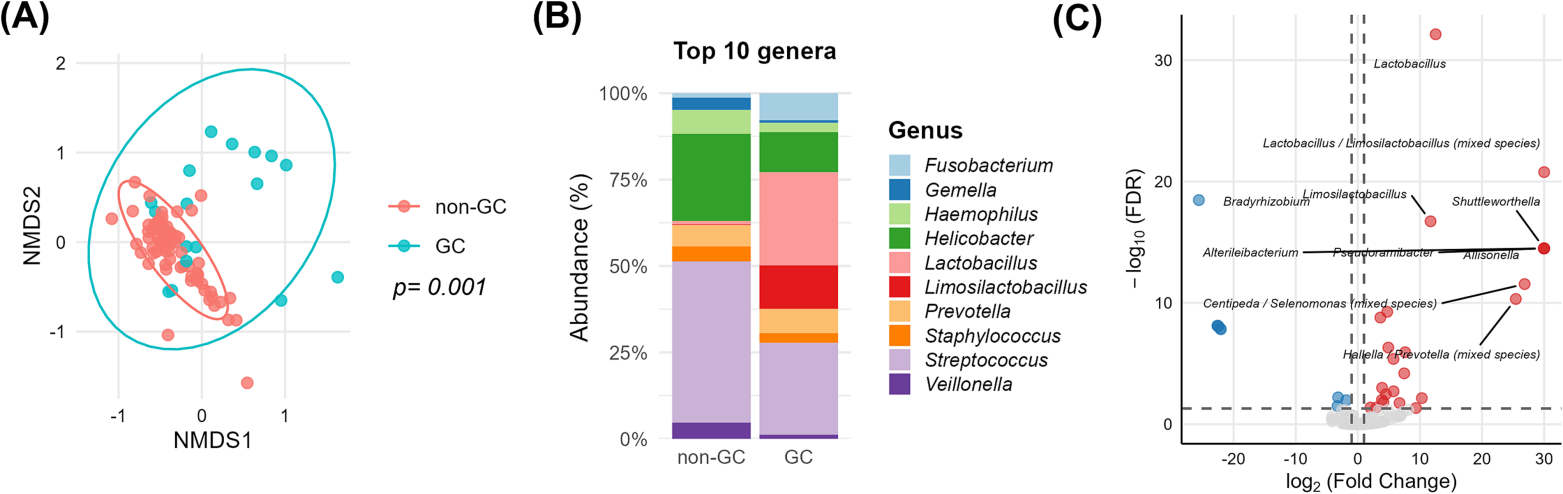
Comparative analysis of gastric microbiota composition and differential abundance between non-GC and GC tissues in the MAGIC cohort. **(A)** Non-metric Multidimensional Scaling (NMDS) plot illustrating the clustering patterns of the microbial communities grouped by GC and non-GC; statistical significance was determined using PERMANOVA (*p* < 0.05); **(B)** Volcano plot between non-GC and GC from the MAGIC cohort; the x-axis represents the log₂FC, while the y-axis shows the −log₁₀ of the adj. *p*-value; genera with significant increases in abundance in GC (log₂FC > 1, FDR < 0.05) are shown in red, and those significantly decreased in GC (log₂FC < −1, FDR < 0.05) in blue; **(C)** Top 10 most abundant genera in tissues grouped by non-GC and GC individuals.

Next, to explore microbial differences between GC diagnosis and non-GC, comprising both MAGIC biopsy tissues and gastrectomy tissues, the differential abundance of genera between non-GC group tissues (SG, AG, IM and DY) and the GC group tissues was analyzed. No statistically significant differences in *α*-diversity were observed among groups (Supplementary Figure 3), but *β*-diversity analysis demonstrated a significantly different bacterial composition between tissues from the GC and non-GC groups (PERMANOVA, *p* = 0.001; Figures 4A,B), which highlights the differential abundance of bacterial genera between both groups as assessed by DESeq2, revealing microbial compositional differences associated with GC, with strongly diminished genera in GC such as *Bradyrhizobium*, *Brevundimonas* and *Acidovorax*. In contrast, genera such as *Lactobacillus* and *Limosilactobacillus*, exhibited a marked increase in GC (Figure 4C). Among them, *Lactobacillus* stands out as one of the taxa most strongly associated with GC tissues (FC = 12.53; *p* = 7.414 × E-33) (Supplementary Table 6).

### 3.4 Microbiome among GC histological subtypes in gastrectomy tissues

Next, the bacterial composition in pairs of gastrectomy-derived tumoral and adjacent to tumor tissues (intestinal, diffuse and mixed GC subtypes) from the MAGIC cohort was evaluated. Overall, there were no statistically significant differences in *α*-diversity between adjacent and tumoral tissues and the NMDS analysis did not reveal a clear clustering between tumoral or adjacent tissues (Supplementary Figure 4). However, specific changes in bacterial composition could be noted, as the apparition of *Carnobacterium* and *Exiguobacterium* genera in the tumoral tissues (Supplementary Figure 4). Regarding the histological GC subtypes, no statistically significant differences in *α*-diversity nor in the NMDS analysis were observed among these groups (Supplementary Figure 5).

**Figure 5:**
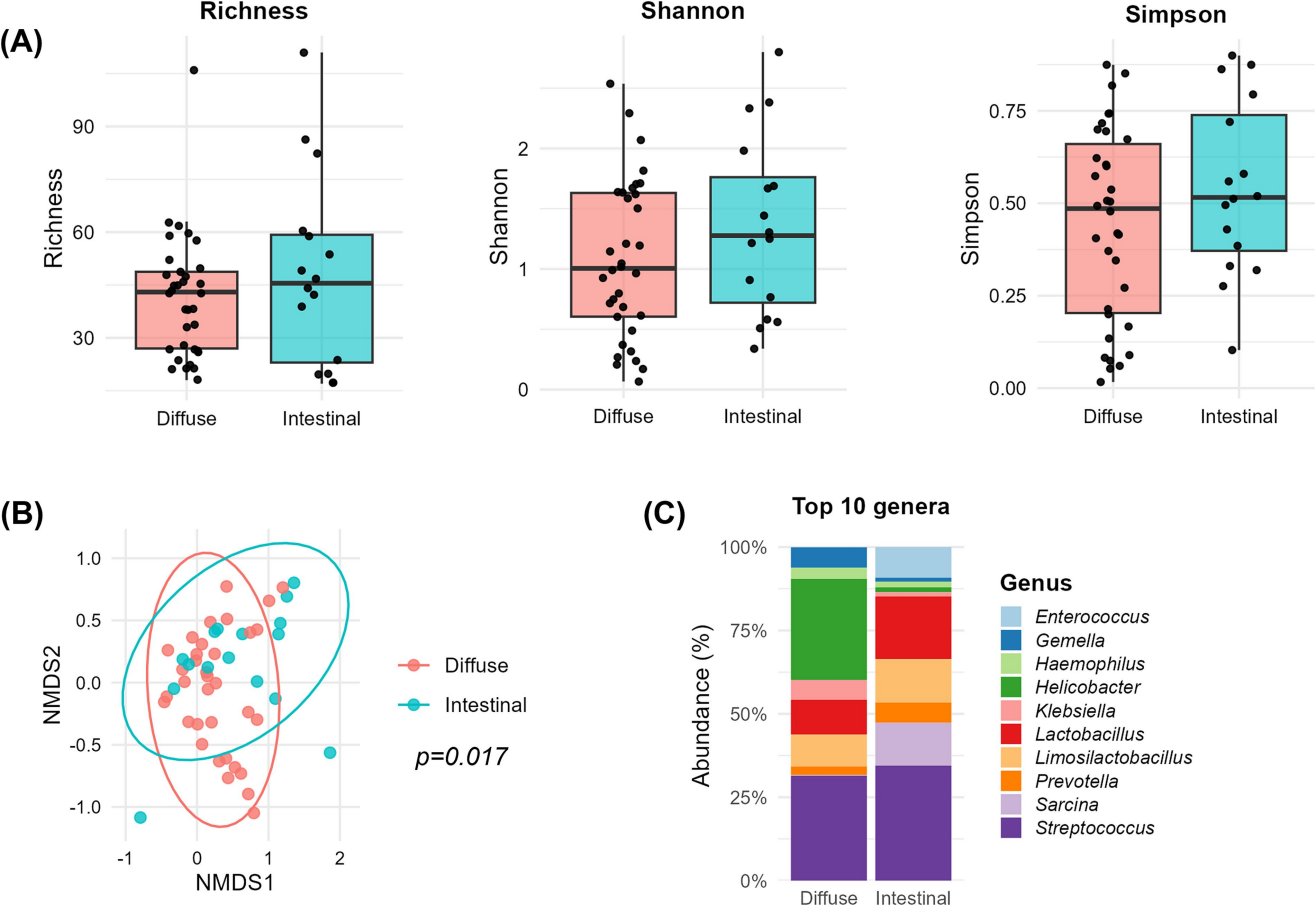
α- and β-diversity metrics in gastrectomy samples from the BTUCH cohort among histological GC subtypes. **(A)** α-diversity was assessed using Richness, Shannon, and Simpson indices; statistical significance was determined using Pairwise Wilcoxon test (*p* < 0.05); **(B)** Non-metric Multidimensional Scaling (NMDS) plot illustrating the clustering patterns of the microbial communities grouped by GC histological subtypes; statistical significance was determined using PERMANOVA (*p* < 0.05); **(C)** Top 10 most abundant genera in gastrectomy grouped by GC histological subtypes; diffuse GC and intestinal GC.

To increase the power of the analyses, 52 tissues from the BTUCH cohort were added to the study, all corresponding to the GC group. When analyzing only the BTUCH cohort, no significant differences in α-diversity and *β*-diversity were observed neither between adjacent and tumoral tissues (Supplementary Figure 6) nor among GC stages (Supplementary Figure 7). Regarding histological intestinal and diffuse GC subtypes, even though no statistically significant differences were observed in α-diversity (Figure 5A), the NMDS ordination showed separation and PERMANOVA confirmed statistically significant differences between both histological subtypes (Figure 5B), indicating that each subtype is associated with a distinct microbial community. Genera such as *Escherichia*, *Clostridium*, *Parvimonas*, *Enterococcus*, and *Vagococcus* showed lower abundance in the diffuse subtype, whereas *Gemella*, *Haemophilus*, *Leptotrichia*, *Oribacterium*, *Neisseria*, *Campylobacter*, *Helicobacter*, *Hoylesella*, and *Lautropia* displayed higher abundance in diffuse tumors (Figure 5C and Supplementary Table 7).

**Figure 6:**
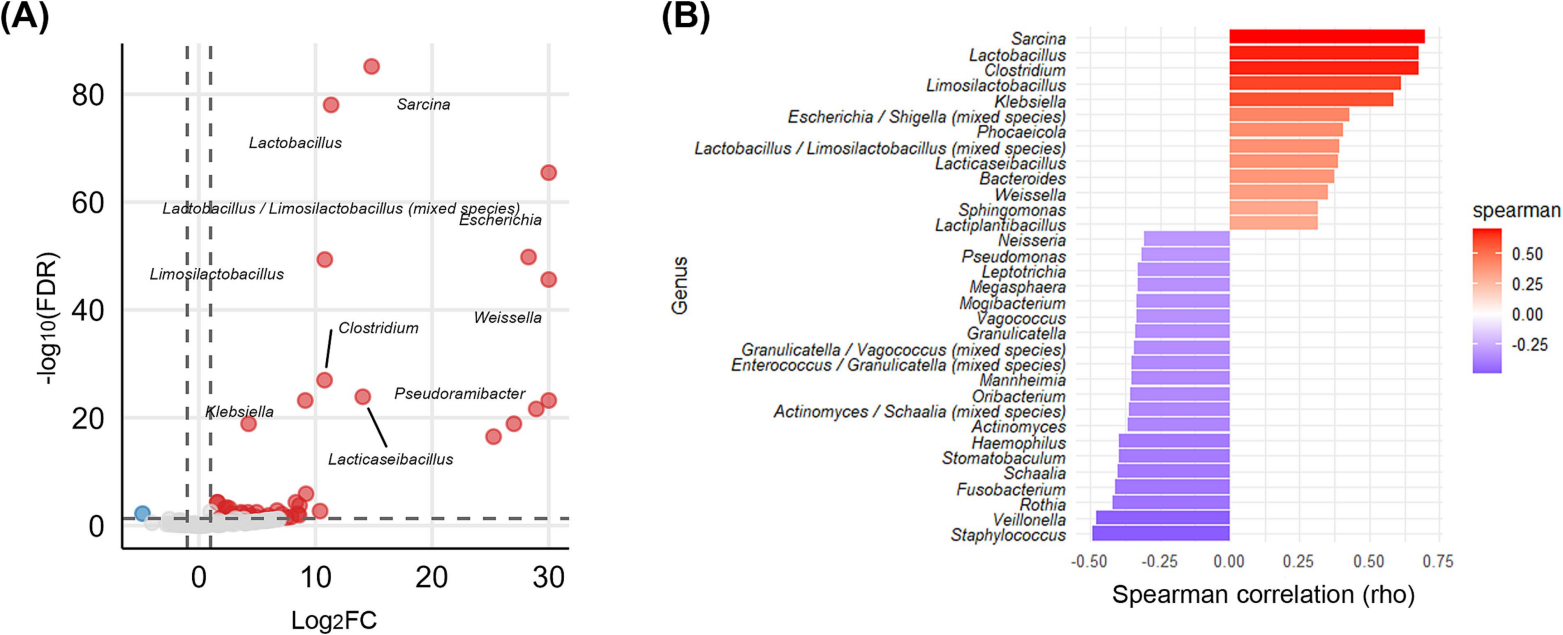
Genus-level microbial signatures associated with GC and disease progression. **(A)** Volcano plot between cancer and non-GC group from both MAGIC and BTUCH cohorts. The x-axis represents the log₂FC, while the y-axis shows the −log₁₀ of the adj. *p*-value. Genera with significant increases in abundance (log₂FC > 1, FDR < 0.05) are shown in red, and those significantly decreased (log₂FC < −1, FDR < 0.05) in blue; **(B)** Spearman correlations between genus-level relative abundance and histological stage. Bars represent the correlation coefficient (*ρ*) for each genus significantly associated with histology (adj. *p*-value <0.05, |*ρ*| ≥ 0.30). Positive values (red) indicate genera that increase with more advanced histological categories, whereas negative values (blue) correspond to genera that decrease through stages.

Furthermore, to explore microbial differences associated with GC in comparison with earlier stages of disease progression in the full cohort, a differential analysis between GC and non-GC groups was performed, comprising both biopsies and gastrectomies from MAGIC and BTUCH cohorts, providing a broader view of microbiome dynamics through various clinical and biological contexts. A volcano plot was generated to visualize differentially abundant bacterial genera between both groups using DESeq2 (Figure 6A), revealing distinct microbial patterns associated with GC, with strongly enriched genera in GC positioned on the right side of the plot, such as *Sarcina*, *Lactobacillus* and *Limosilactobacillus*. The strongest enrichment was observed for *Lactobacillus/Limosilactobacillus* (mixed species), *Escherichia*, *Weissella*, and *Pseudoramibacter*, all of which reached a log_2_FC of 30, indicating extremely elevated abundance in this comparison (Supplementary Table 8). Additionally, Spearman correlation analysis was performed between bacterial genus-level relative abundance and ordered progression stages, SG to GC, revealing a total of 34 genera biologically meaningful. Among them, genera such as *Sarcina*, *Lactobacillus*, *Clostridium*, and *Limosilactobacillus* and *Klebsiella* strongly increased through disease progression (|*ρ*| ≥ 0.58). In contrast, genera including *Staphylococcus*, *Veillonella*, *Rothia* and *Fusobacterium* exhibited moderate-to-strong negative correlations (|*ρ*| < −0.30), reflecting a reduction of these genera in progression stages prior to the emergence of GC (Figure 6B, Supplementary Table 9).

### 3.5 *Helicobacter-*related microbial signatures

**Figure 7:**
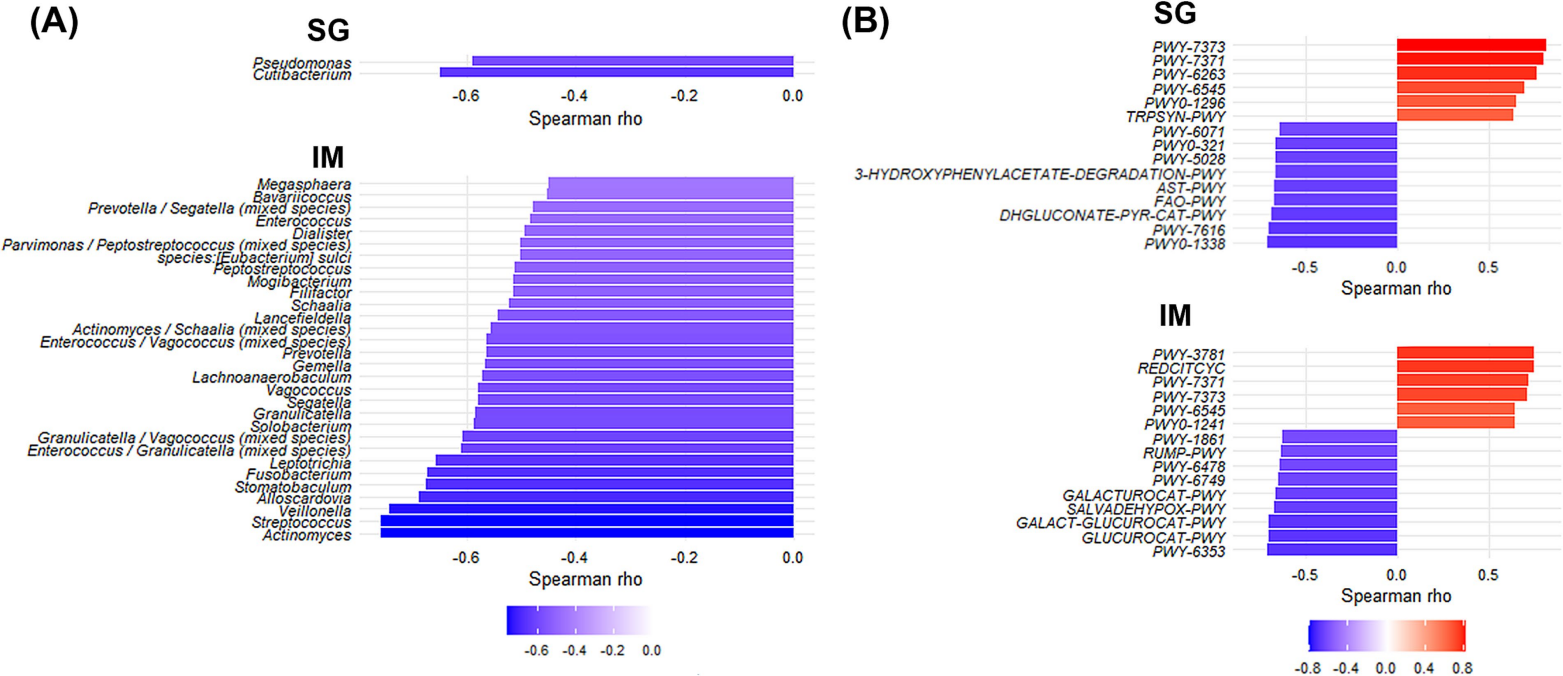
Spearman correlations between genus-level microbial abundance, and Helicobacter abundance. **(A)** Spearman correlations between genus relative abundance and *Helicobacter* abundance. Bars represent the correlation coefficient (ρ) for each genus within SG (top) and IM (bottom); **(B)** Spearman correlations between *Helicobacter* relative abundance and MetaCyc metabolic pathways listed on Supplementary Table 13. Bars represent the correlation coefficients (*ρ*) for the top 15 MetaCyc pathways within SG (top) and IM (bottom). Only genera meeting a prevalence threshold of ≥10% of samples within each stage were included in the analysis, adj. *p*-value< 0.05, |*ρ*| ≥ 0.30. Positive values (red) indicate a positive correlation with *Helicobacter* abundance, whereas negative values (blue) correspond to a negative correlation with *Helicobacter* abundance.

To explore the potential association of *Helicobacter* with the gastric microbiome along the Correa cascade, a targeted *Helicobacter*–centered analysis was performed in MAGIC and BTUCH cohorts together. To evaluate whether *Helicobacter* status influenced the gastric microbial community in diversity, tissues were stratified as *Helicobacter* positive (H+) or negative (H-) based on a relative abundance threshold of >1% for *Helicobacter* genera (H+) (Supplementary Table 10). Differential abundance testing was then performed comparing H + versus H– within progression groups. Groups with insufficient H + tissues as AG (H−, *n* = 8; H+, *n* = 0) or very limited sample size as DY (H−, *n* = 2; H+, *n* = 2) were excluded from this analysis. As expected, a higher microbiome *α*-diversity was observed in the H− group compared with the H + group in SG (H− = 20; H + = 9) and IM (H− = 17, H + = 11); and also, a clear separation of groups in the NMDS analysis in both comparisons (Supplementary Figure 8, 9) with a statistically significant difference. Among GC (H− = 38, H + = 31), there was no difference regarding *Helicobacter* status (Supplementary Figure 10). Also, a Spearman correlation analysis was performed to assess associations between the relative abundance of non-*Helicobacter* genera and *Helicobacter* abundance in early stages of the disease (SG and IM), identifying associated taxa potentially enriched at lower *Helicobacter* abundance. In SG, several genera showed significant correlations with *Helicobacter* abundance (FDR < 0.05). *Cutibacterium* and *Pseudomonas* exhibited strong negative correlations with *Helicobacter* relative abundance (*ρ* = −0.65 and −0.59), indicating that these genera are relatively enriched in tissues with low *Helicobacter* abundance and decrease as *Helicobacter* becomes more dominant (Figure 7, Supplementary Table 11). In contrast, no significant associations were detected in AG or GC after multiple-testing correction, suggesting a reduced or heterogeneous association of *Helicobacter* on microbial composition at these stages. IM exhibited significant *Helicobacter*–associated genera, where the most pronounced negative correlations were *Actinomyces, Streptococcus, Veillonella, Alloscardovia, Stomatobaculum* and *Fusobacterium,* all of which displayed consistent enrichment as *Helicobacter* abundance decreased (Figure 7, Supplementary Table 12).

Given the *Helicobacter*-associated microbial restructuring observed at the SG and IM stages, a functional pathways analysis using PICRUSt2 and LEfSe was performed to identify functional signatures related to *Helicobacter* abundance based on the MetaCyc database. Next, a Sperman correlation analysis was performed to evaluate the importance of these pathways in relation to *Helicobacter* relative abundance. For SG, pathways negatively correlated were predominantly related to degradation and utilization processes and pathways positively correlated were mainly associated with biosynthetic functions. As for IM, pathways negatively correlated were mainly associated with degradation and utilization processes and pathways positively correlated predominantly involved central energy metabolism as well as multiple biosynthetic routes (Figure 7, Supplementary Table 13).

## 4 Discussion

GC is a multifactorial disease arising from the interplay of host, microbial and environmental factors. Dysbiosis has been associated with the promotion of chronic inflammation and increasing susceptibility to cancer development (Noto and RM, 2017), highlighting the importance of functional shifts that might be responsible for the development of gastric diseases (Waskito et al., 2022). Studying microbial shifts through cancer progression is crucial to understand the pathogenesis related to early microbial changes that occur during gastric carcinogenesis. This study provides a comprehensive characterization of the gastric microbiome through different stages of the Correa cascade, histology subtypes, and bacterial taxa associated with *Helicobacter* abundance in gastric tissues from the Chilean MAGIC and BTUCH cohorts. In biopsy tissues the most prevalent genera included *Streptococcus, Gemella, Granulicatella, Haemophilus, Helicobacter, Prevotella, Rothia, Staphylococcus,* and *Veillonella*. This microbial profile is consistent with previously reported gastric microbiota compositions described in independent cohorts (Waskito et al., 2022; Li et al., 2009; Bik et al., 2006). Our results highlight a progressive restructuring of the gastric microbiota along the carcinogenesis cascade in biopsy tissues, characterized by stage-specific differences in genus level abundance, with an important shift in the microbial community structure in GC compared with other stages as illustrated by the NMDS analysis. Microbial dynamics associated with GC also included gastrectomy tissues, pointing to clear dysbiosis related to non-GC tissues, with an increase of *Lactobacillus* and *Limosilactobacillus*, which was further validated in the BTUCH cohort. Usually, *Lactobacillus* enrichment in GC is related to the gradual reduction of gastric secretion and the increase of pH that come with carcinogenesis creating new conditions to overgrow (Rabenhorst et al., 2024). As the disease progressed from SG to GC, mentioned genera strongly increased, together with genera such as *Sarcina, Clostridium* and *Klebsiella.* These genera have frequently been reported in GC tissues and have been associated with GC development (Hsieh et al., 2018; Kim et al., 2025; Park et al., 2022; Dai et al., 2021; Dias-Jácome et al., 2016). Moreover, significant differences through histological subtypes were observed, in agreement with molecular tumor heterogeneity and supporting the potential utility of microbial signatures as complementary biomarkers associated with GC (Liu et al., 2022).

As expected, *Helicobacter* analysis demonstrated that H- tissues have a higher diversity in early stages. Notably, reduced *Helicobacter* abundance was associated with the enrichment of other genera, including *Cutibacterium* and *Pseudomonas* in SG and genera such as *Actinomyces, Streptococcus, Veillonella* in IM, in agreement with prior evidence describing *Helicobacter* as a major modulator of microbiome changes (Spiegelhauer et al., 2020; Niikura et al., 2023). In both SG and IM conditions, pathways negatively correlated with *Helicobacter* abundance were predominantly related to degradation and utilization processes, such as methanol and glucose oxidation, fatty acid *β*-oxidation, and the degradation of amino acids and aromatic compounds in SG, and carbohydrate and nucleotide degradation and formaldehyde metabolism in IM. These associations suggest that potential microbial shifts may reflect predicted metabolic changes associated with GC (Choi et al., 2024; Yu et al., 2024; Zhu et al., 2025).

Our study, based on full-length 16S rRNA sequencing using nanopore technology, underscores the importance of expanding microbiome research to diverse populations, showing that underrepresented regions may harbor unique microbial signatures and potentially novel disease biomarkers (Abdill et al., 2025). A major strength of this work lies in the inclusion of two independent cohorts, MAGIC and BTUCH, derived from distinct geographic regions in Chile. The MAGIC cohort comprises patients from Punta Arenas in southern Chile, enabling detailed assessment through multiple precancerous and cancer stages, whereas the BTUCH cohort includes GC cases from Santiago de Chile, providing an independent, cancer-focused dataset for validation. Santiago, the capital of Chile, is one of the largest cities in South America, and it is highly urbanized with more than 7 million inhabitants (ArcGIS, 2024). Punta Arenas is in the southernmost part of Chile, known as the main urban center of the Chilean Patagonia and the “gateway” to Antarctica with 132 thousand inhabitants with only 74% living in the urbanized zone (ArcGIS, 2024; Instituto Nacional De Estadísticas, 2019). Differences in microbial profiles observed between cohorts may reflect geographic separation, regional dietary habits, and environmental or clinical factors. Nevertheless, the consistent enrichment of key cancer-associated genera in both datasets supports the robustness and reproducibility of our findings. Importantly, the inclusion of these cohorts contributes valuable data from Latin American populations and helps to address the underrepresentation of world-wide cohorts in gastric microbiome research. Also, the inclusion of premalignant lesions in our study is particularly relevant, as it suggests early microbial changes preceding cancer development and provides insights into the progressive shifts in the gastric microbiome along the Correa cascade. This is important not only for clarifying potential microbial contributions to gastric carcinogenesis but also because taxa consistently enriched across cohorts may serve as candidate biomarkers for early detection or disease monitoring. These findings may help to identify microbial signatures associated with GC progression that could complement current diagnostic and risk stratification strategies. However, potential confounding factors may contribute to the observed microbial differences through stages.

While these findings provide valuable insights into gastric microbial dynamics, limitations in the study should be considered when interpreting the results. Technical constraints related to sequencing depth and taxonomic assignment affected sample retention through diagnostic groups, particularly in low-biomass gastric tissues where some low-abundance or poorly represented taxa may remain difficult to classify using current reference databases. In addition, the relatively modest sample size of some progression groups may have limited our ability to detect subtler stage-specific differences. Nevertheless, due to the specific demographic origin of the cohort studies, validation in larger and independent populations should be necessary to determine the extended biological relevance of these signatures associated with GC.

## 5 Conclusion

In conclusion, our findings demonstrate that gastric carcinogenesis is accompanied by dynamic restructuring of the gastric microbiota through disease progression and histological subtypes of GC. Using full-length 16S rRNA sequencing in a Chilean cohort, we highlight non-*Helicobacter* genera as candidate microbial biomarkers associated with gastric disease and cancer progression, underscoring their relevance in an underrepresented population. Future studies including larger sample sizes, cross-population validation, and metabolomic approaches will be important to further validate the biological relevance of these microbial signatures and their potential as biomarkers in GC.

## Data Availability

The original contributions presented in the study are publicly available. This data can be found here: https://www.ncbi.nlm.nih.gov/bioproject/PRJNA1434549.
