# Supplementary material for: Microbial dynamics in gastric cancer: insights from full-length 16S rRNA nanopore sequencing in the MAGIC cohort
Source: Front Microbiol. 2026 Jun 9;17:1841026. doi: 10.3389/fmicb.2026.1841026 (PMC13287022; doi:10.3389/fmicb.2026.1841026)
Supplement: Supplementary file 1 [file Supplementary_file_1.DOCX]

Supplementary Material

**1 Supplementary Figures and Tables**

**1.1 Supplementary figures**

**Supplementary figure 1.**

**
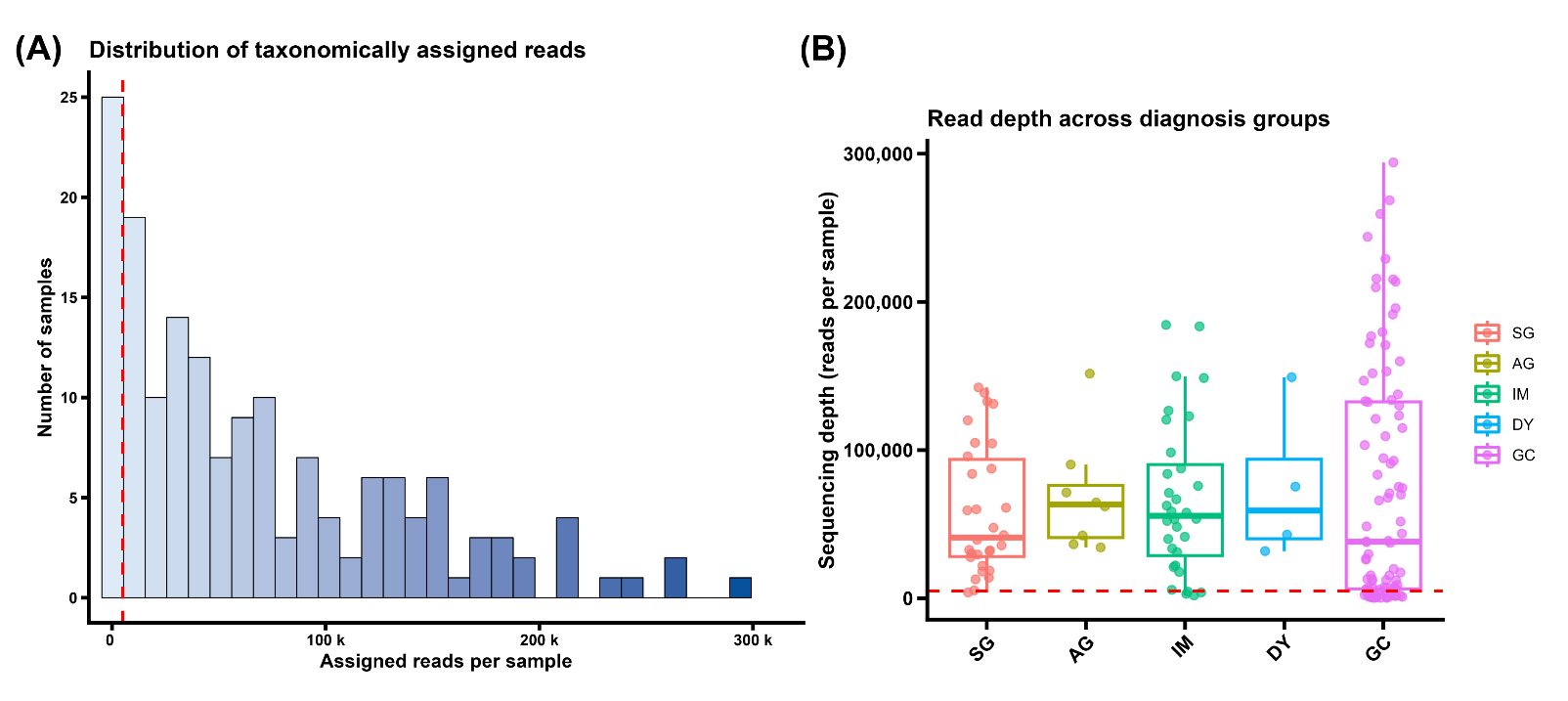
Supplementary Figure 1. Sequencing depth distribution and read counts by diagnostic category.** (A) Histogram showing the distribution of assigned reads per sample across all datasets. (B) Bar plot showing the total number of assigned reads aggregated by diagnostic category. Line in red in both graphs represents the cut-off of ~5,000 assigned reads. SG = Superficial Gastritis, AG = Atrophic Gastritis, IM = Intestinal Metaplasia, DY = Dysplasia, GC = Gastric Cancer.

**Supplementary figure 2.**


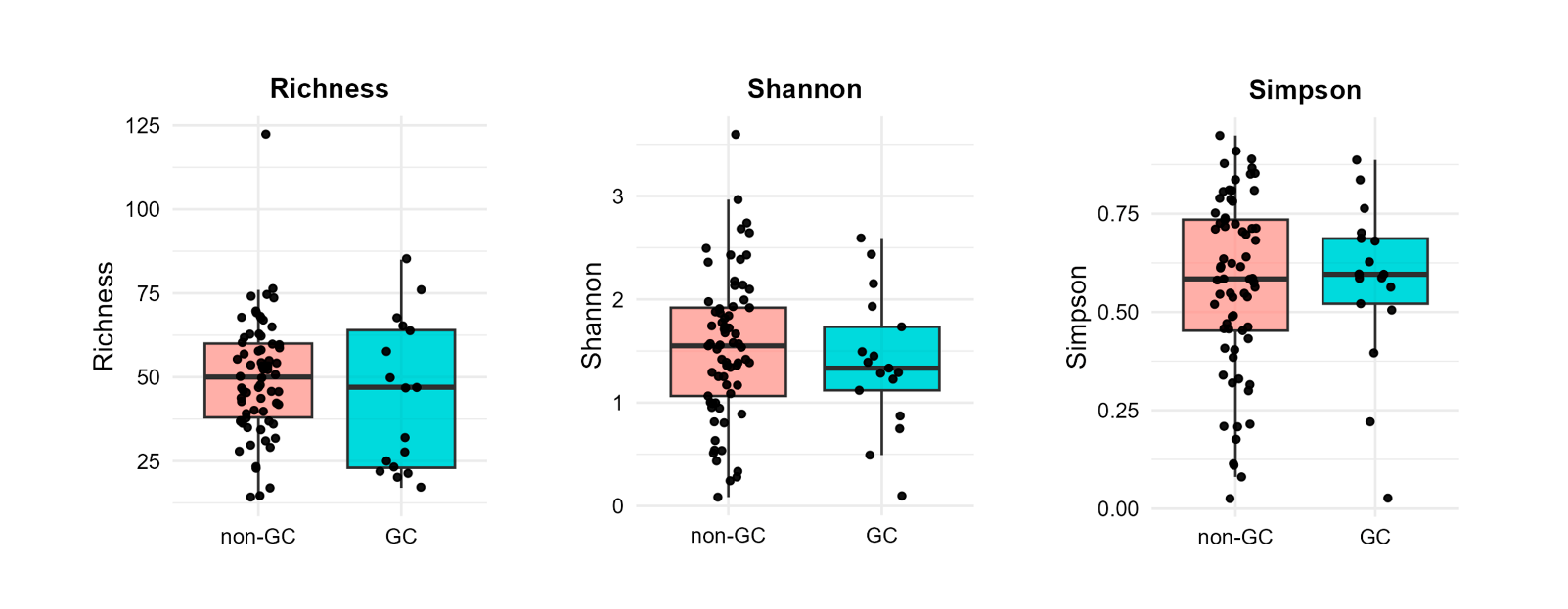


**Supplementary Figure 2. α- and β-diversity metrics in biopsy tissues from the MAGIC cohort by stomach site. (A)** α-diversity was assessed using Richness, Shannon, and Simpson indices. Statistical significance was determined using Pairwise Wilcoxon test (p < 0.05); **(B)** Top 10 most abundant genera in biopsies grouped in antrum and body; **(C)** Non-metric Multidimensional Scaling (NMDS) plot illustrating the clustering patterns of the microbial communities in biopsies grouped in antrum and body. Statistical significance was determined using PERMANOVA (p < 0.05).

**Supplementary figure 3.**
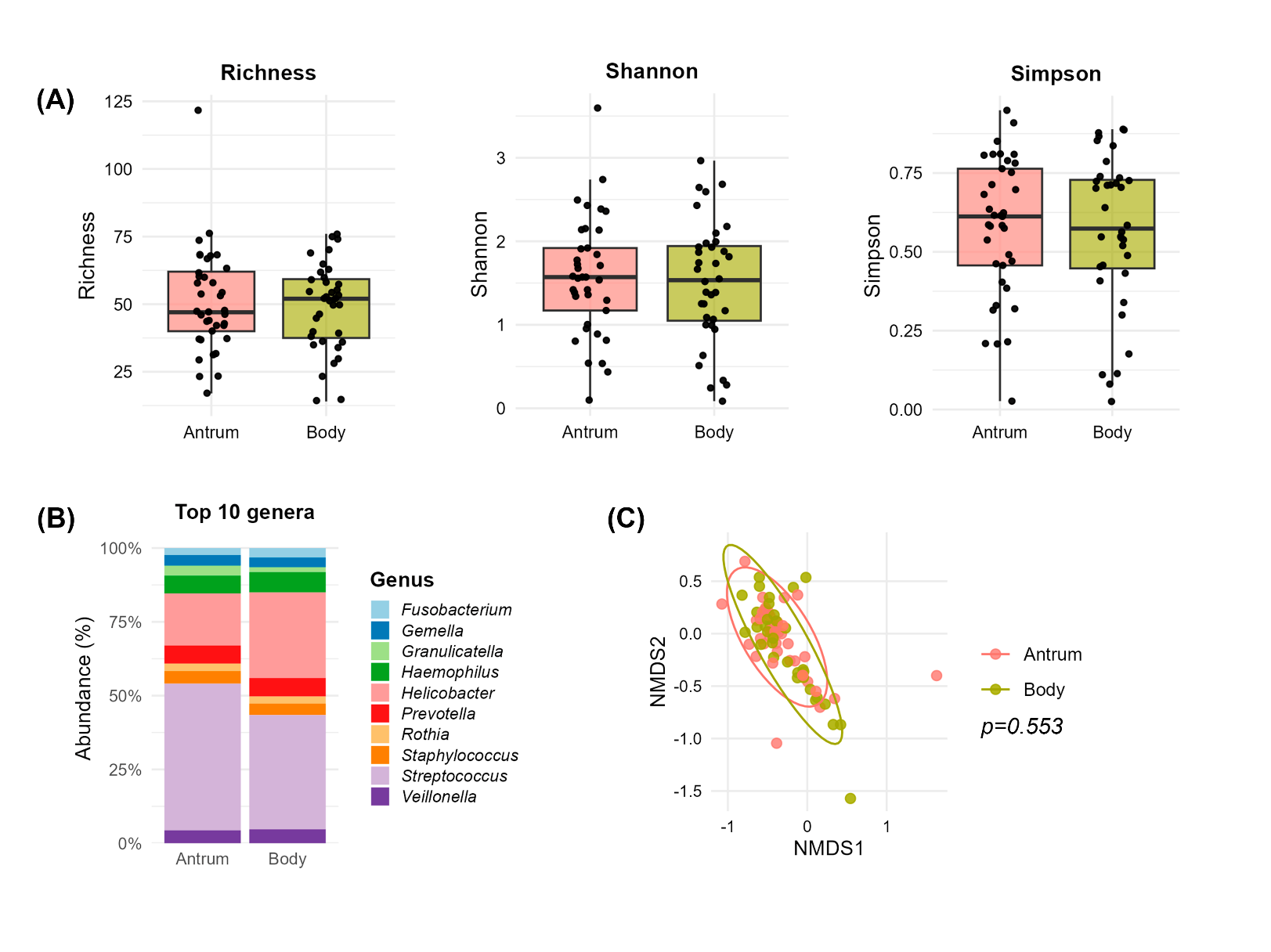


**Supplementary Figure 3. α-diversity metrics in biopsy tissues from the MAGIC cohort by antrum and body.** α-diversity was assessed using Richness, Shannon, and Simpson indices. Statistical significance was determined using Pairwise Wilcoxon test (p < 0.05).

**Supplementary figure 4.**


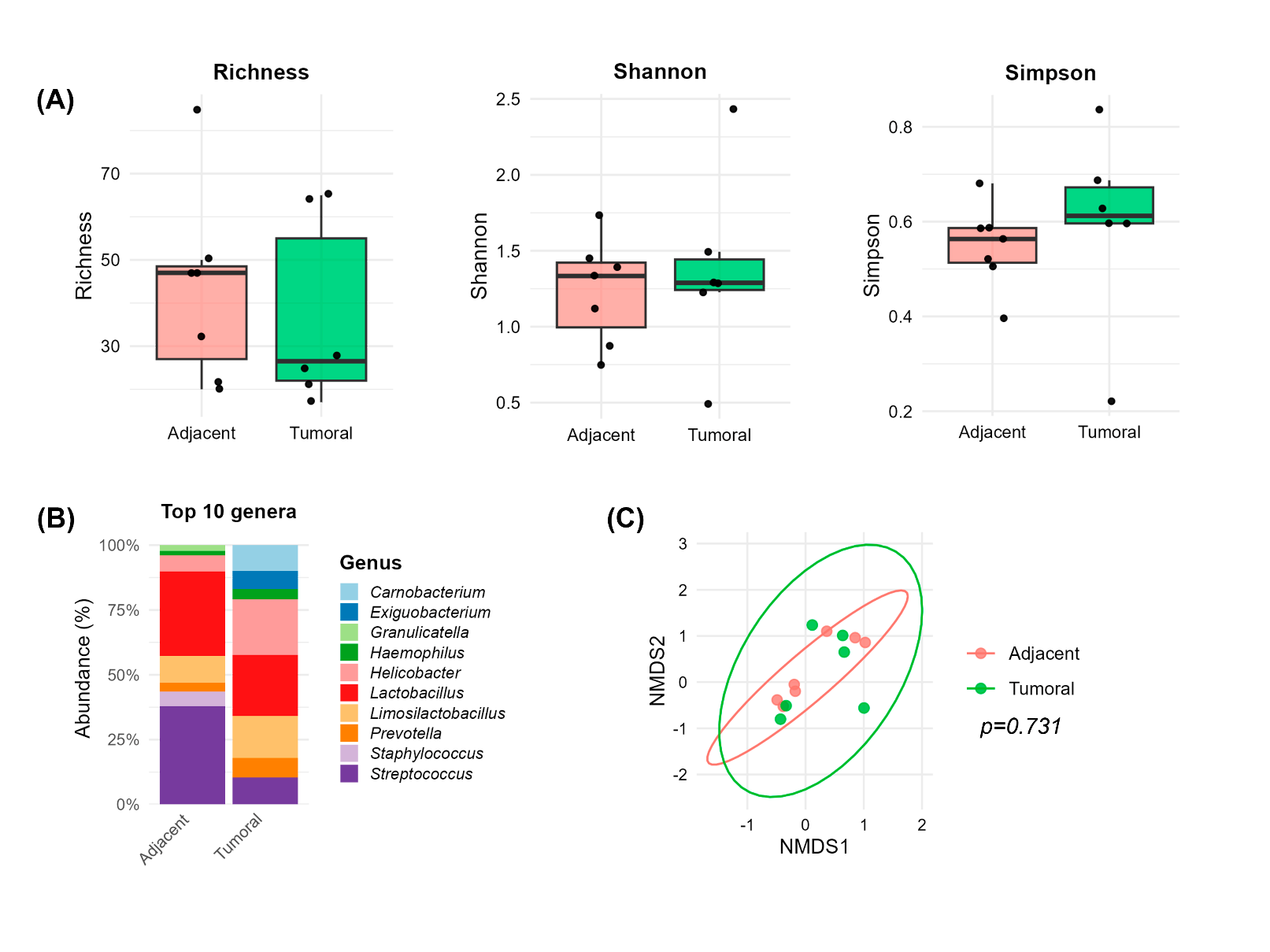


**Supplementary Figure 4.** **α- and β-diversity metrics in GC gastrectomy tissues by adjacent or tumoral tissue from the MAGIC cohort.** **(A)** α-diversity was assessed using Richness, Shannon, and Simpson indices. Statistical significance was determined using Pairwise Wilcoxon test (p < 0.05); **(B)** Top 10 most abundant genera in tissues by adjacent and tumoral tissue **(C)** Non-metric Multidimensional Scaling (NMDS) plot illustrating the clustering patterns of the microbial communities in gastrectomies by adjacent and tumoral tissue. Statistical significance was determined using PERMANOVA (p < 0.05).

**Supplementary figure 5.**


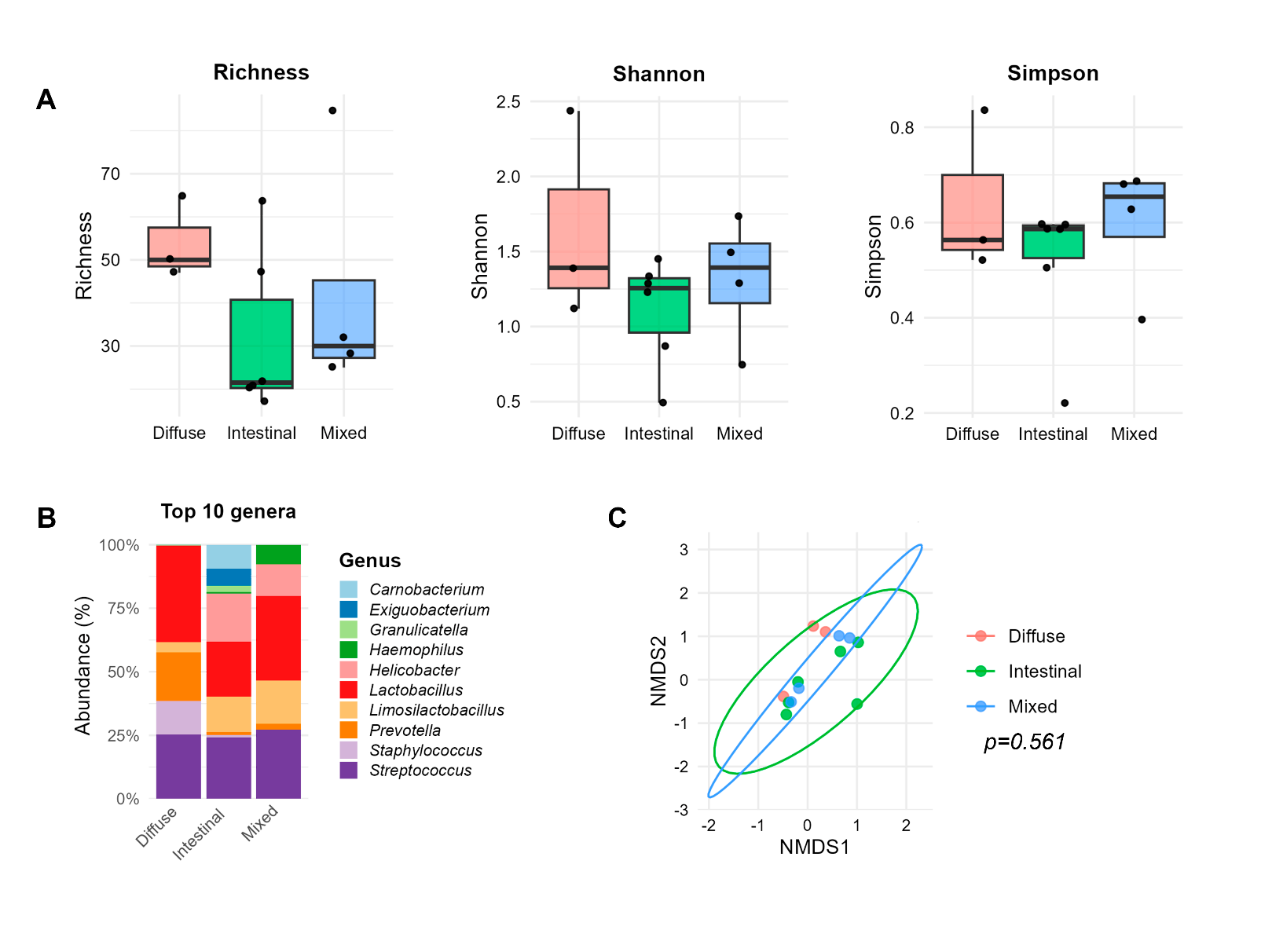


**Supplementary Figure 5. α- and β-diversity metrics in GC gastrectomy tissues by histology from the MAGIC cohort. (A)** α-diversity was assessed using Richness, Shannon, and Simpson indices. Statistical significance was determined using Pairwise Wilcoxon test (p < 0.05); **(B)** Top 10 most abundant genera in tissues grouped by histology **(C)** Non-metric Multidimensional Scaling (NMDS) plot illustrating the clustering patterns of the microbial communities in tissues grouped by histology. Statistical significance was determined using PERMANOVA (p < 0.05).

Supplementary figure 6.


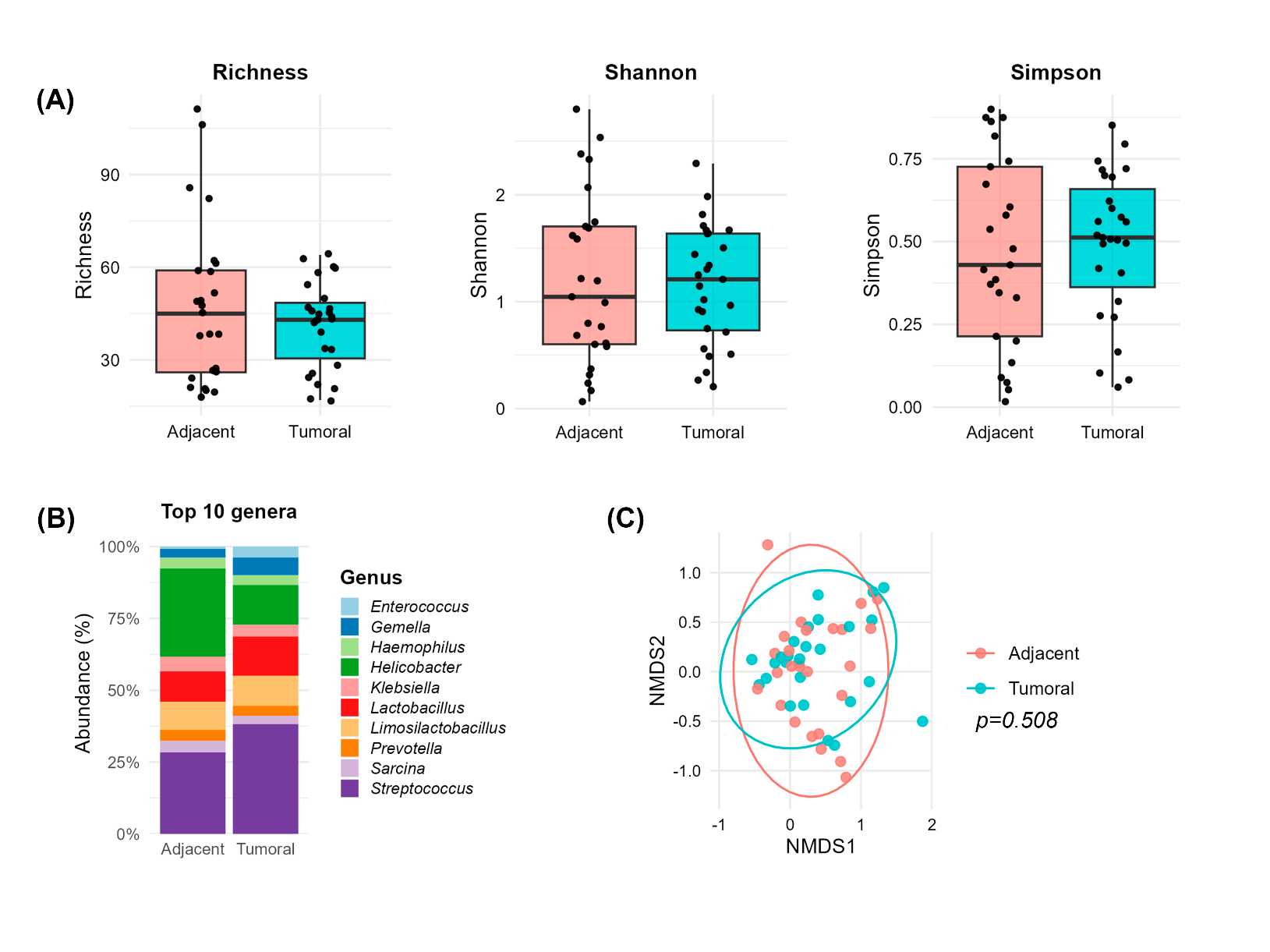


**Supplementary Figure 6. α- and β-diversity metrics in GC gastrectomy tissues by adjacent or tumoral tissue from the BTUCH cohort. (A)** α-diversity was assessed using Richness, Shannon, and Simpson indices. Statistical significance was determined using Pairwise Wilcoxon test (p < 0.05); **(B)** Top 10 most abundant genera in tissues grouped by adjacent or tumoral tissue; **(C)** Non-metric Multidimensional Scaling (NMDS) plot illustrating the clustering patterns of the microbial communities in tissues grouped by adjacent or tumoral tissue. Statistical significance was determined using PERMANOVA (p < 0.05).

Supplementary figure 7.


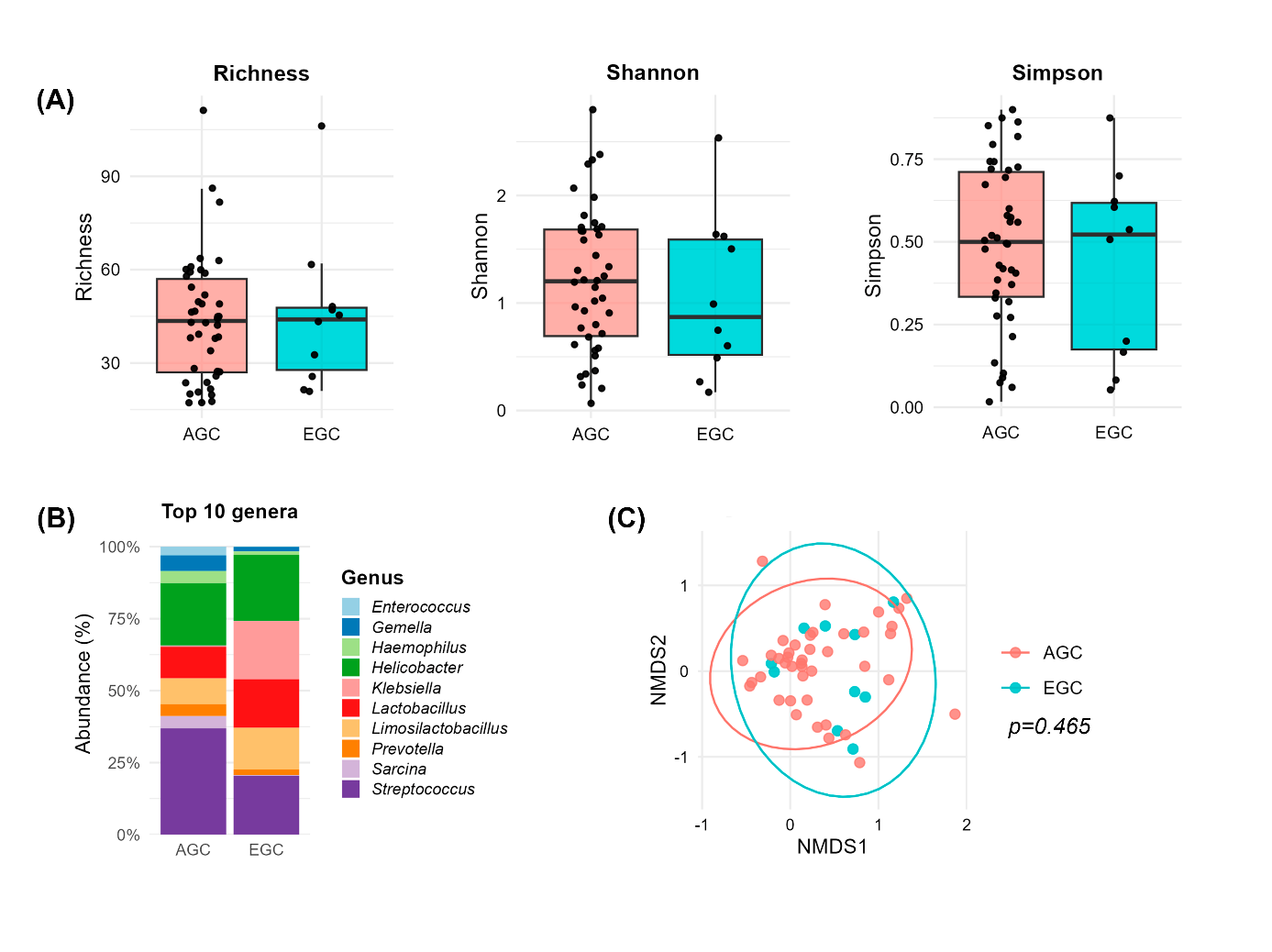


**Supplementary Figure 7. α- and β-diversity metrics in gastrectomy tissues by GC progression from the BTUCH cohort**. **(A)** α-diversity was assessed using Richness, Shannon, and Simpson indices. Statistical significance was determined using Pairwise Wilcoxon test (p < 0.05). **(B)** Top 10 most abundant genera in tissues grouped by progression stage; **(C)** Non-metric Multidimensional Scaling (NMDS) plot illustrating the clustering patterns of the microbial communities in biopsies grouped by progression stage. Statistical significance was determined using PERMANOVA (p < 0.05). AGC = Advanced gastric cancer, EGC = early gastric cancer.

Supplementary figure 8.


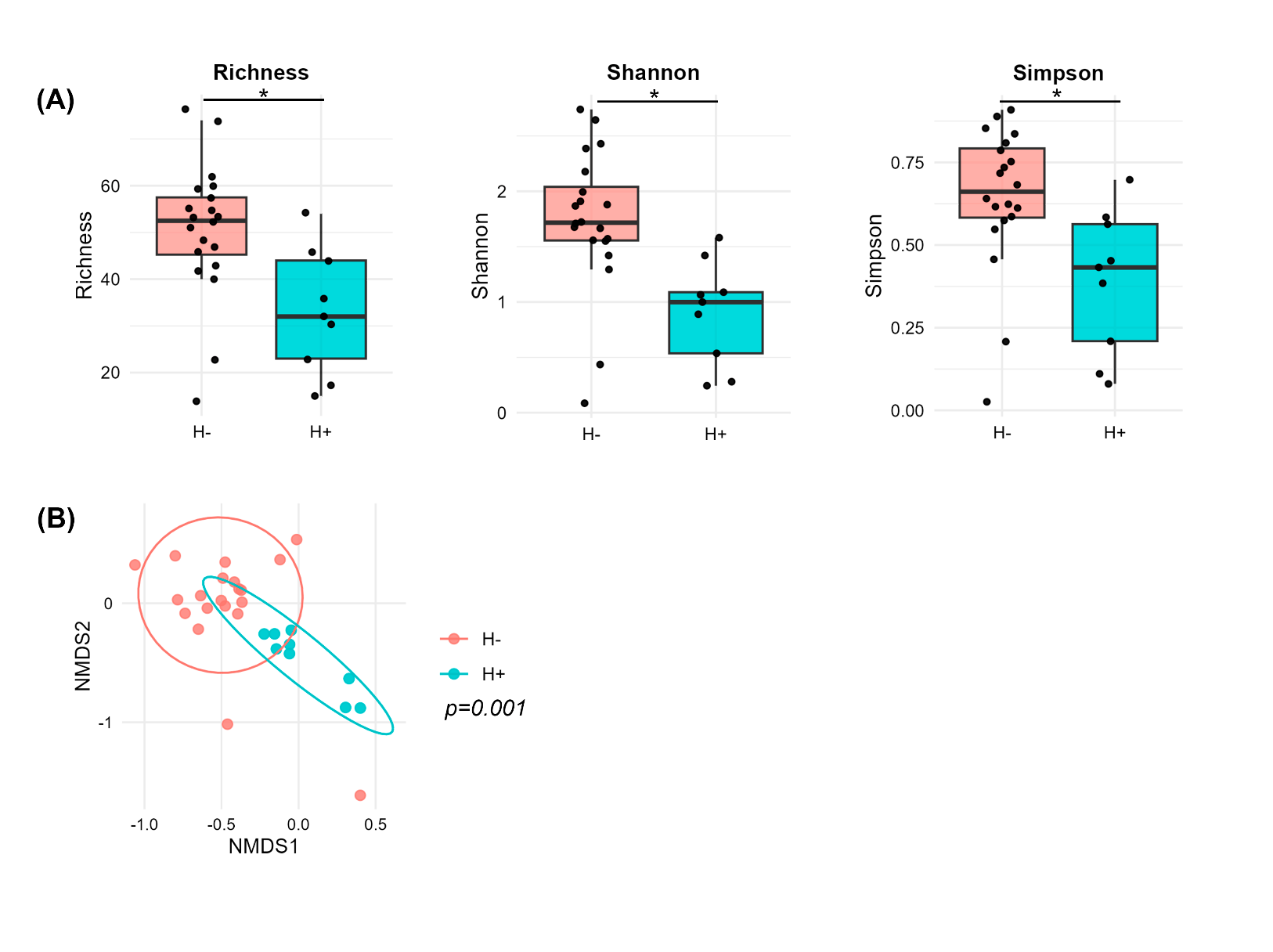


**Supplementary Figure 8. α- and β-diversity metrics in H- and H+ tissues in SG. (A)** α-diversity was assessed in SG using Richness, Shannon, and Simpson indices in H- and H+ tissues. Statistical significance in α diversity was determined using Pairwise Wilcoxon test (p < 0.05); **(B)** Non-metric Multidimensional Scaling (NMDS) plot illustrating the clustering patterns of the microbial communities in H- and H+ tissues. Statistical significance in NMDS was determined using PERMANOVA (p < 0.05). SG = Superficial gastritis.

Supplementary figure 9.


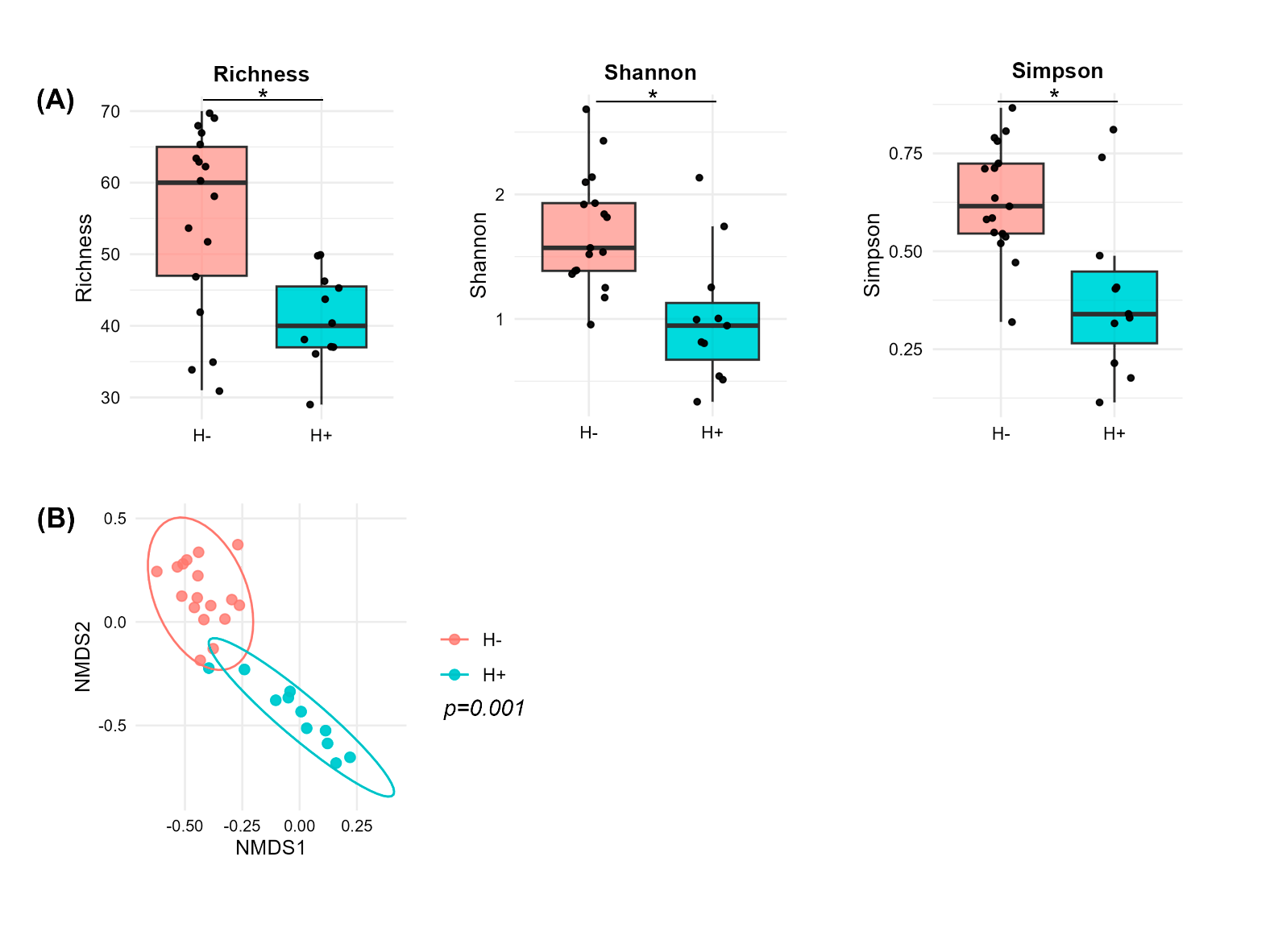


**Supplementary Figure 9. α- and β-diversity metrics in H- and H+ tissues in IM.** **(A)** α-diversity was assessed in IM using Richness, Shannon, and Simpson indices in H- and H+ tissues. Statistical significance in α diversity was determined using Pairwise Wilcoxon test (p < 0.05); **(B)** Non-metric Multidimensional Scaling (NMDS) plot illustrating the clustering patterns of the microbial communities in H- and H+ tissues. Statistical significance in NMDS was determined using PERMANOVA (p < 0.05). IM = Intestinal Metaplasia.

Supplementary figure 10.


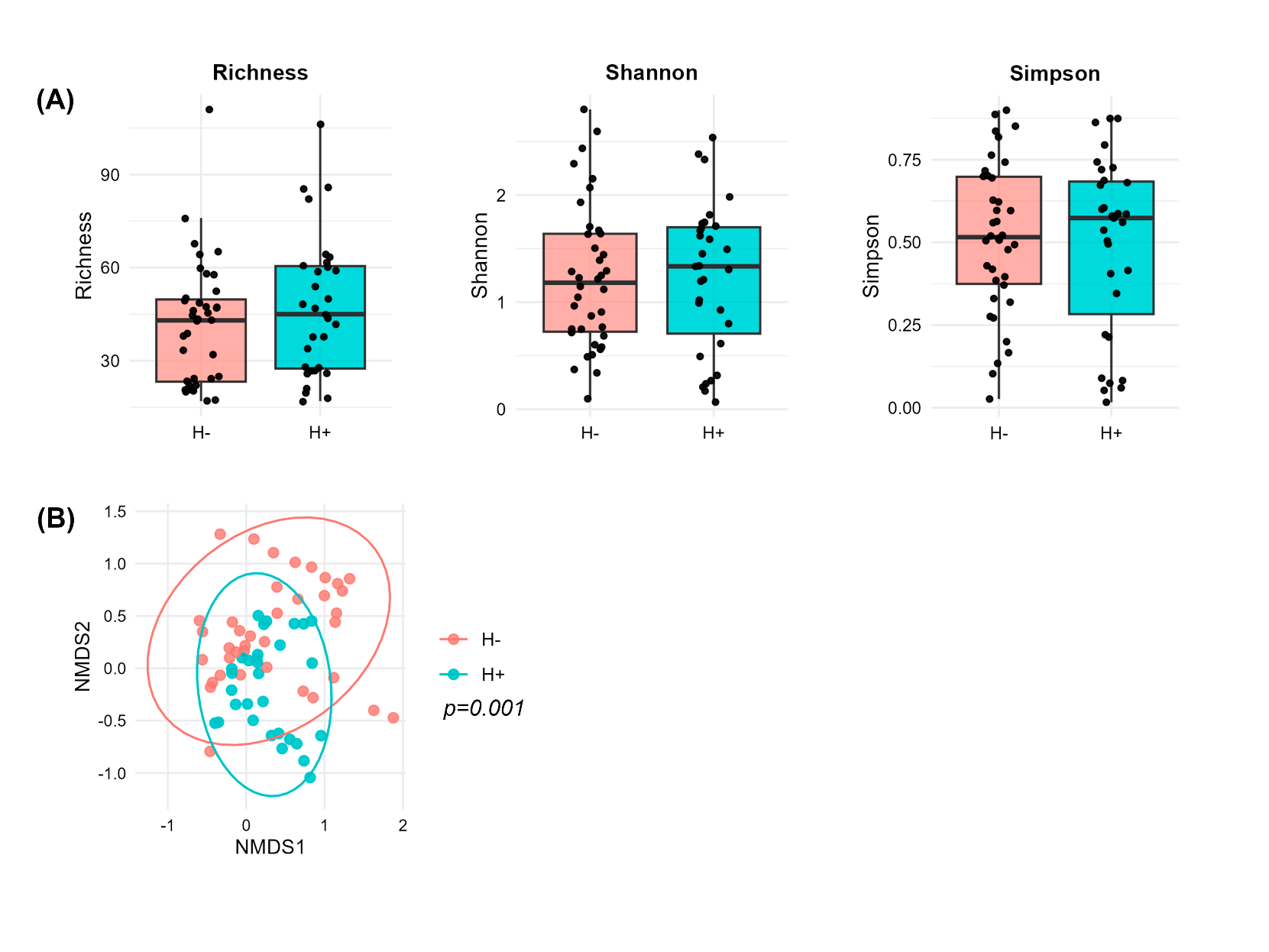


**Supplementary Figure 10. α- and β-diversity metrics in H- and H+ tissues in GC. (A)** α-diversity was assessed in GC using Richness, Shannon, and Simpson indices in H- and H+ tissues. Statistical significance in α diversity was determined using Pairwise Wilcoxon test (p < 0.05); **(B)** Non-metric Multidimensional Scaling (NMDS) plot illustrating the clustering patterns of the microbial communities in HP- and HP+ tissues. Statistical significance in NMDS was determined using PERMANOVA (p < 0.05). GC = Gastric Cancer.

**1.2 Supplementary tables**

**Supplementary Table 1. Sequencing coverage and read quality statistics for each sample included in the study.**

| **sample** | **num_seqs** | **avg_len** | **N50** | **AvgQual** | **num_seqs_filtered** | **num_seqs_assigned** |
| --- | --- | --- | --- | --- | --- | --- |
| 51519N | 843172 | 1225 | 1609 | 17.67 | 248145 | 7068 |
| 51519T | 739322 | 1256 | 1611 | 17.73 | 218785 | 6309 |
| 51525N | 45569 | 1137 | 1555 | 16.96 | 7969 | 1135 |
| 51525T | 77104 | 1200 | 1606 | 17.36 | 19117 | 1832 |
| 51631N | 890753 | 935 | 1510 | 17.66 | 212797 | 83386 |
| 51631T | 1192237 | 1313 | 1613 | 17.78 | 382530 | 38330 |
| 51637N | 127605 | 985 | 1536 | 17.63 | 31504 | 17435 |
| 51637T | 784639 | 1298 | 1536 | 17.94 | 312663 | 229049 |
| 51638N | 1320294 | 1299 | 1614 | 17.95 | 409936 | 3408 |
| 51638T | 888428 | 1362 | 1614 | 17.69 | 277037 | 6444 |
| 51646N | 600731 | 730 | 1412 | 17.90 | 98272 | 26089 |
| 51646T | 628725 | 1032 | 1521 | 18.35 | 244235 | 294148 |
| 51655N | 1620597 | 1214 | 1524 | 17.74 | 527126 | 159916 |
| 51655T | 1036443 | 1268 | 1563 | 17.71 | 345092 | 74431 |
| 51659N | 1061187 | 918 | 1603 | 17.71 | 222742 | 1699 |
| 51659T | 961180 | 1261 | 1615 | 17.65 | 284868 | 12986 |
| 51660N | 831014 | 1222 | 1503 | 17.72 | 277753 | 103413 |
| 51660T | 1189912 | 1193 | 1568 | 17.63 | 358298 | 75300 |
| 51661N | 233447 | 1306 | 1534 | 17.92 | 98760 | 90878 |
| 51661T | 865045 | 1316 | 1535 | 17.88 | 338061 | 215690 |
| 51712N | 778802 | 1349 | 1525 | 17.75 | 282784 | 92817 |
| 51712T | 893264 | 1249 | 1560 | 17.69 | 277645 | 51796 |
| 5171N | 491107 | 1221 | 1512 | 17.98 | 205281 | 195810 |
| 5171T | 684830 | 836 | 1511 | 17.95 | 188044 | 172172 |
| 5172N | 1148184 | 1171 | 1603 | 17.77 | 321426 | 7352 |
| 5172T | 732716 | 1242 | 1609 | 17.89 | 223985 | 6793 |
| 5173N | 2491797 | 849 | 1500 | 17.80 | 553180 | 191601 |
| 5173T | 104947 | 987 | 1506 | 17.92 | 32833 | 26777 |
| 5174N | 782607 | 1244 | 1599 | 17.97 | 234129 | 2240 |
| 5174T | 1870166 | 692 | 1476 | 17.95 | 289763 | 37386 |
| 51768N | 982365 | 1141 | 1493 | 17.78 | 310083 | 153179 |
| 51768T | 1005467 | 1164 | 1481 | 17.92 | 366745 | 259341 |
| 51771T | 506219 | 1189 | 1520 | 17.84 | 180218 | 133009 |
| 5177N | 1478298 | 1033 | 1558 | 17.96 | 404868 | 70878 |
| 5177T | 552228 | 1311 | 1598 | 17.80 | 195624 | 43660 |
| 51873N | 686825 | 1213 | 1467 | 17.97 | 252499 | 176871 |
| 51873T | 990621 | 1297 | 1612 | 17.72 | 307978 | 9030 |
| 51874N | 439333 | 1181 | 1465 | 17.50 | 155453 | 133774 |
| 51874T | 459067 | 767 | 1531 | 17.81 | 76051 | 5831 |
| 51989N | 813007 | 1119 | 1607 | 17.79 | 208520 | 15559 |
| 51989T | 495044 | 922 | 1616 | 17.81 | 97310 | 1592 |
| 51991N | 1578130 | 694 | 1557 | 17.85 | 242593 | 12096 |
| 51991T | 884720 | 726 | 1530 | 17.99 | 170757 | 37980 |
| 51992N | 793932 | 1238 | 1466 | 17.98 | 308072 | 213682 |
| 51992T | 1397629 | 1047 | 1603 | 17.65 | 346507 | 1897 |
| 51993N | 1299218 | 1090 | 1581 | 17.67 | 334532 | 3838 |
| 520103N | 1178380 | 1152 | 1511 | 17.82 | 356549 | 109418 |
| 520103T | 796643 | 1082 | 1511 | 17.64 | 217440 | 48549 |
| 520104N | 967605 | 1079 | 1602 | 17.67 | 242628 | 11633 |
| 520104T | 1327031 | 918 | 1602 | 17.70 | 281928 | 15497 |
| 52110N | 1098027 | 1234 | 1611 | 17.80 | 332941 | 6375 |
| 52110T | 886393 | 1275 | 1511 | 17.72 | 307202 | 146853 |
| 5213N | 1260238 | 1012 | 1550 | 17.74 | 317582 | 69948 |
| 5213T | 706667 | 1144 | 1603 | 17.57 | 196505 | 19805 |
| 5214N | 424315 | 1240 | 1502 | 18.14 | 171330 | 130154 |
| 5214T | 311862 | 1302 | 1501 | 18.19 | 147574 | 137605 |
| 5219N | 1465875 | 912 | 1534 | 17.86 | 383347 | 268607 |
| 5219T | 1060330 | 1109 | 1612 | 17.71 | 273826 | 12286 |
| 5220006N | 539156 | 1266 | 1464 | 18.14 | 247454 | 243960 |
| 5220006T | 1565366 | 1091 | 1603 | 17.81 | 407167 | 29813 |
| GC0001N | 1713654 | 876 | 1613 | 18.87 | 497868 | 2533 |
| GC0001T | 789529 | 801 | 1589 | 16.69 | 182626 | 1019 |
| GC0026A | 544314 | 1396 | 1612 | 16.66 | 239110 | 31703 |
| GC0029A | 686327 | 1346 | 1499 | 16.53 | 328267 | 71430 |
| GC0029C | 705429 | 1309 | 1606 | 16.52 | 288999 | 42405 |
| GC0041A | 666550 | 1521 | 1642 | 16.71 | 295163 | 27761 |
| GC0041C | 516634 | 1481 | 1610 | 16.63 | 249839 | 59359 |
| GC0046A | 807364 | 1329 | 1596 | 18.76 | 427104 | 75387 |
| GC0046C | 577086 | 1352 | 1475 | 19.04 | 338814 | 149221 |
| GC0055A | 748832 | 1302 | 1580 | 18.91 | 394897 | 62497 |
| GC0055C | 649367 | 1270 | 1467 | 19.10 | 377774 | 183526 |
| GC0095A | 636208 | 1245 | 1508 | 19.02 | 340771 | 122930 |
| GC0095C | 598723 | 1138 | 1508 | 18.86 | 291035 | 120544 |
| GC0116A | 610572 | 1164 | 1508 | 18.96 | 302426 | 138633 |
| GC0116C | 355793 | 1112 | 1466 | 19.00 | 171638 | 87460 |
| GC0140A | 537868 | 1221 | 1585 | 16.61 | 205407 | 34377 |
| GC0140C | 494158 | 1303 | 1526 | 16.71 | 217543 | 62093 |
| GC0156A | 565257 | 1335 | 1474 | 19.08 | 323888 | 142287 |
| GC0156C | 204094 | 1238 | 1515 | 20.01 | 135099 | 120110 |
| GC0157A | 368014 | 1247 | 1515 | 18.93 | 184557 | 48231 |
| GC0157C | 492621 | 1223 | 1517 | 18.98 | 236301 | 41550 |
| GC0159A | 332480 | 1053 | 1465 | 19.26 | 172012 | 126592 |
| GC0159C | 266218 | 1112 | 1465 | 19.11 | 134962 | 83984 |
| GC0163A | 268723 | 1257 | 1468 | 19.15 | 169867 | 104497 |
| GC0163C | 325227 | 1289 | 1466 | 19.23 | 205498 | 131233 |
| GC0165N | 1716360 | 397 | 442 | 17.29 | 85214 | 13015 |
| GC0165T | 314009 | 1275 | 1507 | 19.17 | 173004 | 121084 |
| GC0166A | 409017 | 1303 | 1509 | 18.79 | 229663 | 95826 |
| GC0166C | 417743 | 1285 | 1508 | 18.88 | 238501 | 104971 |
| GC0167A | 405654 | 1168 | 1533 | 18.72 | 182603 | 30309 |
| GC0173A | 576056 | 1081 | 1503 | 18.86 | 250096 | 71160 |
| GC0173C | 875634 | 981 | 1503 | 18.93 | 364328 | 148698 |
| GC0194A | 368970 | 1325 | 1466 | 19.24 | 241297 | 149890 |
| GC0194C | 378338 | 1346 | 1465 | 19.36 | 266137 | 184494 |
| GC0206A | 408645 | 1194 | 1503 | 19.02 | 239120 | 151625 |
| GC0206C | 410380 | 1124 | 1501 | 18.90 | 201915 | 90255 |
| GC0218A | 457950 | 1201 | 1507 | 18.85 | 232194 | 83974 |
| GC0218C | 219499 | 1335 | 1465 | 19.50 | 160241 | 132841 |
| GC0227A | 791533 | 1288 | 1606 | 16.54 | 316729 | 32384 |
| GC0227C | 747433 | 1307 | 1606 | 16.58 | 307888 | 35734 |
| GC0232A | 490867 | 1141 | 1609 | 16.62 | 161957 | 5723 |
| GC0232C | 440877 | 1191 | 1539 | 16.52 | 167959 | 31122 |
| GC0234A | 526692 | 1246 | 1507 | 16.58 | 236217 | 98425 |
| GC0234C | 236396 | 1295 | 1510 | 16.69 | 108681 | 33573 |
| GC0246A | 293013 | 1293 | 1540 | 16.66 | 127832 | 29529 |
| GC0246C | 204873 | 1202 | 1506 | 16.52 | 85436 | 32676 |
| GC0250A | 315317 | 720 | 1487 | 16.76 | 81721 | 60083 |
| GC0250C | 173461 | 824 | 1486 | 16.74 | 52544 | 39432 |
| GC0256N | 299216 | 1349 | 1761 | 16.69 | 97132 | 7709 |
| GC0256T | 1329691 | 1343 | 1616 | 18.73 | 643922 | 773 |
| GC0271A | 135778 | 1284 | 1465 | 16.88 | 79142 | 58610 |
| GC0271C | 306273 | 1067 | 1494 | 16.71 | 123780 | 66862 |
| GC0272A | 419568 | 1233 | 1505 | 16.71 | 200872 | 87587 |
| GC0272C | 104372 | 1213 | 1496 | 16.51 | 46556 | 21129 |
| GC0280A | 330677 | 1356 | 1614 | 16.59 | 140580 | 3973 |
| GC0280C | 494326 | 1212 | 1602 | 16.62 | 190772 | 5312 |
| GC0281A | 302234 | 1297 | 1596 | 16.59 | 124441 | 18722 |
| GC0281C | 597384 | 1019 | 1509 | 16.58 | 186107 | 18067 |
| GC0287A | 889905 | 552 | 1505 | 16.90 | 130757 | 40032 |
| GC0287C | 101202 | 1204 | 1466 | 16.71 | 45796 | 22132 |
| GC0300A | 251242 | 1231 | 1498 | 16.54 | 118480 | 53313 |
| GC0300C | 295213 | 1216 | 1501 | 16.59 | 133457 | 57775 |
| GC0308N | 1021754 | 1328 | 1628 | 18.98 | 444774 | 132429 |
| GC0308T | 1104957 | 522 | 1444 | 16.83 | 133939 | 5824 |
| GC0312A | 232865 | 1151 | 1599 | 16.71 | 83998 | 1930 |
| GC0312C | 410216 | 1095 | 1600 | 16.58 | 135274 | 4498 |
| GC0323A | 186841 | 1237 | 1466 | 16.74 | 92914 | 53592 |
| GC0323C | 158504 | 1332 | 1464 | 16.89 | 96262 | 75848 |
| GC0332A | 385848 | 697 | 1502 | 16.74 | 96746 | 52273 |
| GC0332C | 77812 | 1154 | 1508 | 16.57 | 32966 | 17816 |
| GC0340A | 271937 | 1313 | 1604 | 16.59 | 115177 | 13909 |
| GC0340C | 156612 | 1191 | 1588 | 16.69 | 64266 | 12811 |
| GC0343A | 142884 | 1154 | 1510 | 16.67 | 57071 | 21960 |
| GC0343C | 156628 | 1182 | 1466 | 16.80 | 73841 | 42639 |
| GC0355A | 264510 | 1191 | 1612 | 16.61 | 93202 | 3056 |
| GC0355C | 315827 | 1283 | 1611 | 16.63 | 125621 | 3978 |
| GC0357N | 1032612 | 1425 | 1615 | 18.90 | 538842 | 716 |
| GC0357T | 1008211 | 1502 | 1617 | 19.02 | 550091 | 1139 |
| GC0366A | 103192 | 1369 | 1519 | 17.19 | 68179 | 66011 |
| GC0366C | 252951 | 1160 | 1505 | 16.69 | 124865 | 67864 |
| GC0366N | 933860 | 1157 | 1614 | 18.77 | 399221 | 38780 |
| GC0366T | 389042 | 1376 | 1612 | 16.83 | 175995 | 1079 |
| GC0373A | 1078028 | 899 | 1485 | 18.85 | 404643 | 151843 |
| GC0373C | 1237822 | 1162 | 1486 | 18.83 | 618492 | 209838 |
| GC0385A | 112653 | 1309 | 1512 | 17.31 | 69763 | 61116 |
| GC0385C | 169461 | 827 | 1476 | 16.95 | 54403 | 47664 |
| GC0431N | 474061 | 1180 | 1605 | 16.55 | 167561 | 296 |
| GC0431T | 528011 | 1141 | 1601 | 16.62 | 175723 | 327 |
| GC0448A | 229988 | 1286 | 1511 | 16.67 | 109037 | 43006 |
| GC0448C | 332533 | 1238 | 1527 | 16.69 | 142265 | 31883 |
| GC0462A | 374130 | 832 | 1503 | 16.75 | 106664 | 36517 |
| GC0462C | 313022 | 1175 | 1503 | 16.68 | 139089 | 64665 |
| GC0466N | 851132 | 1260 | 1536 | 18.94 | 448202 | 179672 |
| GC0466T | 776197 | 1236 | 1537 | 18.91 | 388985 | 114906 |
| GC0470N | 325588 | 1375 | 1525 | 19.29 | 246638 | 215242 |
| GC0470T | 460452 | 1251 | 1529 | 18.92 | 244867 | 123289 |
| GC0487N | 1012991 | 1261 | 1614 | 18.93 | 453683 | 7453 |
| GC0487T | 719237 | 574 | 1339 | 16.71 | 100968 | 538 |
| GC0546N | 433575 | 1246 | 1512 | 19.13 | 281314 | 170919 |
| GC0546T | 316656 | 1295 | 1492 | 18.94 | 198535 | 94567 |
| GC0558N | 76924 | 710 | 1896 | 16.48 | 10077 | 297 |
| GC0558T | 390265 | 1113 | 1608 | 16.51 | 123639 | 400 |

*Summary of sequencing coverage and read quality metrics for each sample included in the study. num_seqs indicates the total number of reads obtained per sample; avg_len indicates the average read length in base pairs (bp); N50 indicates the read length at which 50% of the total bases are contained in reads of that length or longer; AvgQual indicates the average quality score across reads; num_seqs_filtered indicates filtered reads after quality control and num_seqs_assigned indicates number of reads assigned to phylum.*

**Supplementary Table 2. Summary of demographic, clinical, and tissue distribution characteristics in the diagnostic groups of the study.**

| **Main diagnosis and tissue type** | **n** | **Sex** | | **Age** | | **Cohort** | | **H status** |
| --- | --- | --- | --- | --- | --- | --- | --- | --- |
|  |  | **Female** | **Male** | **Mean** | **SD** | **MAGIC** | **BTUCH** |  |
| **GC** | **88** | **33** | **55** | **63,7** | **±13,15** | **29** | **60** | **31** |
| Gastrectomy: Adjacent | 42 | 15 | 27 | 63,6 | ±13,31 | 13 | 30 | 19 |
| Gastrectomy: Tumoral | 42 | 16 | 26 | 64,0 | ±13,61 | 12 | 30 | 12 |
| Biopsy: Antrum | 2 | 1 | 1 | 62,5 | ±9,19 | 2 | 0 | 0 |
| Biopsy: Body | 2 | 1 | 1 | 62,5 | ±9,19 | 2 | 0 | 0 |
| **DY** | **4** | **2** | **2** | **70,5** | **±5,19** | **4** | **0** | **2** |
| Biopsy: Antrum | 2 | 1 | 1 | 70,5 | ±6,36 | 2 | 0 | 1 |
| Biopsy: Body | 2 | 1 | 1 | 70,5 | ±6,36 | 2 | 0 | 1 |
| **IM** | **32** | **18** | **14** | **58,7** | **±11,52** | **32** | **0** | **11** |
| Biopsy: Antrum | 15 | 8 | 7 | 58,3 | ±12,03 | 15 | 0 | 5 |
| Biopsy: Body | 17 | 10 | 7 | 59,0 | ±11,41 | 17 | 0 | 6 |
| **AG** | **8** | **2** | **6** | **64,0** | **±13** | **8** | **0** | **0** |
| Biopsy: Antrum | 4 | 1 | 3 | 64,0 | ±14,04 | 4 | 0 | 0 |
| Biopsy: Body | 4 | 1 | 3 | 64,0 | ±14,04 | 4 | 0 | 0 |
| **SG** | **30** | **20** | **10** | **55,6** | **±9,2** | **30** | **0** | **9** |
| Biopsy: Antrum | 16 | 11 | 5 | 55,3 | ±9,49 | 16 | 0 | 4 |
| Biopsy: Body | 14 | 9 | 5 | 55,9 | ±9,19 | 14 | 0 | 5 |
| **Total general** | **162** | **75** | **87** | **61,4** | **±12,46** | **103** | **60** | **53** |

*Summary of the distribution of tissues and participant characteristics according to the main histopathological diagnosis and tissue type. Variables include total number of tissues (n), sex distribution, mean age ± standard deviation (SD), cohort of origin (MAGIC or BTUCH), and Helicobacter status determined by sequencing analysis (H status). Tissue categories indicate the anatomical origin and specimen type analyzed, including gastrectomy-derived adjacent and tumoral tissues, as well as endoscopic biopsies from the antrum and body. SG = Superficial Gastritis, AG = Atrophic Gastritis, IM = Intestinal Metaplasia, DY = Dysplasia, GC = Gastric Cancer. GC tissues include both early and advanced cases*

**Supplementary Table 3. Complete tissue-level metadata of the study.**

| **n** | **tissue_id** | **individual_id** | **cohort** | **sex** | **age** | **tissue_type** | **anatomical_site** | **main_diagnosis** | **lauren_classification** | **gc_stage** | **filter_pass** | **HP_status_by_seq** |
| --- | --- | --- | --- | --- | --- | --- | --- | --- | --- | --- | --- | --- |
| 1 | 51519N | 51519 | BTUCH | Female | 56 | Gastrectomy | Adjacent | GC | Intestinal | Advanced | Yes | Yes |
| 2 | 51519T | 51519 | BTUCH | Female | 56 | Gastrectomy | Tumoral | GC | Intestinal | Advanced | Yes | Yes |
| 3 | 51631N | 51631 | BTUCH | Female | 63 | Gastrectomy | Adjacent | GC | Diffuse | Advanced | Yes | No |
| 4 | 51631T | 51631 | BTUCH | Female | 63 | Gastrectomy | Tumoral | GC | Diffuse | Advanced | Yes | No |
| 5 | 51637N | 51637 | BTUCH | Male | 67 | Gastrectomy | Adjacent | GC | Diffuse | Early | Yes | Yes |
| 6 | 51637T | 51637 | BTUCH | Male | 67 | Gastrectomy | Tumoral | GC | Diffuse | Early | Yes | No |
| 7 | 51638T | 51638 | BTUCH | Female | 58 | Gastrectomy | Tumoral | GC | Intestinal | Advanced | Yes | Yes |
| 8 | 51646N | 51646 | BTUCH | Male | 76 | Gastrectomy | Adjacent | GC | Intestinal | Advanced | Yes | No |
| 9 | 51646T | 51646 | BTUCH | Male | 76 | Gastrectomy | Tumoral | GC | Intestinal | Advanced | Yes | No |
| 10 | 51655N | 51655 | BTUCH | Female | 46 | Gastrectomy | Adjacent | GC | Diffuse | Advanced | Yes | Yes |
| 11 | 51655T | 51655 | BTUCH | Female | 46 | Gastrectomy | Tumoral | GC | Diffuse | Advanced | Yes | Yes |
| 12 | 51659T | 51659 | BTUCH | Male | 62 | Gastrectomy | Tumoral | GC | Mixed | Advanced | Yes | Yes |
| 13 | 51660N | 51660 | BTUCH | Female | 25 | Gastrectomy | Adjacent | GC | Diffuse | Advanced | Yes | Yes |
| 14 | 51660T | 51660 | BTUCH | Female | 25 | Gastrectomy | Tumoral | GC | Diffuse | Advanced | Yes | No |
| 15 | 51661N | 51661 | BTUCH | Male | 60 | Gastrectomy | Adjacent | GC | Intestinal | Advanced | Yes | No |
| 16 | 51661T | 51661 | BTUCH | Male | 60 | Gastrectomy | Tumoral | GC | Intestinal | Advanced | Yes | No |
| 17 | 51712N | 51712 | BTUCH | Female | 72 | Gastrectomy | Adjacent | GC | Diffuse | Advanced | Yes | No |
| 18 | 51712T | 51712 | BTUCH | Female | 72 | Gastrectomy | Tumoral | GC | Diffuse | Advanced | Yes | No |
| 19 | 5171N | 5171 | BTUCH | Male | 39 | Gastrectomy | Adjacent | GC | Diffuse | Advanced | Yes | No |
| 20 | 5171T | 5171 | BTUCH | Male | 39 | Gastrectomy | Tumoral | GC | Diffuse | Advanced | Yes | No |
| 21 | 5172N | 5172 | BTUCH | Male | 79 | Gastrectomy | Adjacent | GC | Diffuse | Advanced | Yes | Yes |
| 22 | 5172T | 5172 | BTUCH | Male | 79 | Gastrectomy | Tumoral | GC | Diffuse | Advanced | Yes | Yes |
| 23 | 5173N | 5173 | BTUCH | Male | 51 | Gastrectomy | Adjacent | GC | Diffuse | Advanced | Yes | No |
| 24 | 5173T | 5173 | BTUCH | Male | 51 | Gastrectomy | Tumoral | GC | Diffuse | Advanced | Yes | No |
| 25 | 5174T | 5174 | BTUCH | Female | 62 | Gastrectomy | Tumoral | GC | Intestinal | Advanced | Yes | No |
| 26 | 51768N | 51768 | BTUCH | Female | 33 | Gastrectomy | Adjacent | GC | Intestinal | Advanced | Yes | No |
| 27 | 51768T | 51768 | BTUCH | Female | 33 | Gastrectomy | Tumoral | GC | Intestinal | Advanced | Yes | No |
| 28 | 51771T | 51771 | BTUCH | Female | 82 | Gastrectomy | Tumoral | GC | Diffuse | Advanced | Yes | No |
| 29 | 5177N | 5177 | BTUCH | Male | 65 | Gastrectomy | Adjacent | GC | Diffuse | Early | Yes | Yes |
| 30 | 5177T | 5177 | BTUCH | Male | 65 | Gastrectomy | Tumoral | GC | Diffuse | Early | Yes | Yes |
| 31 | 51873N | 51873 | BTUCH | Male | 69 | Gastrectomy | Adjacent | GC | Diffuse | Advanced | Yes | Yes |
| 32 | 51873T | 51873 | BTUCH | Male | 69 | Gastrectomy | Tumoral | GC | Diffuse | Advanced | Yes | Yes |
| 33 | 51874N | 51874 | BTUCH | Male | 58 | Gastrectomy | Adjacent | GC | Diffuse | Advanced | Yes | Yes |
| 34 | 51874T | 51874 | BTUCH | Male | 58 | Gastrectomy | Tumoral | GC | Diffuse | Advanced | Yes | Yes |
| 35 | 51989N | 51989 | BTUCH | Male | 69 | Gastrectomy | Adjacent | GC | Mixed | Advanced | Yes | Yes |
| 36 | 51991N | 51991 | BTUCH | Male | 56 | Gastrectomy | Adjacent | GC | Diffuse | Early | Yes | Yes |
| 37 | 51991T | 51991 | BTUCH | Male | 56 | Gastrectomy | Tumoral | GC | Diffuse | Early | Yes | No |
| 38 | 51992N | 51992 | BTUCH | Female | 84 | Gastrectomy | Adjacent | GC | Diffuse | Advanced | Yes | Yes |
| 39 | 520103N | 520103 | BTUCH | Female | 73 | Gastrectomy | Adjacent | GC | Diffuse | Early | Yes | Yes |
| 40 | 520103T | 520103 | BTUCH | Female | 73 | Gastrectomy | Tumoral | GC | Diffuse | Early | Yes | No |
| 41 | 520104N | 520104 | BTUCH | Male | 63 | Gastrectomy | Adjacent | GC | Intestinal | Advanced | Yes | Yes |
| 42 | 520104T | 520104 | BTUCH | Male | 63 | Gastrectomy | Tumoral | GC | Intestinal | Advanced | Yes | Yes |
| 43 | 52110N | 52110 | BTUCH | Male | 70 | Gastrectomy | Adjacent | GC | Intestinal | Advanced | Yes | Yes |
| 44 | 52110T | 52110 | BTUCH | Male | 70 | Gastrectomy | Tumoral | GC | Intestinal | Advanced | Yes | No |
| 45 | 5213N | 5213 | BTUCH | Female | 41 | Gastrectomy | Adjacent | GC | Intestinal | Advanced | Yes | No |
| 46 | 5213T | 5213 | BTUCH | Female | 41 | Gastrectomy | Tumoral | GC | Intestinal | Advanced | Yes | No |
| 47 | 5214N | 5214 | BTUCH | Female | 70 | Gastrectomy | Adjacent | GC | Diffuse | Early | Yes | No |
| 48 | 5214T | 5214 | BTUCH | Female | 70 | Gastrectomy | Tumoral | GC | Diffuse | Early | Yes | No |
| 49 | 5219N | 5219 | BTUCH | Male | 70 | Gastrectomy | Adjacent | GC | Diffuse | Advanced | Yes | No |
| 50 | 5219T | 5219 | BTUCH | Male | 70 | Gastrectomy | Tumoral | GC | Diffuse | Advanced | Yes | Yes |
| 51 | 5220006N | 5220006 | BTUCH | Male | 82 | Gastrectomy | Adjacent | GC | Diffuse | Advanced | Yes | Yes |
| 52 | 5220006T | 5220006 | BTUCH | Male | 82 | Gastrectomy | Tumoral | GC | Diffuse | Advanced | Yes | Yes |
| 53 | GC0026A | GC0026 | MAGIC | Female | 41 | Biopsy | Antrum | SG | NA | NA | Yes | No |
| 54 | GC0029A | GC0029 | MAGIC | Male | 78 | Biopsy | Antrum | AG | NA | NA | Yes | No |
| 55 | GC0029C | GC0029 | MAGIC | Male | 78 | Biopsy | Body | AG | NA | NA | Yes | No |
| 56 | GC0041A | GC0041 | MAGIC | Male | 57 | Biopsy | Antrum | SG | NA | NA | Yes | No |
| 57 | GC0041C | GC0041 | MAGIC | Male | 57 | Biopsy | Body | SG | NA | NA | Yes | No |
| 58 | GC0046A | GC0046 | MAGIC | Female | 66 | Biopsy | Antrum | DY | NA | NA | Yes | Yes |
| 59 | GC0046C | GC0046 | MAGIC | Female | 66 | Biopsy | Body | DY | NA | NA | Yes | Yes |
| 60 | GC0055A | GC0055 | MAGIC | Female | 40 | Biopsy | Antrum | IM | NA | NA | Yes | Yes |
| 61 | GC0055C | GC0055 | MAGIC | Female | 40 | Biopsy | Body | IM | NA | NA | Yes | Yes |
| 62 | GC0095A | GC0095 | MAGIC | Female | 79 | Biopsy | Antrum | IM | NA | NA | Yes | No |
| 63 | GC0095C | GC0095 | MAGIC | Female | 79 | Biopsy | Body | IM | NA | NA | Yes | No |
| 64 | GC0116A | GC0116 | MAGIC | Female | 70 | Biopsy | Antrum | SG | NA | NA | Yes | Yes |
| 65 | GC0116C | GC0116 | MAGIC | Female | 70 | Biopsy | Body | SG | NA | NA | Yes | Yes |
| 66 | GC0140A | GC0140 | MAGIC | Male | 74 | Biopsy | Antrum | AG | NA | NA | Yes | No |
| 67 | GC0140C | GC0140 | MAGIC | Male | 74 | Biopsy | Body | AG | NA | NA | Yes | No |
| 68 | GC0156A | GC0156 | MAGIC | Female | 39 | Biopsy | Antrum | SG | NA | NA | Yes | Yes |
| 69 | GC0156C | GC0156 | MAGIC | Female | 39 | Biopsy | Body | SG | NA | NA | Yes | No |
| 70 | GC0157A | GC0157 | MAGIC | Male | 76 | Biopsy | Antrum | IM | NA | NA | Yes | No |
| 71 | GC0157C | GC0157 | MAGIC | Male | 76 | Biopsy | Body | IM | NA | NA | Yes | No |
| 72 | GC0159A | GC0159 | MAGIC | Female | 52 | Biopsy | Antrum | IM | NA | NA | Yes | Yes |
| 73 | GC0159C | GC0159 | MAGIC | Female | 52 | Biopsy | Body | IM | NA | NA | Yes | Yes |
| 74 | GC0163A | GC0163 | MAGIC | Male | 51 | Biopsy | Antrum | SG | NA | NA | Yes | Yes |
| 75 | GC0163C | GC0163 | MAGIC | Male | 51 | Biopsy | Body | SG | NA | NA | Yes | Yes |
| 76 | GC0165N | GC0165 | MAGIC | Male | 71 | Gastrectomy | Adjacent | GC | Mixed | Advanced | Yes | Yes |
| 77 | GC0165T | GC0165 | MAGIC | Male | 71 | Gastrectomy | Tumoral | GC | Mixed | Advanced | Yes | Yes |
| 78 | GC0166A | GC0166 | MAGIC | Female | 63 | Biopsy | Antrum | SG | NA | NA | Yes | No |
| 79 | GC0166C | GC0166 | MAGIC | Female | 63 | Biopsy | Body | SG | NA | NA | Yes | No |
| 80 | GC0167A | GC0167 | MAGIC | Female | 62 | Biopsy | Antrum | SG | NA | NA | Yes | No |
| 81 | GC0173A | GC0173 | MAGIC | Male | 61 | Biopsy | Antrum | IM | NA | NA | Yes | No |
| 82 | GC0173C | GC0173 | MAGIC | Male | 61 | Biopsy | Body | IM | NA | NA | Yes | No |
| 83 | GC0194A | GC0194 | MAGIC | Female | 45 | Biopsy | Antrum | IM | NA | NA | Yes | Yes |
| 84 | GC0194C | GC0194 | MAGIC | Female | 45 | Biopsy | Body | IM | NA | NA | Yes | Yes |
| 85 | GC0206A | GC0206 | MAGIC | Female | 54 | Biopsy | Antrum | AG | NA | NA | Yes | No |
| 86 | GC0206C | GC0206 | MAGIC | Female | 54 | Biopsy | Body | AG | NA | NA | Yes | No |
| 87 | GC0218A | GC0218 | MAGIC | Female | 47 | Biopsy | Antrum | SG | NA | NA | Yes | Yes |
| 88 | GC0218C | GC0218 | MAGIC | Female | 47 | Biopsy | Body | SG | NA | NA | Yes | Yes |
| 89 | GC0227A | GC0227 | MAGIC | Female | 52 | Biopsy | Antrum | SG | NA | NA | Yes | No |
| 90 | GC0227C | GC0227 | MAGIC | Female | 52 | Biopsy | Body | SG | NA | NA | Yes | No |
| 91 | GC0232A | GC0232 | MAGIC | Female | 46 | Biopsy | Antrum | IM | NA | NA | Yes | No |
| 92 | GC0232C | GC0232 | MAGIC | Female | 46 | Biopsy | Body | IM | NA | NA | Yes | No |
| 93 | GC0234A | GC0234 | MAGIC | Female | 68 | Biopsy | Antrum | IM | NA | NA | Yes | No |
| 94 | GC0234C | GC0234 | MAGIC | Female | 68 | Biopsy | Body | IM | NA | NA | Yes | No |
| 95 | GC0246A | GC0246 | MAGIC | Male | 44 | Biopsy | Antrum | SG | NA | NA | Yes | No |
| 96 | GC0246C | GC0246 | MAGIC | Male | 44 | Biopsy | Body | SG | NA | NA | Yes | No |
| 97 | GC0250A | GC0250 | MAGIC | Female | 72 | Biopsy | Antrum | SG | NA | NA | Yes | No |
| 98 | GC0250C | GC0250 | MAGIC | Female | 72 | Biopsy | Body | SG | NA | NA | Yes | No |
| 99 | GC0256N | GC0256 | MAGIC | Male | 67 | Gastrectomy | Adjacent | GC | Diffuse | Advanced | Yes | No |
| 100 | GC0271A | GC0271 | MAGIC | Male | 55 | Biopsy | Antrum | IM | NA | NA | Yes | Yes |
| 101 | GC0271C | GC0271 | MAGIC | Male | 55 | Biopsy | Body | IM | NA | NA | Yes | Yes |
| 102 | GC0272A | GC0272 | MAGIC | Female | 67 | Biopsy | Antrum | IM | NA | NA | Yes | No |
| 103 | GC0272C | GC0272 | MAGIC | Female | 67 | Biopsy | Body | IM | NA | NA | Yes | No |
| 104 | GC0280C | GC0280 | MAGIC | Male | 56 | Biopsy | Body | SG | NA | NA | Yes | No |
| 105 | GC0281A | GC0281 | MAGIC | Female | 55 | Biopsy | Antrum | SG | NA | NA | Yes | No |
| 106 | GC0281C | GC0281 | MAGIC | Female | 55 | Biopsy | Body | SG | NA | NA | Yes | No |
| 107 | GC0287A | GC0287 | MAGIC | Female | 56 | Biopsy | Antrum | IM | NA | NA | Yes | No |
| 108 | GC0287C | GC0287 | MAGIC | Female | 56 | Biopsy | Body | IM | NA | NA | Yes | Yes |
| 109 | GC0300A | GC0300 | MAGIC | Male | 61 | Biopsy | Antrum | IM | NA | NA | Yes | No |
| 110 | GC0300C | GC0300 | MAGIC | Male | 61 | Biopsy | Body | IM | NA | NA | Yes | No |
| 111 | GC0308N | GC0308 | MAGIC | Male | 68 | Gastrectomy | Adjacent | GC | Intestinal | Advanced | Yes | Yes |
| 112 | GC0308T | GC0308 | MAGIC | Male | 68 | Gastrectomy | Tumoral | GC | Intestinal | Advanced | Yes | No |
| 113 | GC0323A | GC0323 | MAGIC | Male | 47 | Biopsy | Antrum | IM | NA | NA | Yes | Yes |
| 114 | GC0323C | GC0323 | MAGIC | Male | 47 | Biopsy | Body | IM | NA | NA | Yes | Yes |
| 115 | GC0332A | GC0332 | MAGIC | Male | 72 | Biopsy | Antrum | IM | NA | NA | Yes | No |
| 116 | GC0332C | GC0332 | MAGIC | Male | 72 | Biopsy | Body | IM | NA | NA | Yes | No |
| 117 | GC0340A | GC0340 | MAGIC | Female | 55 | Biopsy | Antrum | SG | NA | NA | Yes | No |
| 118 | GC0340C | GC0340 | MAGIC | Female | 55 | Biopsy | Body | SG | NA | NA | Yes | No |
| 119 | GC0343A | GC0343 | MAGIC | Male | 61 | Biopsy | Antrum | SG | NA | NA | Yes | No |
| 120 | GC0343C | GC0343 | MAGIC | Male | 61 | Biopsy | Body | SG | NA | NA | Yes | Yes |
| 121 | GC0366A | GC0366_2 | MAGIC | Male | 69 | Biopsy | Antrum | GC | Intestinal | Advanced | Yes | No |
| 122 | GC0366C | GC0366_2 | MAGIC | Male | 69 | Biopsy | Body | GC | Intestinal | Advanced | Yes | No |
| 123 | GC0366N | GC0366 | MAGIC | Male | 69 | Gastrectomy | Adjacent | GC | Intestinal | Advanced | Yes | Yes |
| 124 | GC0373A | GC0373 | MAGIC | Female | 56 | Biopsy | Antrum | GC | Diffuse | Advanced | Yes | No |
| 125 | GC0373C | GC0373 | MAGIC | Female | 56 | Biopsy | Body | GC | Diffuse | Advanced | Yes | No |
| 126 | GC0385A | GC0385 | MAGIC | Female | 60 | Biopsy | Antrum | SG | NA | NA | Yes | No |
| 127 | GC0385C | GC0385 | MAGIC | Female | 60 | Biopsy | Body | SG | NA | NA | Yes | Yes |
| 128 | GC0448A | GC0448 | MAGIC | Male | 75 | Biopsy | Antrum | DY | NA | NA | Yes | No |
| 129 | GC0448C | GC0448 | MAGIC | Male | 75 | Biopsy | Body | DY | NA | NA | Yes | No |
| 130 | GC0462A | GC0462 | MAGIC | Male | 50 | Biopsy | Antrum | AG | NA | NA | Yes | No |
| 131 | GC0462C | GC0462 | MAGIC | Male | 50 | Biopsy | Body | AG | NA | NA | Yes | No |
| 132 | GC0466N | GC0466 | MAGIC | Male | 88 | Gastrectomy | Adjacent | GC | Intestinal | Advanced | Yes | No |
| 133 | GC0466T | GC0466 | MAGIC | Male | 88 | Gastrectomy | Tumoral | GC | Intestinal | Advanced | Yes | No |
| 134 | GC0470N | GC0470 | MAGIC | Female | 78 | Gastrectomy | Adjacent | GC | Mixed | Advanced | Yes | No |
| 135 | GC0470T | GC0470 | MAGIC | Female | 78 | Gastrectomy | Tumoral | GC | Mixed | Advanced | Yes | No |
| 136 | GC0487N | GC0487 | MAGIC | Male | 78 | Gastrectomy | Adjacent | GC | Intestinal | Advanced | Yes | Yes |
| 137 | GC0546N | GC0546 | MAGIC | Male | 55 | Gastrectomy | Adjacent | GC | Diffuse | Advanced | Yes | No |
| 138 | GC0546T | GC0546 | MAGIC | Male | 55 | Gastrectomy | Tumoral | GC | Diffuse | Advanced | Yes | No |
| 139 | 51525N | 51525 | BTUCH | Female | 72 | Gastrectomy | Adjacent | GC | Diffuse | Early | No | NA |
| 140 | 51525T | 51525 | BTUCH | Female | 72 | Gastrectomy | Tumoral | GC | Diffuse | Early | No | NA |
| 141 | 51638N | 51638 | BTUCH | Female | 58 | Gastrectomy | Adjacent | GC | Intestinal | Advanced | No | NA |
| 142 | 51659N | 51659 | BTUCH | Male | 62 | Gastrectomy | Adjacent | GC | Mixed | Advanced | No | NA |
| 143 | 5174N | 5174 | BTUCH | Female | 62 | Gastrectomy | Adjacent | GC | Intestinal | Advanced | No | NA |
| 144 | 51989T | 51989 | BTUCH | Male | 69 | Gastrectomy | Tumoral | GC | Mixed | Advanced | No | NA |
| 145 | 51992T | 51992 | BTUCH | Female | 84 | Gastrectomy | Tumoral | GC | Diffuse | Advanced | No | NA |
| 146 | 51993N | 51993 | BTUCH | Male | 65 | Gastrectomy | Adjacent | GC | Intestinal | Early | No | NA |
| 147 | GC0001N | GC0001 | MAGIC | Male | 62 | Gastrectomy | Adjacent | GC | Intestinal | Early | No | NA |
| 148 | GC0001T | GC0001 | MAGIC | Male | 62 | Gastrectomy | Tumoral | GC | Intestinal | Early | No | NA |
| 149 | GC0256T | GC0256 | MAGIC | Male | 67 | Gastrectomy | Tumoral | GC | Diffuse | Advanced | No | NA |
| 150 | GC0280A | GC0280 | MAGIC | Male | 56 | Biopsy | Antrum | SG | NA | NA | No | NA |
| 151 | GC0312A | GC0312 | MAGIC | Female | 64 | Biopsy | Body | IM | NA | NA | No | NA |
| 152 | GC0312C | GC0312 | MAGIC | Female | 64 | Biopsy | Body | IM | NA | NA | No | NA |
| 153 | GC0355A | GC0355 | MAGIC | Male | 50 | Biopsy | Antrum | IM | NA | NA | No | NA |
| 154 | GC0355C | GC0355 | MAGIC | Male | 50 | Biopsy | Body | IM | NA | NA | No | NA |
| 155 | GC0357N | GC0357 | MAGIC | Male | 59 | Gastrectomy | Adjacent | GC | Intestinal | Early | No | NA |
| 156 | GC0357T | GC0357 | MAGIC | Male | 59 | Gastrectomy | Tumoral | GC | Intestinal | Early | No | NA |
| 157 | GC0366T | GC0366 | MAGIC | Male | 69 | Gastrectomy | Tumoral | GC | Intestinal | Advanced | No | NA |
| 158 | GC0431N | GC0431 | MAGIC | Male | 71 | Gastrectomy | Adjacent | GC | Intestinal | Early | No | NA |
| 159 | GC0431T | GC0431 | MAGIC | Male | 71 | Gastrectomy | Tumoral | GC | Intestinal | Early | No | NA |
| 160 | GC0487T | GC0487 | MAGIC | Male | 78 | Gastrectomy | Tumoral | GC | Intestinal | Advanced | No | NA |
| 161 | GC0558N | GC0558 | MAGIC | Female | 49 | Gastrectomy | Adjacent | GC | NA | Early | No | NA |
| 162 | GC0558T | GC0558 | MAGIC | Female | 49 | Gastrectomy | Tumoral | GC | NA | Early | No | NA |

*Each row corresponds to an individual tissue sample analyzed in this study. The columns indicate: n, sample number identifier; tissue_id, unique tissue sample code; individual_id, participant identifier; cohort, study cohort of origin (MAGIC or BTUCH); sex, biological sex of the participant; age, age at sample collection; tissue_type, type of specimen collected (biopsy or gastrectomy); anatomical_site, anatomical location of the tissue (antrum, body, adjacent, or tumoral); main_diagnosis, primary histopathological diagnosis; lauren_classification, histological subtype according to Lauren classification (only for GC); gc_stage, GC stage classification (early or advanced, only for GC); filter_pass, indicates whether the sample passed quality-control and technical filtering criteria for inclusion in downstream analyses; and HP_status_by_seq, Helicobacter detection status determined by sequencing analysis. SG = Superficial Gastritis, AG = Atrophic Gastritis, IM = Intestinal Metaplasia, DY = Dysplasia, GC = Gastric Cancer.*

**Supplementary Table 4.** **DeSeq2 analysis for differentially abundant genera through pairwise comparisons in biopsy tissues from the MAGIC cohort.**

| ***Genus*** | **log₂FC** | | | | | | | | | |
| --- | --- | --- | --- | --- | --- | --- | --- | --- | --- | --- |
|  | **AG vs SG** | **IM vs SG** | **DY vs SG** | **GC vs SG** | **IM vs AG** | **DY vs AG** | **GC vs AG** | **DY vs IM** | **GC vs IM** | **GC vs DY** |
| *Acidovorax* | -29.92 | -7.55 | -23.68 | -25.15 | 22.37 | 0.00 | 0.00 | -16.13 | -17.61 | 0.00 |
| *Agathobacter* | 30.00 | -8.68 | -2.04 | -2.03 | -38.68 | -32.04 | -32.03 | 6.63 | 6.64 | 0.00 |
| *Alloprevotella* | -0.14 | -0.23 | -27.55 | 3.18 | -0.09 | -27.41 | 3.33 | -27.32 | 3.41 | 30.74 |
| *Alloscardovia* | -30.00 | -3.09 | -21.94 | 2.77 | 26.91 | 0.00 | 32.77 | -18.85 | 5.86 | 24.71 |
| *Anaerococcus* | 4.29 | -4.21 | -24.29 | -23.08 | -8.49 | -28.58 | -27.36 | -20.09 | -18.87 | 0.00 |
| *Bacteroides* | 24.37 | 0.00 | 0.00 | 24.87 | -35.69 | -27.16 | 0.50 | 0.00 | 36.20 | 27.67 |
| *Blautia* | 29.00 | 0.00 | 0.00 | 0.00 | -33.05 | -30.90 | -30.83 | 0.00 | 0.00 | 0.00 |
| *Bradyrhizobium* | -4.09 | -1.99 | -26.50 | -25.91 | 2.10 | -22.41 | -21.82 | -24.51 | -23.92 | 0.00 |
| *Bulleidia* | 20.20 | 25.00 | -6.37 | 25.49 | 4.81 | -26.57 | 5.30 | -31.38 | 0.49 | 31.87 |
| *Catonella* | 02.02 | -1.25 | -24.86 | 2.62 | -3.27 | -26.88 | 0.60 | -23.62 | 3.86 | 27.48 |
| *Centipeda / Selenomonas (mixed species)* | 0.00 | 23.54 | 0.00 | 25.68 | 30.05 | 0.00 | 32.19 | -27.74 | 2.14 | 29.88 |
| *Dialister* | 0.03 | -1.57 | -26.82 | 1.72 | -1.60 | -26.85 | 1.69 | -25.25 | 3.29 | 28.54 |
| *Eikenella* | -1.37 | -2.21 | -25.60 | -0.95 | -0.84 | -24.23 | 0.43 | -23.39 | 1.27 | 24.65 |
| *Escherichia* | 25.49 | -3.85 | 0.00 | 0.00 | -29.34 | -27.18 | -27.18 | 2.16 | 2.16 | 0.00 |
| *Escherichia / Shigella (mixed species)* | 6.42 | -22.17 | -19.47 | -19.16 | -28.59 | -25.90 | -25.58 | 2.69 | 03.01 | 0.00 |
| *Filifactor* | -24.94 | 2.38 | 2.12 | 2.94 | 27.31 | 27.05 | 27.87 | -0.26 | 0.56 | 0.82 |
| *Fusobacterium* | 0.12 | -0.79 | -2.53 | 4.96 | -0.92 | -2.65 | 4.84 | -1.73 | 5.75 | 7.48 |
| *Gemmiger* | 25.35 | -4.18 | 0.00 | 0.00 | -29.53 | -27.44 | -27.38 | 02.09 | 2.14 | 0.00 |
| *Gemmiger / Subdoligranulum (mixed species)* | 8.53 | -21.83 | -15.07 | -18.28 | -30.36 | -23.60 | -26.81 | 6.76 | 3.55 | 0.00 |
| *Helicobacter* | -11.56 | 0.01 | -0.81 | -6.51 | 11.57 | 10.75 | 05.05 | -0.82 | -6.53 | -5.70 |
| *Hoylesella* | -0.90 | -1.91 | -28.00 | 0.67 | -1.01 | -27.10 | 1.57 | -26.10 | 2.58 | 28.67 |
| *Lachnoanaerobaculum* | 0.92 | -0.57 | -6.33 | 0.94 | -1.50 | -7.25 | 0.02 | -5.76 | 1.52 | 7.28 |
| *Lactobacillus* | -3.65 | -5.43 | -6.82 | 2.64 | -1.79 | -3.17 | 6.28 | -1.38 | 08.07 | 9.45 |
| *Leyella* | 6.35 | -22.13 | -18.24 | -20.14 | -28.47 | -24.59 | -26.49 | 0.00 | 0.00 | 0.00 |
| *Ligilactobacillus* | -3.04 | -0.78 | -23.81 | -24.25 | 2.25 | -20.77 | -21.22 | -23.03 | -23.47 | 0.00 |
| *Limosilactobacillus* | -1.61 | -2.59 | -1.05 | 4.96 | -0.98 | 0.55 | 6.56 | 1.54 | 7.55 | 06.01 |
| *Mammaliicoccus / Staphylococcus (mixed species)* | -10.35 | -9.48 | -9.55 | -6.73 | 0.87 | 0.80 | 3.62 | 0.00 | 0.00 | 0.00 |
| *Mediterraneibacter* | 20.38 | 23.14 | 0.00 | 0.00 | 2.76 | -25.28 | -25.09 | -28.04 | -27.85 | 0.00 |
| *Moraxella* | -29.94 | -25.37 | -23.67 | 3.18 | 4.56 | 0.00 | 33.12 | 1.71 | 28.55 | 26.85 |
| *Neisseria* | -0.19 | -1.72 | -4.21 | 0.34 | -1.53 | -4.02 | 0.53 | -2.49 | 02.06 | 4.55 |
| *Olsenella* | -22.79 | -1.35 | 2.90 | 2.76 | 21.43 | 25.69 | 25.54 | 4.25 | 4.11 | -0.14 |
| *Parvimonas / Peptostreptococcus (mixed species)* | 0.48 | -2.56 | -23.80 | 1.16 | -3.04 | -24.28 | 0.68 | -21.24 | 3.73 | 24.96 |
| *Peptoanaerobacter* | -21.75 | -29.96 | -19.39 | 5.58 | -8.22 | 2.35 | 27.32 | 10.57 | 35.54 | 24.97 |
| *Peptostreptococcus* | -0.65 | -1.94 | -7.39 | 1.72 | -1.29 | -6.74 | 2.38 | -5.46 | 3.66 | 9.12 |
| *Phascolarctobacterium* | 28.10 | 0.00 | 0.00 | 0.00 | -32.82 | -29.26 | -29.32 | 0.00 | 0.00 | 0.00 |
| *Prevotella* | 0.75 | 0.02 | -1.60 | 1.78 | -0.73 | -2.35 | 01.03 | -1.62 | 1.76 | 3.38 |
| *Prevotella / Segatella (mixed species)* | 0.63 | -0.47 | -23.32 | 0.75 | -1.10 | -23.95 | 0.12 | -22.85 | 1.22 | 24.07 |
| *Prevotellamassilia* | 24.76 | 0.00 | 0.00 | 0.00 | -29.76 | -27.57 | -27.37 | 0.00 | 0.00 | 0.00 |
| *Pseudomonas* | -6.51 | -2.88 | -8.03 | -4.82 | 3.63 | -1.53 | 1.69 | -5.16 | -1.94 | 3.22 |
| *Pseudostreptobacillus* | -20.72 | 2.87 | -20.02 | 7.68 | 23.60 | 0.00 | 28.41 | -22.89 | 4.81 | 27.70 |
| *Roseburia* | 28.43 | 0.00 | 0.00 | 0.00 | -39.24 | -31.08 | -31.04 | 0.00 | 0.00 | 0.00 |
| *Ruminococcoides* | 25.88 | 20.04 | 0.00 | 0.00 | -5.84 | -32.48 | -32.52 | -26.63 | -26.67 | 0.00 |
| *Segatella* | 1.57 | -0.33 | -4.14 | 0.65 | -1.90 | -5.72 | -0.93 | -3.81 | 0.97 | 4.79 |
| *Selenomonas* | 5.56 | 1.47 | -25.15 | 4.96 | -4.08 | -30.71 | -0.60 | -26.62 | 3.49 | 30.11 |
| *Shuttleworthella* | -26.09 | -29.18 | -24.78 | 1.64 | 0.00 | 0.00 | 27.73 | 0.00 | 30.82 | 26.42 |
| *Solobacterium* | -0.34 | -1.48 | -27.40 | 0.89 | -1.14 | -27.07 | 1.23 | -25.92 | 2.37 | 28.29 |
| *Tannerella* | -29.98 | 0.42 | -23.83 | 2.21 | 30.40 | 0.00 | 32.19 | -24.25 | 1.79 | 26.05 |
| *species:[Eubacterium] nodatum* | -25.45 | 1.60 | 2.59 | -21.92 | 27.05 | 28.04 | 0.00 | 0.99 | -23.51 | -24.51 |
| *species:[Eubacterium] sulci* | -0.06 | 0.38 | -24.98 | 1.71 | 0.45 | -24.91 | 1.77 | -25.36 | 1.32 | 26.68 |

*The table shows the log₂ fold change (log₂FC) values obtained from DESeq2 differential abundance analysis for genera identified in pairwise comparisons among diagnosis groups from biopsy tissues in the MAGIC cohort. Positive log₂FC values indicate relative enrichment of a genera in the first group of the comparison, whereas negative values indicate relative depletion. A total of 73 tissues passing quality-control filters were included in the analysis. SG = Superficial Gastritis, AG = Atrophic Gastritis, IM = Intestinal Metaplasia, DY = Dysplasia, GC = Gastric Cancer.*

**Supplementary Table 5. DeSeq2 analysis for differentially abundant genera through pairwise comparisons in biopsy tissues from the MAGIC cohort.**

| ***Genus*** | **Adjusted p-values** | | | | | | | | | |
| --- | --- | --- | --- | --- | --- | --- | --- | --- | --- | --- |
|  | **AG vs SG** | **IM vs SG** | **DY vs SG** | **GC vs SG** | **IM vs AG** | **DY vs AG** | **GC vs AG** | **DY vs IM** | **GC vs IM** | **GC vs DY** |
| *Acidovorax* | 3.691 x E-08 | 2.272 x E-01 | 2.418 x E-03 | 3.980 x E-03 | 5.986 x E-05 | 1.000 x E+00 | 1.000 x E+00 | 7.826 x E-02 | 8.106 x E-02 | 1.000 x E+00 |
| *Agathobacter* | 3.487 x E-08 | 9.993 x E-02 | 1.000 x E+00 | 1.000 x E+00 | 2.555 x E-13 | 2.106 x E-04 | 3.977 x E-04 | 8.199 x E-01 | 1.000 x E+00 | 1.000 x E+00 |
| *Alloprevotella* | 1.000 x E+00 | 9.806 x E-01 | 1.939 x E-20 | 1.000 x E+00 | 1.000 x E+00 | 7.724 x E-16 | 8.171 x E-01 | 7.091 x E-20 | 1.000 x E+00 | 7.029 x E-15 |
| *Alloscardovia* | 3.328 x E-12 | 7.126 x E-01 | 3.811 x E-04 | 1.000 x E+00 | 5.442 x E-10 | 1.000 x E+00 | 3.934 x E-06 | 2.759 x E-03 | 1.000 x E+00 | 3.191 x E-03 |
| *Anaerococcus* | 7.376 x E-01 | 7.126 x E-01 | 2.018 x E-03 | 9.877 x E-03 | 6.085 x E-01 | 9.535 x E-04 | 1.947 x E-03 | 1.428 x E-02 | 4.858 x E-02 | 1.000 x E+00 |
| *Bacteroides* | 5.718 x E-06 | 1.000 x E+00 | 1.000 x E+00 | 3.980 x E-03 | 1.692 x E-11 | 1.650 x E-03 | 1.000 x E+00 | 1.000 x E+00 | 5.468 x E-06 | 8.514 x E-03 |
| *Blautia* | 6.741 x E-08 | 1.000 x E+00 | 1.000 x E+00 | 1.000 x E+00 | 5.539 x E-10 | 3.539 x E-04 | 5.832 x E-04 | 1.000 x E+00 | 1.000 x E+00 | 1.000 x E+00 |
| *Bradyrhizobium* | 7.376 x E-01 | 7.471 x E-01 | 4.609 x E-06 | 1.219 x E-04 | 1.000 x E+00 | 9.535 x E-04 | 1.947 x E-03 | 2.637 x E-05 | 1.813 x E-04 | 1.000 x E+00 |
| *Bulleidia* | 2.474 x E-04 | 9.122 x E-13 | 9.385 x E-01 | 3.980 x E-03 | 1.000 x E+00 | 2.060 x E-03 | 1.000 x E+00 | 2.522 x E-05 | 1.000 x E+00 | 2.274 x E-03 |
| *Catonella* | 8.261 x E-01 | 9.130 x E-01 | 2.216 x E-07 | 1.000 x E+00 | 1.000 x E+00 | 1.148 x E-06 | 1.000 x E+00 | 1.106 x E-06 | 1.000 x E+00 | 2.294 x E-05 |
| *Centipeda / Selenomonas (mixed species)* | 1.000 x E+00 | 2.180 x E-11 | 1.000 x E+00 | 3.980 x E-03 | 2.513 x E-08 | 1.000 x E+00 | 3.966 x E-04 | 2.494 x E-04 | 1.000 x E+00 | 4.136 x E-03 |
| *Dialister* | 1.000 x E+00 | 7.126 x E-01 | 4.706 x E-16 | 1.000 x E+00 | 1.000 x E+00 | 1.216 x E-12 | 1.000 x E+00 | 2.700 x E-14 | 1.000 x E+00 | 5.376 x E-11 |
| *Eikenella* | 7.376 x E-01 | 3.778 x E-01 | 1.435 x E-24 | 1.000 x E+00 | 1.000 x E+00 | 2.488 x E-17 | 1.000 x E+00 | 2.227 x E-20 | 1.000 x E+00 | 3.110 x E-14 |
| *Escherichia* | 2.311 x E-06 | 7.126 x E-01 | 1.000 x E+00 | 1.000 x E+00 | 3.687 x E-08 | 1.650 x E-03 | 2.023 x E-03 | 1.000 x E+00 | 1.000 x E+00 | 1.000 x E+00 |
| *Escherichia / Shigella (mixed species)* | 7.225 x E-01 | 3.470 x E-10 | 1.729 x E-02 | 5.230 x E-02 | 8.002 x E-08 | 2.649 x E-03 | 3.488 x E-03 | 1.000 x E+00 | 1.000 x E+00 | 1.000 x E+00 |
| *Filifactor* | 9.103 x E-10 | 7.126 x E-01 | 9.999 x E-01 | 1.000 x E+00 | 1.135 x E-11 | 1.899 x E-05 | 3.210 x E-05 | 1.000 x E+00 | 1.000 x E+00 | 1.000 x E+00 |
| *Fusobacterium* | 1.000 x E+00 | 7.126 x E-01 | 1.885 x E-01 | 3.980 x E-03 | 1.000 x E+00 | 2.161 x E-01 | 4.907 x E-03 | 6.177 x E-01 | 2.909 x E-04 | 1.456 x E-04 |
| *Gemmiger* | 2.509 x E-06 | 7.126 x E-01 | 1.000 x E+00 | 1.000 x E+00 | 3.123 x E-08 | 1.561 x E-03 | 1.947 x E-03 | 1.000 x E+00 | 1.000 x E+00 | 1.000 x E+00 |
| *Gemmiger / Subdoligranulum (mixed species)* | 3.725 x E-01 | 6.269 x E-10 | 9.136 x E-02 | 7.345 x E-02 | 1.397 x E-08 | 6.928 x E-03 | 2.308 x E-03 | 8.156 x E-01 | 1.000 x E+00 | 1.000 x E+00 |
| *Helicobacter* | 4.498 x E-14 | 1.000 x E+00 | 9.999 x E-01 | 1.110 x E-02 | 6.457 x E-14 | 1.072 x E-05 | 7.896 x E-02 | 1.000 x E+00 | 7.965 x E-03 | 9.708 x E-02 |
| *Hoylesella* | 8.743 x E-01 | 7.126 x E-01 | 2.686 x E-22 | 1.000 x E+00 | 1.000 x E+00 | 2.432 x E-16 | 1.000 x E+00 | 1.814 x E-19 | 1.000 x E+00 | 3.110 x E-14 |
| *Lachnoanaerobaculum* | 8.743 x E-01 | 9.618 x E-01 | 4.325 x E-02 | 1.000 x E+00 | 1.000 x E+00 | 3.231 x E-02 | 1.000 x E+00 | 9.377 x E-02 | 1.000 x E+00 | 9.850 x E-02 |
| *Lactobacillus* | 6.610 x E-02 | 2.786 x E-07 | 2.924 x E-03 | 1.000 x E+00 | 1.000 x E+00 | 4.169 x E-01 | 2.000 x E-02 | 8.649 x E-01 | 6.310 x E-04 | 2.251 x E-03 |
| *Leyella* | 7.225 x E-01 | 3.470 x E-10 | 2.849 x E-02 | 3.792 x E-02 | 8.255 x E-08 | 4.578 x E-03 | 2.464 x E-03 | 1.000 x E+00 | 1.000 x E+00 | 1.000 x E+00 |
| *Ligilactobacillus* | 8.297 x E-01 | 9.806 x E-01 | 2.418 x E-03 | 5.669 x E-03 | 1.000 x E+00 | 2.082 x E-02 | 2.042 x E-02 | 3.200 x E-03 | 5.711 x E-03 | 1.000 x E+00 |
| *Limosilactobacillus* | 7.376 x E-01 | 4.193 x E-01 | 1.000 x E+00 | 5.487 x E-01 | 1.000 x E+00 | 1.000 x E+00 | 1.089 x E-01 | 9.271 x E-01 | 4.858 x E-02 | 3.059 x E-01 |
| *Mammaliicoccus / Staphylococcus (mixed species)* | 1.771 x E-01 | 4.825 x E-02 | 4.876 x E-01 | 1.000 x E+00 | 1.000 x E+00 | 1.000 x E+00 | 1.000 x E+00 | 1.000 x E+00 | 1.000 x E+00 | 1.000 x E+00 |
| *Mediterraneibacter* | 2.180 x E-04 | 4.464 x E-11 | 1.000 x E+00 | 1.000 x E+00 | 1.000 x E+00 | 3.440 x E-03 | 4.230 x E-03 | 2.204 x E-04 | 6.150 x E-04 | 1.000 x E+00 |
| *Moraxella* | 3.691 x E-08 | 4.900 x E-13 | 2.418 x E-03 | 1.000 x E+00 | 1.000 x E+00 | 1.000 x E+00 | 3.385 x E-04 | 1.000 x E+00 | 3.723 x E-04 | 1.102 x E-02 |
| *Neisseria* | 9.997 x E-01 | 3.494 x E-01 | 4.325 x E-02 | 1.000 x E+00 | 1.000 x E+00 | 9.991 x E-02 | 1.000 x E+00 | 4.970 x E-01 | 1.000 x E+00 | 1.314 x E-01 |
| *Olsenella* | 1.945 x E-06 | 9.618 x E-01 | 9.640 x E-01 | 1.000 x E+00 | 8.638 x E-06 | 7.175 x E-04 | 1.150 x E-03 | 8.649 x E-01 | 1.000 x E+00 | 1.000 x E+00 |
| *Parvimonas / Peptostreptococcus (mixed species)* | 1.000 x E+00 | 7.126 x E-01 | 9.665 x E-05 | 1.000 x E+00 | 1.000 x E+00 | 5.428 x E-04 | 1.000 x E+00 | 5.771 x E-04 | 1.000 x E+00 | 3.191 x E-03 |
| *Peptoanaerobacter* | 7.790 x E-05 | 1.585 x E-17 | 1.729 x E-02 | 1.000 x E+00 | 6.613 x E-01 | 1.000 x E+00 | 1.947 x E-03 | 4.458 x E-01 | 5.468 x E-06 | 2.017 x E-02 |
| *Peptostreptococcus* | 8.874 x E-01 | 4.779 x E-01 | 2.924 x E-03 | 1.000 x E+00 | 1.000 x E+00 | 1.916 x E-02 | 9.039 x E-01 | 5.471 x E-02 | 5.640 x E-01 | 6.591 x E-03 |
| *Phascolarctobacterium* | 1.627 x E-07 | 1.000 x E+00 | 1.000 x E+00 | 1.000 x E+00 | 6.691 x E-10 | 7.324 x E-04 | 1.150 x E-03 | 1.000 x E+00 | 1.000 x E+00 | 1.000 x E+00 |
| *Prevotella* | 7.376 x E-01 | 1.000 x E+00 | 3.381 x E-01 | 5.487 x E-01 | 1.000 x E+00 | 9.991 x E-02 | 9.186 x E-01 | 3.764 x E-01 | 4.764 x E-01 | 3.497 x E-02 |
| *Prevotella / Segatella (mixed species)* | 9.540 x E-01 | 9.806 x E-01 | 9.357 x E-13 | 1.000 x E+00 | 1.000 x E+00 | 1.534 x E-10 | 1.000 x E+00 | 3.063 x E-12 | 1.000 x E+00 | 3.554 x E-08 |
| *Prevotellamassilia* | 4.187 x E-06 | 1.000 x E+00 | 1.000 x E+00 | 1.000 x E+00 | 2.513 x E-08 | 1.530 x E-03 | 1.947 x E-03 | 1.000 x E+00 | 1.000 x E+00 | 1.000 x E+00 |
| *Pseudomonas* | 1.746 x E-02 | 4.193 x E-01 | 2.905 x E-02 | 7.735 x E-01 | 6.613 x E-01 | 1.000 x E+00 | 1.000 x E+00 | 3.439 x E-01 | 1.000 x E+00 | 9.368 x E-01 |
| *Pseudostreptobacillus* | 5.100 x E-05 | 7.126 x E-01 | 6.709 x E-03 | 1.000 x E+00 | 3.115 x E-06 | 1.000 x E+00 | 5.832 x E-04 | 1.425 x E-03 | 1.000 x E+00 | 4.136 x E-03 |
| *Roseburia* | 1.210 x E-07 | 1.000 x E+00 | 1.000 x E+00 | 1.000 x E+00 | 1.385 x E-13 | 3.410 x E-04 | 5.832 x E-04 | 1.000 x E+00 | 1.000 x E+00 | 1.000 x E+00 |
| *Ruminococcoides* | 1.738 x E-06 | 1.694 x E-08 | 1.000 x E+00 | 1.000 x E+00 | 1.000 x E+00 | 1.764 x E-04 | 3.966 x E-04 | 4.777 x E-04 | 9.268 x E-04 | 1.000 x E+00 |
| *Segatella* | 7.225 x E-01 | 9.735 x E-01 | 5.113 x E-02 | 1.000 x E+00 | 8.054 x E-01 | 9.538 x E-03 | 1.000 x E+00 | 9.841 x E-02 | 1.000 x E+00 | 1.113 x E-01 |
| *Selenomonas* | 7.713 x E-02 | 7.126 x E-01 | 1.172 x E-13 | 7.735 x E-01 | 5.584 x E-01 | 1.268 x E-15 | 1.000 x E+00 | 3.660 x E-15 | 1.000 x E+00 | 1.217 x E-11 |
| *Shuttleworthella* | 1.738 x E-06 | 5.662 x E-17 | 1.618 x E-03 | 1.000 x E+00 | 1.000 x E+00 | 1.000 x E+00 | 1.947 x E-03 | 1.000 x E+00 | 1.478 x E-04 | 1.234 x E-02 |
| *Solobacterium* | 9.997 x E-01 | 7.126 x E-01 | 5.025 x E-20 | 1.000 x E+00 | 1.000 x E+00 | 2.072 x E-15 | 1.000 x E+00 | 6.936 x E-18 | 1.000 x E+00 | 6.577 x E-13 |
| *Tannerella* | 5.619 x E-15 | 9.806 x E-01 | 6.154 x E-06 | 1.000 x E+00 | 3.192 x E-15 | 1.000 x E+00 | 2.693 x E-07 | 4.279 x E-06 | 1.000 x E+00 | 3.027 x E-04 |
| *species:[Eubacterium] nodatum* | 4.258 x E-08 | 9.159 x E-01 | 9.834 x E-01 | 3.980 x E-03 | 4.430 x E-09 | 1.448 x E-04 | 1.000 x E+00 | 1.000 x E+00 | 6.310 x E-04 | 6.354 x E-03 |
| *species:[Eubacterium] sulci* | 1.000 x E+00 | 9.806 x E-01 | 1.882 x E-10 | 1.000 x E+00 | 1.000 x E+00 | 4.175 x E-08 | 1.000 x E+00 | 9.545 x E-11 | 1.000 x E+00 | 3.846 x E-07 |

*Adjusted p-values obtained from DESeq2 differential abundance analysis for genera identified in pairwise comparisons among diagnosis groups from biopsy tissues in the MAGIC cohort. Only genera showing statistically significant differential abundance in at least one pairwise comparison are shown. A total of 73 tissues passing quality-control filters were included in the analysis. SG = Superficial Gastritis, AG = Atrophic Gastritis, IM = Intestinal Metaplasia, DY = Dysplasia, GC = Gastric Cancer.*

**Supplementary Table 6. Differentially abundant genera between GC and non-GC groups from the MAGIC cohort.**

| ***Genus*** | **log₂FC** | **Adjusted p-value** |
| --- | --- | --- |
| *Bradyrhizobium* | -25.60 | 3.386 x E-19 |
| *Brevundimonas* | -22.61 | 7.433 x E-09 |
| *Escherichia / Shigella (mixed species)* | -22.52 | 8.047 x E-09 |
| *Acidovorax* | -22.46 | 8.391 x E-09 |
| *Moraxella* | -22.18 | 1.283 x E-08 |
| *Mediterraneibacter* | -22.05 | 1.490 x E-08 |
| *Pseudoglutamicibacter* | -3.27 | 3.153 x E-02 |
| *Helicobacter* | -3.18 | 6.041 x E-03 |
| *Rothia* | -1.88 | 1.038 x E-02 |
| *Enterococcus* | 2.03 | 4.186 x E-02 |
| *Campylobacter* | 2.95 | 4.186 x E-02 |
| *Prevotella* | 3.63 | 1.603 x E-09 |
| *Lachnoanaerobaculum* | 3.87 | 9.861 x E-03 |
| *Porphyromonas* | 3.91 | 9.901 x E-04 |
| *Alloprevotella* | 4.12 | 1.470 x E-02 |
| *Lancefieldella* | 4.52 | 3.355 x E-03 |
| *Fusobacterium* | 4.74 | 5.437 x E-10 |
| *Segatella* | 4.90 | 4.784 x E-07 |
| *Peptostreptococcus* | 5.74 | 4.131 x E-06 |
| *Selenomonas* | 5.75 | 1.910 x E-03 |
| *Carnobacterium* | 6.71 | 1.781 x E-02 |
| *Treponema* | 7.46 | 6.372 x E-05 |
| *Dialister* | 7.62 | 1.159 x E-06 |
| *Curtanaerobium* | 9.36 | 4.561 x E-02 |
| *Olsenella* | 10.31 | 6.973 x E-03 |
| *Limosilactobacillus* | 11.71 | 1.904 x E-17 |
| *Lactobacillus* | 12.53 | 7.414 x E-33 |
| *Centipeda / Selenomonas (mixed species)* | 26.86 | 2.814 x E-12 |
| *Allisonella* | 29.94 | 3.267 x E-15 |
| *Lactobacillus / Limosilactobacillus (mixed species)* | 30.00 | 1.678 x E-21 |
| *Pseudoramibacter* | 30.00 | 3.209 x E-15 |
| *Alterileibacterium* | 30.00 | 3.209 x E-15 |
| *Shuttleworthella* | 30.00 | 3.209 x E-15 |

*Log₂ fold change (log₂FC) values for genera showing significant differential abundance between GC and non-GC groups in the MAGIC cohort. Only genera with at least one statistically significant comparison (adjusted p-value < 0.05) are included. Positive log₂FC values indicate increased abundance in GC tissues, whereas negative values indicate decreased abundance in GC tissues. A total of 86 tissues passing quality-control filters were included in the analysis. The color gradient indicates the magnitude and direction of the log₂FC values, ranging from decreased abundance (red) to increased abundance (green) in GC. GC = Gastric cancer.*

**Supplementary Table 7.** **Differential abundance signatures through pairwise comparisons in histologycal subtypes in the BTUCH Cohort.**

| **genus** | **log₂FC** | | |
| --- | --- | --- | --- |
|  | **Diffuse_vs_Intestinal** | **Diffuse vs Mixed** | **Intestinal vs Mixed** |
| *Escherichia* | -6.54 | -1.18 | 5.36 |
| *Clostridium* | -4.85 | -1.06 | 3.79 |
| *Parvimonas* | -4.09 | -0.98 | 3.11 |
| *Enterococcus* | -2.61 | -0.58 | 2.03 |
| *Vagococcus* | -1.87 | 19.63 | 21.51 |
| *Tannerella* | 0.17 | 19.81 | 19.64 |
| *Gemella* | 2.21 | 3.15 | 0.94 |
| *Haemophilus* | 2.65 | -3.72 | -6.37 |
| *Leptotrichia* | 3.23 | 19.86 | 16.64 |
| *Oribacterium* | 3.42 | 21.25 | 17.83 |
| *Neisseria* | 3.65 | 3.98 | 0.33 |
| *Campylobacter* | 4.13 | 22.49 | 18.36 |
| *Lancefieldella* | 5.54 | 22.37 | 16.84 |
| *Helicobacter* | 6.39 | 6.70 | 0.32 |
| *Hoylesella* | 6.51 | 22.62 | 16.11 |
| *Lautropia* | 21.90 | 18.93 | 0.00 |
| *Corynebacterium* | 21.94 | -1.00 | -22.95 |
| *Pseudomonas* | 22.66 | 1.97 | -20.69 |
| *Capnocytophaga* | 23.37 | 20.79 | -2.58 |
| *Solobacterium* | 24.57 | 4.00 | -20.57 |

*Log₂ fold change (log₂FC) matrix for differentially abundant genera by histology subtypes in the BTUCH cohort based on DESeq2 analysis of genus-level count data. Positive log₂FC values indicate relative enrichment in the first group of each comparison, whereas negative values indicate relative depletion. A total of 52 tissues passing quality-control filters were included in the analysis. Only genera showing at least one statistically significant difference (adjusted p-value < 0.05) in any pairwise comparison are included in the table.*

**Supplementary Table 8. Differentially abundant genera between GC and non-GC groups from both MAGIC and BTUCH cohorts.**

| **genus** | **log₂FC** | **Adjusted p-value** |
| --- | --- | --- |
| *species:[Eubacterium] brachy* | -4.84 | 6.205 x E-03 |
| *Streptococcus* | 0.99 | 2.988 x E-03 |
| *Prevotella* | 1.56 | 5.037 x E-05 |
| *Gemella* | 1.60 | 5.037 x E-05 |
| *Peptostreptococcus* | 1.88 | 3.187 x E-02 |
| *Helicobacter* | 2.20 | 8.332 x E-04 |
| *Fusobacterium* | 2.36 | 4.535 x E-04 |
| *Porphyromonas* | 2.60 | 6.466 x E-04 |
| *Parvimonas* | 2.79 | 6.101 x E-03 |
| *Catonella* | 3.13 | 5.444 x E-02 |
| *Phocaeicola* | 3.60 | 3.513 x E-03 |
| *Treponema* | 3.65 | 3.345 x E-02 |
| *Dialister* | 3.67 | 6.101 x E-03 |
| *Escherichia / Shigella (mixed species)* | 4.20 | 3.513 x E-03 |
| *Enterococcus* | 4.26 | 1.340 x E-19 |
| *Bacteroides* | 4.36 | 1.317 x E-02 |
| *Carnobacterium* | 4.89 | 2.505 x E-02 |
| *Ligilactobacillus* | 4.96 | 3.513 x E-03 |
| *Pseudostreptobacillus* | 5.64 | 3.187 x E-02 |
| *Enterobacter* | 5.95 | 1.387 x E-02 |
| *Blautia* | 6.71 | 1.676 x E-03 |
| *Serratia* | 7.12 | 7.634 x E-03 |
| *Kosakonia* | 7.53 | 3.345 x E-02 |
| *Kluyvera* | 7.92 | 2.505 x E-02 |
| *Sphingomonas* | 8.31 | 4.978 x E-05 |
| *Komagataeibacter* | 8.46 | 6.193 x E-03 |
| *Olsenella* | 8.59 | 1.217 x E-02 |
| *Lactiplantibacillus* | 8.63 | 1.854 x E-04 |
| *Klebsiella* | 9.12 | 6.388 x E-24 |
| *Coprococcus* | 9.18 | 1.318 x E-06 |
| *Enterococcus / Lacticaseibacillus (mixed species)* | 10.39 | 2.094 x E-03 |
| *Clostridium* | 10.77 | 1.093 x E-27 |
| *Limosilactobacillus* | 10.80 | 4.626 x E-50 |
| *Lactobacillus* | 11.34 | 9.553 x E-79 |
| *Lacticaseibacillus* | 14.01 | 1.321 x E-24 |
| *Sarcina* | 14.81 | 7.145 x E-86 |
| *Shuttleworthella* | 27.01 | 1.340 x E-19 |
| *Escherichia* | 28.27 | 1.576 x E-50 |
| *Aeromonas* | 28.93 | 2.348 x E-22 |
| *Lactobacillus / Limosilactobacillus (mixed species)* | 30.00 | 3.570 x E-66 |
| *Weissella* | 30.00 | 2.365 x E-46 |
| *Pseudoramibacter* | 30.00 | 6.388 x E-24 |

*Log₂ fold change (log₂FC) values for genera showing significant differential abundance between GC and non-GC groups across the MAGIC and BTUCH cohorts. Positive log₂FC values indicate increased abundance in GC tissues, whereas negative values indicate decreased abundance in GC tissues. A total of 138 tissues passing quality-control filters were included in the analysis. Only genera with at least one statistically significant difference (adjusted p-value < 0.05) in any comparison are included. The color gradient represents the magnitude and direction of log₂FC values, ranging from decreased abundance in GC (red) to increased abundance in GC (green). GC = Gastric cancer.*

**Supplementary Table 9. Spearman correlation analysis between bacterial genus-level relative abundances and progression stages along Correa Cascade (SG to GC).**

| ***Genus*** | **Correlation coefficients (ρ)** | **Adjusted p-value** |
| --- | --- | --- |
| *Staphylococcus* | -0.49 | 1.3 x E-08 |
| *Veillonella* | -0.48 | 3.7 x E-08 |
| *Rothia* | -0.42 | 3.3 x E-06 |
| *Fusobacterium* | -0.41 | 4.6 x E-06 |
| *Schaalia* | -0.40 | 7.4 x E-06 |
| *Stomatobaculum* | -0.40 | 9.2 x E-06 |
| *Haemophilus* | -0.40 | 9.2 x E-06 |
| *Actinomyces* | -0.37 | 5.5 x E-05 |
| *Actinomyces / Schaalia (mixed species)* | -0.36 | 7.0 x E-05 |
| *Oribacterium* | -0.36 | 7.3 x E-05 |
| *Mannheimia* | -0.35 | 9.5 x E-05 |
| *Enterococcus / Granulicatella (mixed species)* | -0.35 | 9.7 x E-05 |
| *Granulicatella / Vagococcus (mixed species)* | -0.34 | 1.6 x E-04 |
| *Granulicatella* | -0.34 | 1.7 x E-04 |
| *Vagococcus* | -0.33 | 2.2 x E-04 |
| *Mogibacterium* | -0.33 | 2.2 x E-04 |
| *Megasphaera* | -0.33 | 2.3 x E-04 |
| *Leptotrichia* | -0.33 | 2.7 x E-04 |
| *Pseudomonas* | -0.32 | 4.8 x E-04 |
| *Neisseria* | -0.31 | 6.3 x E-04 |
| *Lactiplantibacillus* | 0.31 | 5.2 x E-04 |
| *Sphingomonas* | 0.32 | 4.8 x E-04 |
| *Weissella* | 0.35 | 1.1 x E-04 |
| *Bacteroides* | 0.38 | 3.2 x E-05 |
| *Lacticaseibacillus* | 0.39 | 1.4 x E-05 |
| *Lactobacillus / Limosilactobacillus (mixed species)* | 0.39 | 1.0 x E-05 |
| *Phocaeicola* | 0.41 | 6.6 x E-06 |
| *Escherichia / Shigella (mixed species)* | 0.43 | 1.7 x E-06 |
| *Klebsiella* | 0.59 | 8.6 x E-13 |
| *Limosilactobacillus* | 0.62 | 2.3 x E-14 |
| *Clostridium* | 0.68 | 2.9 x E-18 |
| *Lactobacillus* | 0.68 | 2.9 x E-18 |
| *Sarcina* | 0.70 | 9.3 x E-20 |

*Spearman correlation analysis between bacterial genus-level relative abundances in progression stages of disease in the MAGIC cohort. Correlation coefficients (ρ) were calculated across histopathological stages ranging from SG to GC. Positive ρ values indicate increasing relative abundance along disease progression, whereas negative ρ values indicate decreasing abundance. A total of 138 tissues passing quality-control filters were included in the analysis. Only genera showing statistically significant correlations (adjusted p-value < 0.05 and |ρ| ≥ 0.30) are included in the table. SG = superficial gastritis; IM = intestinal metaplasia.*

**Supplementary Table 10.** ***Helicobacter* status across gastric disease stages along the Correa cascade**

| **Group** | **H-** | **H+** | **Total** |
| --- | --- | --- | --- |
| SG | 20 | 9 | 29 |
| AG | 8 | 0 | 8 |
| IM | 17 | 11 | 28 |
| DY | 2 | 2 | 4 |
| GC | 38 | 31 | 69 |

*Helicobacter status across gastric disease stages along the Correa cascade based on sequencing-derived relative abundance data. Tissues were classified as Helicobacter-positive when the relative abundance exceeded the predefined threshold of 1%. H+ = Helicobacter-positive, H - = Helicobacter-negative. SG = Superficial Gastritis, AG = Atrophic Gastritis, IM = Intestinal Metaplasia, DY = Dysplasia, GC = Gastric Cancer.*

**Supplementary Table 11. Spearman correlation analysis between bacterial genus-level relative abundances in SG by *Helicobacter* status.**

| ***Genus*** | **Correlation coefficients (ρ)** | **p-value** | **Adjusted p-value** |
| --- | --- | --- | --- |
| *Cutibacterium* | -0.65 | 1.404 x E-04 | 1.278 x E-02 |
| *Pseudomonas* | -0.59 | 7.875 x E-04 | 3.583 x E-02 |
| *Hoylesella* | -0.52 | 3.530 x E-03 | 8.221 x E-02 |
| *Fusobacterium* | -0.51 | 4.493 x E-03 | 8.221 x E-02 |
| *Actinobacillus / Aggregatibacter (mixed species)* | -0.50 | 5.454 x E-03 | 8.221 x E-02 |
| *Enterococcus* | -0.50 | 5.500 x E-03 | 8.221 x E-02 |
| *Haemophilus* | -0.50 | 6.324 x E-03 | 8.221 x E-02 |
| *Delftia* | -0.48 | 8.808 x E-03 | 9.268 x E-02 |
| *Morococcus* | -0.48 | 9.166 x E-03 | 9.268 x E-02 |
| *Treponema* | -0.47 | 1.038 x E-02 | 9.446 x E-02 |
| *Prevotella* | -0.44 | 1.681 x E-02 | 1.298 x E-01 |
| *Moraxella* | -0.44 | 1.711 x E-02 | 1.298 x E-01 |
| *Bradyrhizobium* | -0.43 | 2.084 x E-02 | 1.355 x E-01 |
| *species:[Eubacterium] nodatum* | 0.43 | 2.036 x E-02 | 1.355 x E-01 |
| *Morococcus / Neisseria (mixed species)* | -0.42 | 2.454 x E-02 | 1.489 x E-01 |
| *Segatella* | -0.41 | 2.633 x E-02 | 1.498 x E-01 |
| *Aggregatibacter / Haemophilus (mixed species)* | -0.40 | 3.254 x E-02 | 1.742 x E-01 |
| *Kocuria* | -0.39 | 3.681 x E-02 | 1.772 x E-01 |
| *Actinobacillus / Haemophilus (mixed species)* | -0.39 | 3.827 x E-02 | 1.772 x E-01 |
| *Neisseria* | -0.39 | 3.895 x E-02 | 1.772 x E-01 |
| *Eikenella / Neisseria (mixed species)* | -0.37 | 4.770 x E-02 | 2.003 x E-01 |
| *Eikenella* | -0.37 | 4.841 x E-02 | 2.003 x E-01 |
| *Lautropia* | -0.36 | 5.703 x E-02 | 2.220 x E-01 |
| *Streptococcus* | -0.35 | 5.942 x E-02 | 2.220 x E-01 |
| *Propionibacterium* | 0.35 | 6.098 x E-02 | 2.220 x E-01 |
| *Alloprevotella* | -0.35 | 6.472 x E-02 | 2.265 x E-01 |
| *Tannerella* | -0.34 | 6.745 x E-02 | 2.273 x E-01 |
| *Massilia* | -0.34 | 7.010 x E-02 | 2.278 x E-01 |
| *Pseudoglutamicibacter / Psychromicrobium (mixed species)* | -0.33 | 7.940 x E-02 | 2.408 x E-01 |
| *Cardiobacterium* | 0.33 | 7.835 x E-02 | 2.408 x E-01 |
| *Aggregatibacter* | -0.32 | 9.154 x E-02 | 2.687 x E-01 |
| *Actinobacillus* | -0.31 | 9.751 x E-02 | 2.773 x E-01 |
| *Staphylococcus* | -0.31 | 1.034 x E-01 | 2.853 x E-01 |
| *Arthrobacter* | -0.30 | 1.099 x E-01 | 2.943 x E-01 |
| *Kocuria / Rothia (mixed species)* | 0.29 | 1.292 x E-01 | 3.359 x E-01 |
| *Carnobacterium* | -0.28 | 1.367 x E-01 | 3.456 x E-01 |
| *Aggregatibacter / Mannheimia (mixed species)* | -0.27 | 1.519 x E-01 | 3.674 x E-01 |
| *Mannheimia* | -0.27 | 1.570 x E-01 | 3.674 x E-01 |
| *Faucicola* | -0.27 | 1.586 x E-01 | 3.674 x E-01 |
| *Granulicatella / Streptococcus (mixed species)* | -0.27 | 1.615 x E-01 | 3.674 x E-01 |
| *Capnocytophaga* | -0.26 | 1.722 x E-01 | 3.785 x E-01 |
| *Gemella* | -0.26 | 1.747 x E-01 | 3.785 x E-01 |
| *Bavariicoccus / Granulicatella (mixed species)* | -0.26 | 1.801 x E-01 | 3.812 x E-01 |
| *Abiotrophia* | -0.25 | 1.868 x E-01 | 3.863 x E-01 |
| *Stomatobaculum* | 0.25 | 1.974 x E-01 | 3.992 x E-01 |
| *Sphingobium* | -0.24 | 2.102 x E-01 | 4.078 x E-01 |
| *Bacillus* | -0.24 | 2.109 x E-01 | 4.078 x E-01 |
| *Actinomyces* | -0.23 | 2.206 x E-01 | 4.078 x E-01 |
| *Campylobacter* | -0.23 | 2.284 x E-01 | 4.078 x E-01 |
| *Peptostreptococcus* | -0.23 | 2.285 x E-01 | 4.078 x E-01 |
| *Limosilactobacillus* | 0.24 | 2.179 x E-01 | 4.078 x E-01 |
| *Corynebacterium* | -0.22 | 2.619 x E-01 | 4.583 x E-01 |
| *Granulicatella* | -0.21 | 2.705 x E-01 | 4.644 x E-01 |
| *Enterococcus / Granulicatella (mixed species)* | -0.21 | 2.809 x E-01 | 4.712 x E-01 |
| *Pseudoglutamicibacter* | -0.20 | 2.913 x E-01 | 4.712 x E-01 |
| *Bavariicoccus* | -0.20 | 2.934 x E-01 | 4.712 x E-01 |
| *Citrobacter* | -0.20 | 2.951 x E-01 | 4.712 x E-01 |
| *Actinomyces / Schaalia (mixed species)* | -0.20 | 3.069 x E-01 | 4.815 x E-01 |
| *Enterococcus / Vagococcus (mixed species)* | -0.19 | 3.258 x E-01 | 5.025 x E-01 |
| *Lacticaseibacillus* | -0.18 | 3.393 x E-01 | 5.085 x E-01 |
| *Mogibacterium* | -0.18 | 3.409 x E-01 | 5.085 x E-01 |
| *Acidovorax* | -0.18 | 3.504 x E-01 | 5.143 x E-01 |
| *Klebsiella* | -0.18 | 3.634 x E-01 | 5.250 x E-01 |
| *Porphyromonas* | -0.16 | 4.178 x E-01 | 5.940 x E-01 |
| *Vagococcus* | -0.14 | 4.534 x E-01 | 6.241 x E-01 |
| *Prevotella / Segatella (mixed species)* | -0.14 | 4.541 x E-01 | 6.241 x E-01 |
| *Enterobacter* | -0.14 | 4.595 x E-01 | 6.241 x E-01 |
| *Veillonella* | -0.13 | 4.948 x E-01 | 6.622 x E-01 |
| *Lactobacillus* | 0.12 | 5.273 x E-01 | 6.954 x E-01 |
| *Leptotrichia* | -0.12 | 5.467 x E-01 | 7.106 x E-01 |
| *Granulicatella / Vagococcus (mixed species)* | -0.11 | 5.600 x E-01 | 7.178 x E-01 |
| *Ligilactobacillus* | 0.11 | 5.697 x E-01 | 7.201 x E-01 |
| *Metamycoplasma* | -0.11 | 5.872 x E-01 | 7.320 x E-01 |
| *Rodentibacter* | -0.09 | 6.298 x E-01 | 7.745 x E-01 |
| *Catonella* | -0.09 | 6.610 x E-01 | 8.020 x E-01 |
| *Rothia* | -0.08 | 6.901 x E-01 | 8.263 x E-01 |
| *Parvimonas / Peptostreptococcus (mixed species)* | -0.06 | 7.661 x E-01 | 9.054 x E-01 |
| *species:[Eubacterium] brachy* | -0.05 | 8.064 x E-01 | 9.097 x E-01 |
| *Dialister* | -0.05 | 8.070 x E-01 | 9.097 x E-01 |
| *Solobacterium* | -0.05 | 8.085 x E-01 | 9.097 x E-01 |
| *Lachnoanaerobaculum* | -0.04 | 8.206 x E-01 | 9.097 x E-01 |
| *Oribacterium* | 0.04 | 8.298 x E-01 | 9.097 x E-01 |
| *Lancefieldella* | 0.04 | 8.175 x E-01 | 9.097 x E-01 |
| *Selenomonas* | -0.04 | 8.458 x E-01 | 9.140 x E-01 |
| *species:[Eubacterium] sulci* | 0.03 | 8.638 x E-01 | 9.140 x E-01 |
| *Parvimonas* | 0.04 | 8.552 x E-01 | 9.140 x E-01 |
| *Megasphaera* | 0.03 | 8.786 x E-01 | 9.189 x E-01 |
| *Mammaliicoccus / Staphylococcus (mixed species)* | -0.01 | 9.539 x E-01 | 9.864 x E-01 |
| *Kingella* | 0.01 | 9.657 x E-01 | 9.874 x E-01 |
| *Schaalia* | 0.00 | 9.949 x E-01 | 9.962 x E-01 |
| *Olsenella* | 0.00 | 9.962 x E-01 | 9.962 x E-01 |

*Spearman correlation analysis between bacterial genus-level relative abundances in SG tissues according to Helicobacter status. Helicobacter positivity was defined using a sequencing-derived relative abundance threshold of 1%. Positive correlation coefficients (ρ) indicate genera associated with Helicobacter-positive tissues, whereas negative values indicate association with Helicobacter-negative tissues. A total of 29 tissues passing quality-control filters were included in the analysis. Only correlations with adjusted p-value < 0.05 are shown. SG = superficial gastritis.*

**Supplementary Table 12. Spearman correlation analysis between bacterial genus-level relative abundances in IM by *Helicobacter* status.**

| ***Genus*** | **Correlation coefficients (ρ)** | **p-value** | **Adjusted p-value** |
| --- | --- | --- | --- |
| *Actinomyces* | -0.76 | 3.1 x E-06 | 1.4 x E-04 |
| *Streptococcus* | -0.76 | 3.2 x E-06 | 1.4 x E-04 |
| *Veillonella* | -0.74 | 6.6 x E-06 | 1.9 x E-04 |
| *Alloscardovia* | -0.69 | 5.4 x E-05 | 1.2 x E-03 |
| *Stomatobaculum* | -0.67 | 8.6 x E-05 | 1.3 x E-03 |
| *Fusobacterium* | -0.67 | 8.8 x E-05 | 1.3 x E-03 |
| *Leptotrichia* | -0.66 | 1.5 x E-04 | 1.8 x E-03 |
| *Enterococcus / Granulicatella (mixed species)* | -0.61 | 5.8 x E-04 | 5.8 x E-03 |
| *Granulicatella / Vagococcus (mixed species)* | -0.61 | 6.0 x E-04 | 5.8 x E-03 |
| *Solobacterium* | -0.59 | 1.0 x E-03 | 8.5 x E-03 |
| *Granulicatella* | -0.58 | 1.1 x E-03 | 8.5 x E-03 |
| *Segatella* | -0.58 | 1.2 x E-03 | 8.5 x E-03 |
| *Vagococcus* | -0.58 | 1.3 x E-03 | 8.5 x E-03 |
| *Lachnoanaerobaculum* | -0.57 | 1.5 x E-03 | 9.3 x E-03 |
| *Gemella* | -0.57 | 1.7 x E-03 | 9.3 x E-03 |
| *Prevotella* | -0.56 | 1.8 x E-03 | 9.3 x E-03 |
| *Enterococcus / Vagococcus (mixed species)* | -0.56 | 1.8 x E-03 | 9.3 x E-03 |
| *Actinomyces / Schaalia (mixed species)* | -0.55 | 2.2 x E-03 | 1.1 x E-02 |
| *Lancefieldella* | -0.54 | 2.9 x E-03 | 1.3 x E-02 |
| *Schaalia* | -0.52 | 4.3 x E-03 | 1.9 x E-02 |
| *Filifactor* | -0.51 | 5.0 x E-03 | 2.0 x E-02 |
| *Mogibacterium* | -0.51 | 5.2 x E-03 | 2.0 x E-02 |
| *Peptostreptococcus* | -0.51 | 5.3 x E-03 | 2.0 x E-02 |
| *species:[Eubacterium] sulci* | -0.50 | 6.6 x E-03 | 2.3 x E-02 |
| *Parvimonas / Peptostreptococcus (mixed species)* | -0.50 | 6.6 x E-03 | 2.3 x E-02 |
| *Dialister* | -0.49 | 7.5 x E-03 | 2.5 x E-02 |
| *Enterococcus* | -0.48 | 9.0 x E-03 | 2.9 x E-02 |
| *Prevotella / Segatella (mixed species)* | -0.48 | 9.9 x E-03 | 3.1 x E-02 |
| *Bavariicoccus* | -0.45 | 1.5 x E-02 | 4.6 x E-02 |
| *Megasphaera* | -0.45 | 1.7 x E-02 | 4.9 x E-02 |
| *Cryptobacterium* | -0.43 | 2.1 x E-02 | 6.0 x E-02 |
| *species:[Eubacterium] infirmum* | -0.43 | 2.4 x E-02 | 6.4 x E-02 |
| *Parvimonas* | -0.42 | 2.7 x E-02 | 7.0 x E-02 |
| *Neisseria* | -0.42 | 2.8 x E-02 | 7.1 x E-02 |
| *Oribacterium* | -0.40 | 3.3 x E-02 | 8.2 x E-02 |
| *Eikenella / Neisseria (mixed species)* | -0.40 | 3.6 x E-02 | 8.8 x E-02 |
| *Eikenella* | -0.38 | 4.7 x E-02 | 1.1 x E-01 |
| *Pseudoglutamicibacter* | -0.38 | 4.8 x E-02 | 1.1 x E-01 |
| *Porphyromonas* | -0.37 | 5.3 x E-02 | 1.2 x E-01 |
| *Corynebacterium* | -0.35 | 6.8 x E-02 | 1.5 x E-01 |
| *Selenomonas* | -0.35 | 7.1 x E-02 | 1.5 x E-01 |
| *Rothia* | -0.32 | 9.8 x E-02 | 2.0 x E-01 |
| *Lautropia* | -0.31 | 1.1 x E-01 | 2.3 x E-01 |
| *Capnocytophaga* | -0.31 | 1.1 x E-01 | 2.3 x E-01 |
| *species:[Eubacterium] brachy* | -0.30 | 1.2 x E-01 | 2.3 x E-01 |
| *Campylobacter* | -0.30 | 1.2 x E-01 | 2.3 x E-01 |
| *Catonella* | -0.30 | 1.2 x E-01 | 2.3 x E-01 |
| *Tannerella* | -0.29 | 1.3 x E-01 | 2.4 x E-01 |
| *Bulleidia* | -0.29 | 1.3 x E-01 | 2.4 x E-01 |
| *Morococcus / Neisseria (mixed species)* | -0.29 | 1.4 x E-01 | 2.4 x E-01 |
| *Pseudomonas* | -0.27 | 1.7 x E-01 | 2.9 x E-01 |
| *species:[Eubacterium] nodatum* | -0.26 | 1.7 x E-01 | 2.9 x E-01 |
| *Rodentibacter* | -0.26 | 1.9 x E-01 | 3.1 x E-01 |
| *Aggregatibacter / Haemophilus (mixed species)* | -0.25 | 2.0 x E-01 | 3.2 x E-01 |
| *Cutibacterium* | -0.23 | 2.4 x E-01 | 3.7 x E-01 |
| *Alloprevotella* | -0.21 | 2.9 x E-01 | 4.4 x E-01 |
| *Kocuria* | -0.20 | 3.0 x E-01 | 4.6 x E-01 |
| *Massilia* | -0.20 | 3.1 x E-01 | 4.6 x E-01 |
| *Actinobacillus / Aggregatibacter (mixed species)* | -0.18 | 3.5 x E-01 | 5.0 x E-01 |
| *Enterobacter* | -0.18 | 3.5 x E-01 | 5.0 x E-01 |
| *Staphylococcus* | -0.18 | 3.5 x E-01 | 5.0 x E-01 |
| *Haemophilus* | -0.17 | 3.8 x E-01 | 5.4 x E-01 |
| *Arthrobacter* | 0.17 | 4.0 x E-01 | 5.5 x E-01 |
| *Hallella / Prevotella (mixed species)* | -0.16 | 4.2 x E-01 | 5.6 x E-01 |
| *Aggregatibacter / Mannheimia (mixed species)* | -0.16 | 4.2 x E-01 | 5.6 x E-01 |
| *Congobacterium / Mogibacterium (mixed species)* | 0.16 | 4.2 x E-01 | 5.6 x E-01 |
| *Pseudoglutamicibacter / Psychromicrobium (mixed species)* | -0.15 | 4.5 x E-01 | 5.8 x E-01 |
| *Aggregatibacter* | -0.14 | 4.7 x E-01 | 5.8 x E-01 |
| *Phocaeicola* | 0.14 | 4.7 x E-01 | 5.8 x E-01 |
| *Mannheimia* | 0.15 | 4.6 x E-01 | 5.8 x E-01 |
| *Treponema* | -0.13 | 5.2 x E-01 | 6.4 x E-01 |
| *Ligilactobacillus* | -0.12 | 5.3 x E-01 | 6.4 x E-01 |
| *Actinobacillus* | -0.11 | 5.7 x E-01 | 6.7 x E-01 |
| *Pseudostreptobacillus* | -0.11 | 5.7 x E-01 | 6.7 x E-01 |
| *Hoylesella* | -0.10 | 6.2 x E-01 | 7.1 x E-01 |
| *Bavariicoccus / Granulicatella (mixed species)* | -0.10 | 6.2 x E-01 | 7.1 x E-01 |
| *Kingella* | -0.07 | 7.1 x E-01 | 8.0 x E-01 |
| *Klebsiella* | -0.07 | 7.2 x E-01 | 8.0 x E-01 |
| *Mediterraneibacter* | 0.05 | 8.0 x E-01 | 8.8 x E-01 |
| *Morococcus* | -0.04 | 8.2 x E-01 | 8.9 x E-01 |
| *Limosilactobacillus* | 0.04 | 8.4 x E-01 | 9.0 x E-01 |
| *Abiotrophia* | -0.03 | 8.6 x E-01 | 9.0 x E-01 |
| *Citrobacter* | 0.04 | 8.6 x E-01 | 9.0 x E-01 |
| *Lactobacillus* | -0.02 | 9.1 x E-01 | 9.4 x E-01 |
| *Bradyrhizobium* | 0.02 | 9.2 x E-01 | 9.4 x E-01 |
| *Kocuria / Rothia (mixed species)* | 0.00 | 1.0 x E+00 | 1.0 x E+00 |
| *Carnobacterium* | 0.00 | 9.9 x E-01 | 1.0 x E+00 |

*Spearman correlation analysis between bacterial genus-level relative abundances in IM tissues according to Helicobacter status. Helicobacter positivity was defined using a sequencing-derived relative abundance threshold of 1%. Positive correlation coefficients (ρ) indicate genera associated with Helicobacter-positive tissues, whereas negative values indicate association with Helicobacter-negative tissues. A total of 28 tissues passing quality-control filters were included in the analysis. Only correlations with adjusted p-value < 0.05 are shown. IM = intestinal metaplasia.*

**Supplementary Table 13. MetaCyc functional pathways significantly correlated with *Helicobacter* abundance in SG (top 15) and IM (top 15).**

| **Stage** | **Pathway - BioCYC ID** | **Correlation coefficients (ρ)** | **Adjusted p-value** | **Main class ontology** | **Pathway name** |
| --- | --- | --- | --- | --- | --- |
| SG | PWY0-1338 | 0.81 | 3.5 x E-05 | Byosinthesis | polymyxin resistance |
| SG | PWY-7616 | 0.80 | 3.5 x E-05 | Degradation/Utilization/Assimilation | methanol oxidation to carbon dioxide |
| SG | DHGLUCONATE-PYR-CAT-PWY | 0.76 | 1.8 x E-04 | Degradation/Utilization/Assimilation | glucose degradation (oxidative) |
| SG | FAO-PWY | 0.69 | 1.8 x E-03 | Degradation/Utilization/Assimilation | fatty acid β-oxidation I (generic) |
| SG | AST-PWY | 0.65 | 4.1 x E-03 | Degradation/Utilization/Assimilation | L-arginine degradation II (AST pathway) |
| SG | 3-HYDROXYPHENYLACETATE-DEGRADATION-PWY | 0.63 | 5.4 x E-03 | Degradation/Utilization/Assimilation | 4-hydroxyphenylacetate degradation |
| SG | PWY-5028 | -0.64 | 4.9 x E-03 | Degradation/Utilization/Assimilation | L-histidine degradation II |
| SG | PWY0-321 | -0.66 | 2.8 x E-03 | Degradation/Utilization/Assimilation | phenylacetate degradation I (aerobic) |
| SG | PWY-6071 | -0.66 | 2.8 x E-03 | Degradation/Utilization/Assimilation | superpathway of phenylethylamine degradation |
| SG | TRPSYN-PWY | -0.67 | 2.8 x E-03 | Byosinthesis | L-tryptophan biosynthesis |
| SG | PWY0-1296 | -0.67 | 2.8 x E-03 | Degradation/Utilization/Assimilation | purine ribonucleosides degradation |
| SG | PWY-6545 | -0.67 | 2.8 x E-03 | Byosinthesis | pyrimidine deoxyribonucleotides *de novo* biosynthesis III |
| SG | PWY-6263 | -0.69 | 2.1 x E-03 | Byosinthesis | superpathway of menaquinol-8 biosynthesis II |
| SG | PWY-7371 | -0.70 | 1.6 x E-03 | Byosinthesis | 5,8-dihydroxy-2-naphthoate biosynthesis II |
| SG | PWY-7373 | -0.71 | 1.6 x E-03 | Byosinthesis | superpathway of demethylmenaquinol-6 biosynthesis II |
| IM | PWY-3781 | 0.75 | 9.8 x E-04 | Generation of precursor metabolites and energy | aerobic respiration I (cytochrome c) |
| IM | REDCITCYC | 0.74 | 9.8 x E-04 | Generation of precursor metabolites and energy | TCA cycle VI (Helicobacter) |
| IM | PWY-7371 | 0.72 | 1.8 x E-03 | Biosynthesis | 5,8-dihydroxy-2-naphthoate biosynthesis II |
| IM | PWY-7373 | 0.71 | 1.8 x E-03 | Biosynthesis | superpathway of demethylmenaquinol-6 biosynthesis II |
| IM | PWY-6545 | 0.64 | 7.3 x E-03 | Biosynthesis | pyrimidine deoxyribonucleotides *de novo* biosynthesis III |
| IM | PWY0-1241 | 0.64 | 7.3 x E-03 | Biosynthesis | ADP-L-*glycero*-β-D-*manno*-heptose biosynthesis |
| IM | PWY-1861 | -0.63 | 8.9 x E-03 | Degradation/Utilization/Assimilation | formaldehyde assimilation II (assimilatory RuMP Cycle) |
| IM | RUMP-PWY | -0.63 | 7.6 x E-03 | Degradation/Utilization/Assimilation | formaldehyde oxidation I |
| IM | PWY-6478 | -0.64 | 7.3 x E-03 | Biosynthesis | GDP-D-*glycero*-α-D-*manno*-heptose biosynthesis |
| IM | PWY-6749 | -0.65 | 7.3 x E-03 | Biosynthesis | CMP-legionaminate biosynthesis I |
| IM | GALACTUROCAT-PWY | -0.66 | 5.1 x E-03 | Degradation/Utilization/Assimilation | D-galacturonate degradation I |
| IM | SALVADEHYPOX-PWY | -0.67 | 3.8 x E-03 | Degradation/Utilization/Assimilation | adenosine nucleotides degradation II |
| IM | GALACT-GLUCUROCAT-PWY | -0.70 | 1.8 x E-03 | Superpathways | superpathway of hexuronide and hexuronate degradation |
| IM | GLUCUROCAT-PWY | -0.70 | 1.8 x E-03 | Superpathways | superpathway of β-D-glucuronides degradation (to pyruvate) |
| IM | PWY-6353 | -0.71 | 1.8 x E-03 | Degradation/Utilization/Assimilation | purine nucleotides degradation II (aerobic) |

*The table presents the top 15 MetaCyc functional pathways significantly correlated with Helicobacter relative abundance in SG and IM tissues. Correlations were calculated using Spearman analysis between predicted pathway abundances and Helicobacter abundance. Positive correlation coefficients (ρ) indicate pathways positively associated with Helicobacter abundance, whereas negative values indicate inverse associations. Only pathways showing statistically significant correlations (adjusted p-value < 0.05 and |ρ| ≥ 0.30) are included. A total of 29 SG tissues and 28 IM tissues passing quality-control filters were included in the analysis. SG = superficial gastritis, IM = intestinal metaplasia.*

**Supplementary table 14. Metadata and sequencing information for tissues deposited in the Sequence Read Archive (SRA).**

| **n** | **tissue_name** | **accession** | **study** | **bioproject_accession** | **biosample_accession** | **library_source** | **library_selection** | **platform** | **filename** | **isolation_source** | **collection_date** | **geo_loc_name** | **lat_lon** |
| --- | --- | --- | --- | --- | --- | --- | --- | --- | --- | --- | --- | --- | --- |
| 1 | 51519N | SRR37550729 | SRP682544 | PRJNA1434549 | SAMN56409431 | METAGENOMIC | PCR | OXFORD_NANOPORE | 51519N.fastq.gz | gastrectomy | 2015 | Chile: Santiago | 33.4489 S 70.6693 W |
| 2 | 51519T | SRR37550728 | SRP682544 | PRJNA1434549 | SAMN56409432 | METAGENOMIC | PCR | OXFORD_NANOPORE | 51519T.fastq.gz | gastrectomy | 2015 | Chile: Santiago | 33.4489 S 70.6693 W |
| 3 | 51525N | SRR37550687 | SRP682544 | PRJNA1434549 | SAMN56409433 | METAGENOMIC | PCR | OXFORD_NANOPORE | 51525N.fastq.gz | gastrectomy | 2015 | Chile: Santiago | 33.4489 S 70.6693 W |
| 4 | 51525T | SRR37550676 | SRP682544 | PRJNA1434549 | SAMN56409434 | METAGENOMIC | PCR | OXFORD_NANOPORE | 51525T.fastq.gz | gastrectomy | 2015 | Chile: Santiago | 33.4489 S 70.6693 W |
| 5 | 51631N | SRR37550631 | SRP682544 | PRJNA1434549 | SAMN56409435 | METAGENOMIC | PCR | OXFORD_NANOPORE | 51631N.fastq.gz | gastrectomy | 2016 | Chile: Santiago | 33.4489 S 70.6693 W |
| 6 | 51631T | SRR37550620 | SRP682544 | PRJNA1434549 | SAMN56409436 | METAGENOMIC | PCR | OXFORD_NANOPORE | 51631T.fastq.gz | gastrectomy | 2016 | Chile: Santiago | 33.4489 S 70.6693 W |
| 7 | 51637N | SRR37550609 | SRP682544 | PRJNA1434549 | SAMN56409437 | METAGENOMIC | PCR | OXFORD_NANOPORE | 51637N.fastq.gz | gastrectomy | 2016 | Chile: Santiago | 33.4489 S 70.6693 W |
| 8 | 51637T | SRR37550598 | SRP682544 | PRJNA1434549 | SAMN56409438 | METAGENOMIC | PCR | OXFORD_NANOPORE | 51637T.fastq.gz | gastrectomy | 2016 | Chile: Santiago | 33.4489 S 70.6693 W |
| 9 | 51638N | SRR37550587 | SRP682544 | PRJNA1434549 | SAMN56409439 | METAGENOMIC | PCR | OXFORD_NANOPORE | 51638N.fastq.gz | gastrectomy | 2016 | Chile: Santiago | 33.4489 S 70.6693 W |
| 10 | 51638T | SRR37550576 | SRP682544 | PRJNA1434549 | SAMN56409440 | METAGENOMIC | PCR | OXFORD_NANOPORE | 51638T.fastq.gz | gastrectomy | 2016 | Chile: Santiago | 33.4489 S 70.6693 W |
| 11 | 51646N | SRR37550727 | SRP682544 | PRJNA1434549 | SAMN56409441 | METAGENOMIC | PCR | OXFORD_NANOPORE | 51646N.fastq.gz | gastrectomy | 2016 | Chile: Santiago | 33.4489 S 70.6693 W |
| 12 | 51646T | SRR37550716 | SRP682544 | PRJNA1434549 | SAMN56409442 | METAGENOMIC | PCR | OXFORD_NANOPORE | 51646T.fastq.gz | gastrectomy | 2016 | Chile: Santiago | 33.4489 S 70.6693 W |
| 13 | 51655N | SRR37550705 | SRP682544 | PRJNA1434549 | SAMN56409443 | METAGENOMIC | PCR | OXFORD_NANOPORE | 51655N.fastq.gz | gastrectomy | 2016 | Chile: Santiago | 33.4489 S 70.6693 W |
| 14 | 51655T | SRR37550662 | SRP682544 | PRJNA1434549 | SAMN56409444 | METAGENOMIC | PCR | OXFORD_NANOPORE | 51655T.fastq.gz | gastrectomy | 2016 | Chile: Santiago | 33.4489 S 70.6693 W |
| 15 | 51659N | SRR37550651 | SRP682544 | PRJNA1434549 | SAMN56409445 | METAGENOMIC | PCR | OXFORD_NANOPORE | 51659N.fastq.gz | gastrectomy | 2016 | Chile: Santiago | 33.4489 S 70.6693 W |
| 16 | 51659T | SRR37550640 | SRP682544 | PRJNA1434549 | SAMN56409446 | METAGENOMIC | PCR | OXFORD_NANOPORE | 51659T.fastq.gz | gastrectomy | 2016 | Chile: Santiago | 33.4489 S 70.6693 W |
| 17 | 51660N | SRR37550693 | SRP682544 | PRJNA1434549 | SAMN56409447 | METAGENOMIC | PCR | OXFORD_NANOPORE | 51660N.fastq.gz | gastrectomy | 2016 | Chile: Santiago | 33.4489 S 70.6693 W |
| 18 | 51660T | SRR37550690 | SRP682544 | PRJNA1434549 | SAMN56409448 | METAGENOMIC | PCR | OXFORD_NANOPORE | 51660T.fastq.gz | gastrectomy | 2016 | Chile: Santiago | 33.4489 S 70.6693 W |
| 19 | 51661N | SRR37550689 | SRP682544 | PRJNA1434549 | SAMN56409449 | METAGENOMIC | PCR | OXFORD_NANOPORE | 51661N.fastq.gz | gastrectomy | 2016 | Chile: Santiago | 33.4489 S 70.6693 W |
| 20 | 51661T | SRR37550688 | SRP682544 | PRJNA1434549 | SAMN56409450 | METAGENOMIC | PCR | OXFORD_NANOPORE | 51661T.fastq.gz | gastrectomy | 2016 | Chile: Santiago | 33.4489 S 70.6693 W |
| 21 | 51712N | SRR37550686 | SRP682544 | PRJNA1434549 | SAMN56409451 | METAGENOMIC | PCR | OXFORD_NANOPORE | 51712N.fastq.gz | gastrectomy | 2017 | Chile: Santiago | 33.4489 S 70.6693 W |
| 22 | 51712T | SRR37550685 | SRP682544 | PRJNA1434549 | SAMN56409452 | METAGENOMIC | PCR | OXFORD_NANOPORE | 51712T.fastq.gz | gastrectomy | 2017 | Chile: Santiago | 33.4489 S 70.6693 W |
| 23 | 5171N | SRR37550684 | SRP682544 | PRJNA1434549 | SAMN56409453 | METAGENOMIC | PCR | OXFORD_NANOPORE | 5171N.fastq.gz | gastrectomy | 2017 | Chile: Santiago | 33.4489 S 70.6693 W |
| 24 | 5171T | SRR37550683 | SRP682544 | PRJNA1434549 | SAMN56409454 | METAGENOMIC | PCR | OXFORD_NANOPORE | 5171T.fastq.gz | gastrectomy | 2017 | Chile: Santiago | 33.4489 S 70.6693 W |
| 25 | 5172N | SRR37550682 | SRP682544 | PRJNA1434549 | SAMN56409455 | METAGENOMIC | PCR | OXFORD_NANOPORE | 5172N.fastq.gz | gastrectomy | 2017 | Chile: Santiago | 33.4489 S 70.6693 W |
| 26 | 5172T | SRR37550681 | SRP682544 | PRJNA1434549 | SAMN56409456 | METAGENOMIC | PCR | OXFORD_NANOPORE | 5172T.fastq.gz | gastrectomy | 2017 | Chile: Santiago | 33.4489 S 70.6693 W |
| 27 | 5173N | SRR37550680 | SRP682544 | PRJNA1434549 | SAMN56409457 | METAGENOMIC | PCR | OXFORD_NANOPORE | 5173N.fastq.gz | gastrectomy | 2017 | Chile: Santiago | 33.4489 S 70.6693 W |
| 28 | 5173T | SRR37550679 | SRP682544 | PRJNA1434549 | SAMN56409458 | METAGENOMIC | PCR | OXFORD_NANOPORE | 5173T.fastq.gz | gastrectomy | 2017 | Chile: Santiago | 33.4489 S 70.6693 W |
| 29 | 5174N | SRR37550678 | SRP682544 | PRJNA1434549 | SAMN56409459 | METAGENOMIC | PCR | OXFORD_NANOPORE | 5174N.fastq.gz | gastrectomy | 2017 | Chile: Santiago | 33.4489 S 70.6693 W |
| 30 | 5174T | SRR37550677 | SRP682544 | PRJNA1434549 | SAMN56409460 | METAGENOMIC | PCR | OXFORD_NANOPORE | 5174T.fastq.gz | gastrectomy | 2017 | Chile: Santiago | 33.4489 S 70.6693 W |
| 31 | 51768N | SRR37550675 | SRP682544 | PRJNA1434549 | SAMN56409461 | METAGENOMIC | PCR | OXFORD_NANOPORE | 51768N.fastq.gz | gastrectomy | 2017 | Chile: Santiago | 33.4489 S 70.6693 W |
| 32 | 51768T | SRR37550674 | SRP682544 | PRJNA1434549 | SAMN56409462 | METAGENOMIC | PCR | OXFORD_NANOPORE | 51768T.fastq.gz | gastrectomy | 2017 | Chile: Santiago | 33.4489 S 70.6693 W |
| 33 | 51771T | SRR37550673 | SRP682544 | PRJNA1434549 | SAMN56409463 | METAGENOMIC | PCR | OXFORD_NANOPORE | 51771T.fastq.gz | gastrectomy | 2017 | Chile: Santiago | 33.4489 S 70.6693 W |
| 34 | 5177N | SRR37550672 | SRP682544 | PRJNA1434549 | SAMN56409464 | METAGENOMIC | PCR | OXFORD_NANOPORE | 5177N.fastq.gz | gastrectomy | 2017 | Chile: Santiago | 33.4489 S 70.6693 W |
| 35 | 5177T | SRR37550671 | SRP682544 | PRJNA1434549 | SAMN56409465 | METAGENOMIC | PCR | OXFORD_NANOPORE | 5177T.fastq.gz | gastrectomy | 2017 | Chile: Santiago | 33.4489 S 70.6693 W |
| 36 | 51873N | SRR37550670 | SRP682544 | PRJNA1434549 | SAMN56409466 | METAGENOMIC | PCR | OXFORD_NANOPORE | 51873N.fastq.gz | gastrectomy | 2018 | Chile: Santiago | 33.4489 S 70.6693 W |
| 37 | 51873T | SRR37550669 | SRP682544 | PRJNA1434549 | SAMN56409467 | METAGENOMIC | PCR | OXFORD_NANOPORE | 51873T.fastq.gz | gastrectomy | 2018 | Chile: Santiago | 33.4489 S 70.6693 W |
| 38 | 51874N | SRR37550668 | SRP682544 | PRJNA1434549 | SAMN56409468 | METAGENOMIC | PCR | OXFORD_NANOPORE | 51874N.fastq.gz | gastrectomy | 2018 | Chile: Santiago | 33.4489 S 70.6693 W |
| 39 | 51874T | SRR37550667 | SRP682544 | PRJNA1434549 | SAMN56409469 | METAGENOMIC | PCR | OXFORD_NANOPORE | 51874T.fastq.gz | gastrectomy | 2018 | Chile: Santiago | 33.4489 S 70.6693 W |
| 40 | 51989N | SRR37550666 | SRP682544 | PRJNA1434549 | SAMN56409470 | METAGENOMIC | PCR | OXFORD_NANOPORE | 51989N.fastq.gz | gastrectomy | 2019 | Chile: Santiago | 33.4489 S 70.6693 W |
| 41 | 51989T | SRR37550630 | SRP682544 | PRJNA1434549 | SAMN56409471 | METAGENOMIC | PCR | OXFORD_NANOPORE | 51989T.fastq.gz | gastrectomy | 2019 | Chile: Santiago | 33.4489 S 70.6693 W |
| 42 | 51991N | SRR37550629 | SRP682544 | PRJNA1434549 | SAMN56409472 | METAGENOMIC | PCR | OXFORD_NANOPORE | 51991N.fastq.gz | gastrectomy | 2019 | Chile: Santiago | 33.4489 S 70.6693 W |
| 43 | 51991T | SRR37550628 | SRP682544 | PRJNA1434549 | SAMN56409473 | METAGENOMIC | PCR | OXFORD_NANOPORE | 51991T.fastq.gz | gastrectomy | 2019 | Chile: Santiago | 33.4489 S 70.6693 W |
| 44 | 51992N | SRR37550627 | SRP682544 | PRJNA1434549 | SAMN56409474 | METAGENOMIC | PCR | OXFORD_NANOPORE | 51992N.fastq.gz | gastrectomy | 2019 | Chile: Santiago | 33.4489 S 70.6693 W |
| 45 | 51992T | SRR37550626 | SRP682544 | PRJNA1434549 | SAMN56409475 | METAGENOMIC | PCR | OXFORD_NANOPORE | 51992T.fastq.gz | gastrectomy | 2019 | Chile: Santiago | 33.4489 S 70.6693 W |
| 46 | 51993N | SRR37550625 | SRP682544 | PRJNA1434549 | SAMN56409476 | METAGENOMIC | PCR | OXFORD_NANOPORE | 51993N.fastq.gz | gastrectomy | 2019 | Chile: Santiago | 33.4489 S 70.6693 W |
| 47 | 520103N | SRR37550624 | SRP682544 | PRJNA1434549 | SAMN56409477 | METAGENOMIC | PCR | OXFORD_NANOPORE | 520103N.fastq.gz | gastrectomy | 2020 | Chile: Santiago | 33.4489 S 70.6693 W |
| 48 | 520103T | SRR37550623 | SRP682544 | PRJNA1434549 | SAMN56409478 | METAGENOMIC | PCR | OXFORD_NANOPORE | 520103T.fastq.gz | gastrectomy | 2020 | Chile: Santiago | 33.4489 S 70.6693 W |
| 49 | 520104N | SRR37550622 | SRP682544 | PRJNA1434549 | SAMN56409479 | METAGENOMIC | PCR | OXFORD_NANOPORE | 520104N.fastq.gz | gastrectomy | 2020 | Chile: Santiago | 33.4489 S 70.6693 W |
| 50 | 520104T | SRR37550621 | SRP682544 | PRJNA1434549 | SAMN56409480 | METAGENOMIC | PCR | OXFORD_NANOPORE | 520104T.fastq.gz | gastrectomy | 2020 | Chile: Santiago | 33.4489 S 70.6693 W |
| 51 | 52110N | SRR37550619 | SRP682544 | PRJNA1434549 | SAMN56409481 | METAGENOMIC | PCR | OXFORD_NANOPORE | 52110N.fastq.gz | gastrectomy | 2021 | Chile: Santiago | 33.4489 S 70.6693 W |
| 52 | 52110T | SRR37550618 | SRP682544 | PRJNA1434549 | SAMN56409482 | METAGENOMIC | PCR | OXFORD_NANOPORE | 52110T.fastq.gz | gastrectomy | 2021 | Chile: Santiago | 33.4489 S 70.6693 W |
| 53 | 5213N | SRR37550617 | SRP682544 | PRJNA1434549 | SAMN56409483 | METAGENOMIC | PCR | OXFORD_NANOPORE | 5213N.fastq.gz | gastrectomy | 2021 | Chile: Santiago | 33.4489 S 70.6693 W |
| 54 | 5213T | SRR37550616 | SRP682544 | PRJNA1434549 | SAMN56409484 | METAGENOMIC | PCR | OXFORD_NANOPORE | 5213T.fastq.gz | gastrectomy | 2021 | Chile: Santiago | 33.4489 S 70.6693 W |
| 55 | 5214N | SRR37550615 | SRP682544 | PRJNA1434549 | SAMN56409485 | METAGENOMIC | PCR | OXFORD_NANOPORE | 5214N.fastq.gz | gastrectomy | 2021 | Chile: Santiago | 33.4489 S 70.6693 W |
| 56 | 5214T | SRR37550614 | SRP682544 | PRJNA1434549 | SAMN56409486 | METAGENOMIC | PCR | OXFORD_NANOPORE | 5214T.fastq.gz | gastrectomy | 2021 | Chile: Santiago | 33.4489 S 70.6693 W |
| 57 | 5219N | SRR37550613 | SRP682544 | PRJNA1434549 | SAMN56409487 | METAGENOMIC | PCR | OXFORD_NANOPORE | 5219N.fastq.gz | gastrectomy | 2021 | Chile: Santiago | 33.4489 S 70.6693 W |
| 58 | 5219T | SRR37550612 | SRP682544 | PRJNA1434549 | SAMN56409488 | METAGENOMIC | PCR | OXFORD_NANOPORE | 5219T.fastq.gz | gastrectomy | 2021 | Chile: Santiago | 33.4489 S 70.6693 W |
| 59 | 5220006N | SRR37550611 | SRP682544 | PRJNA1434549 | SAMN56409489 | METAGENOMIC | PCR | OXFORD_NANOPORE | 5220006N.fastq.gz | gastrectomy | 2022 | Chile: Santiago | 33.4489 S 70.6693 W |
| 60 | 5220006T | SRR37550610 | SRP682544 | PRJNA1434549 | SAMN56409490 | METAGENOMIC | PCR | OXFORD_NANOPORE | 5220006T.fastq.gz | gastrectomy | 2022 | Chile: Santiago | 33.4489 S 70.6693 W |
| 61 | GC0001N | SRR37550608 | SRP682544 | PRJNA1434549 | SAMN56409491 | METAGENOMIC | PCR | OXFORD_NANOPORE | GC0001N.fastq.gz | gastrectomy | 2018 | Chile: Punta Arenas | 53.1638 S 70.9171 W |
| 62 | GC0001T | SRR37550607 | SRP682544 | PRJNA1434549 | SAMN56409492 | METAGENOMIC | PCR | OXFORD_NANOPORE | GC0001T.fastq.gz | gastrectomy | 2018 | Chile: Punta Arenas | 53.1638 S 70.9171 W |
| 63 | GC0026A | SRR37550606 | SRP682544 | PRJNA1434549 | SAMN56409493 | METAGENOMIC | PCR | OXFORD_NANOPORE | GC0026A.fastq.gz | gastric mucosa biopsy | 2018 | Chile: Punta Arenas | 53.1638 S 70.9171 W |
| 64 | GC0029A | SRR37550605 | SRP682544 | PRJNA1434549 | SAMN56409494 | METAGENOMIC | PCR | OXFORD_NANOPORE | GC0029A.fastq.gz | gastric mucosa biopsy | 2018 | Chile: Punta Arenas | 53.1638 S 70.9171 W |
| 65 | GC0029C | SRR37550604 | SRP682544 | PRJNA1434549 | SAMN56409495 | METAGENOMIC | PCR | OXFORD_NANOPORE | GC0029C.fastq.gz | gastric mucosa biopsy | 2018 | Chile: Punta Arenas | 53.1638 S 70.9171 W |
| 66 | GC0041A | SRR37550603 | SRP682544 | PRJNA1434549 | SAMN56409496 | METAGENOMIC | PCR | OXFORD_NANOPORE | GC0041A.fastq.gz | gastric mucosa biopsy | 2018 | Chile: Punta Arenas | 53.1638 S 70.9171 W |
| 67 | GC0041C | SRR37550602 | SRP682544 | PRJNA1434549 | SAMN56409497 | METAGENOMIC | PCR | OXFORD_NANOPORE | GC0041C.fastq.gz | gastric mucosa biopsy | 2018 | Chile: Punta Arenas | 53.1638 S 70.9171 W |
| 68 | GC0046A | SRR37550601 | SRP682544 | PRJNA1434549 | SAMN56409498 | METAGENOMIC | PCR | OXFORD_NANOPORE | GC0046A.fastq.gz | gastric mucosa biopsy | 2018 | Chile: Punta Arenas | 53.1638 S 70.9171 W |
| 69 | GC0046C | SRR37550600 | SRP682544 | PRJNA1434549 | SAMN56409499 | METAGENOMIC | PCR | OXFORD_NANOPORE | GC0046C.fastq.gz | gastric mucosa biopsy | 2018 | Chile: Punta Arenas | 53.1638 S 70.9171 W |
| 70 | GC0055A | SRR37550599 | SRP682544 | PRJNA1434549 | SAMN56409500 | METAGENOMIC | PCR | OXFORD_NANOPORE | GC0055A.fastq.gz | gastric mucosa biopsy | 2018 | Chile: Punta Arenas | 53.1638 S 70.9171 W |
| 71 | GC0055C | SRR37550597 | SRP682544 | PRJNA1434549 | SAMN56409501 | METAGENOMIC | PCR | OXFORD_NANOPORE | GC0055C.fastq.gz | gastric mucosa biopsy | 2018 | Chile: Punta Arenas | 53.1638 S 70.9171 W |
| 72 | GC0095A | SRR37550596 | SRP682544 | PRJNA1434549 | SAMN56409502 | METAGENOMIC | PCR | OXFORD_NANOPORE | GC0095A.fastq.gz | gastric mucosa biopsy | 2019 | Chile: Punta Arenas | 53.1638 S 70.9171 W |
| 73 | GC0095C | SRR37550595 | SRP682544 | PRJNA1434549 | SAMN56409503 | METAGENOMIC | PCR | OXFORD_NANOPORE | GC0095C.fastq.gz | gastric mucosa biopsy | 2019 | Chile: Punta Arenas | 53.1638 S 70.9171 W |
| 74 | GC0116A | SRR37550594 | SRP682544 | PRJNA1434549 | SAMN56409504 | METAGENOMIC | PCR | OXFORD_NANOPORE | GC0116A.fastq.gz | gastric mucosa biopsy | 2019 | Chile: Punta Arenas | 53.1638 S 70.9171 W |
| 75 | GC0116C | SRR37550593 | SRP682544 | PRJNA1434549 | SAMN56409505 | METAGENOMIC | PCR | OXFORD_NANOPORE | GC0116C.fastq.gz | gastric mucosa biopsy | 2019 | Chile: Punta Arenas | 53.1638 S 70.9171 W |
| 76 | GC0140A | SRR37550592 | SRP682544 | PRJNA1434549 | SAMN56409506 | METAGENOMIC | PCR | OXFORD_NANOPORE | GC0140A.fastq.gz | gastric mucosa biopsy | 2019 | Chile: Punta Arenas | 53.1638 S 70.9171 W |
| 77 | GC0140C | SRR37550591 | SRP682544 | PRJNA1434549 | SAMN56409507 | METAGENOMIC | PCR | OXFORD_NANOPORE | GC0140C.fastq.gz | gastric mucosa biopsy | 2019 | Chile: Punta Arenas | 53.1638 S 70.9171 W |
| 78 | GC0156A | SRR37550590 | SRP682544 | PRJNA1434549 | SAMN56409508 | METAGENOMIC | PCR | OXFORD_NANOPORE | GC0156A.fastq.gz | gastric mucosa biopsy | 2019 | Chile: Punta Arenas | 53.1638 S 70.9171 W |
| 79 | GC0156C | SRR37550589 | SRP682544 | PRJNA1434549 | SAMN56409509 | METAGENOMIC | PCR | OXFORD_NANOPORE | GC0156C.fastq.gz | gastric mucosa biopsy | 2019 | Chile: Punta Arenas | 53.1638 S 70.9171 W |
| 80 | GC0157A | SRR37550588 | SRP682544 | PRJNA1434549 | SAMN56409510 | METAGENOMIC | PCR | OXFORD_NANOPORE | GC0157A.fastq.gz | gastric mucosa biopsy | 2019 | Chile: Punta Arenas | 53.1638 S 70.9171 W |
| 81 | GC0157C | SRR37550586 | SRP682544 | PRJNA1434549 | SAMN56409511 | METAGENOMIC | PCR | OXFORD_NANOPORE | GC0157C.fastq.gz | gastric mucosa biopsy | 2019 | Chile: Punta Arenas | 53.1638 S 70.9171 W |
| 82 | GC0159A | SRR37550585 | SRP682544 | PRJNA1434549 | SAMN56409512 | METAGENOMIC | PCR | OXFORD_NANOPORE | GC0159A.fastq.gz | gastric mucosa biopsy | 2019 | Chile: Punta Arenas | 53.1638 S 70.9171 W |
| 83 | GC0159C | SRR37550584 | SRP682544 | PRJNA1434549 | SAMN56409513 | METAGENOMIC | PCR | OXFORD_NANOPORE | GC0159C.fastq.gz | gastric mucosa biopsy | 2019 | Chile: Punta Arenas | 53.1638 S 70.9171 W |
| 84 | GC0163A | SRR37550583 | SRP682544 | PRJNA1434549 | SAMN56409514 | METAGENOMIC | PCR | OXFORD_NANOPORE | GC0163A.fastq.gz | gastric mucosa biopsy | 2019 | Chile: Punta Arenas | 53.1638 S 70.9171 W |
| 85 | GC0163C | SRR37550582 | SRP682544 | PRJNA1434549 | SAMN56409515 | METAGENOMIC | PCR | OXFORD_NANOPORE | GC0163C.fastq.gz | gastric mucosa biopsy | 2019 | Chile: Punta Arenas | 53.1638 S 70.9171 W |
| 86 | GC0165N | SRR37550581 | SRP682544 | PRJNA1434549 | SAMN56409516 | METAGENOMIC | PCR | OXFORD_NANOPORE | GC0165N.fastq.gz | gastrectomy | 2019 | Chile: Punta Arenas | 53.1638 S 70.9171 W |
| 87 | GC0165T | SRR37550580 | SRP682544 | PRJNA1434549 | SAMN56409517 | METAGENOMIC | PCR | OXFORD_NANOPORE | GC0165T.fastq.gz | gastrectomy | 2019 | Chile: Punta Arenas | 53.1638 S 70.9171 W |
| 88 | GC0166A | SRR37550579 | SRP682544 | PRJNA1434549 | SAMN56409518 | METAGENOMIC | PCR | OXFORD_NANOPORE | GC0166A.fastq.gz | gastric mucosa biopsy | 2019 | Chile: Punta Arenas | 53.1638 S 70.9171 W |
| 89 | GC0166C | SRR37550578 | SRP682544 | PRJNA1434549 | SAMN56409519 | METAGENOMIC | PCR | OXFORD_NANOPORE | GC0166C.fastq.gz | gastric mucosa biopsy | 2019 | Chile: Punta Arenas | 53.1638 S 70.9171 W |
| 90 | GC0167A | SRR37550577 | SRP682544 | PRJNA1434549 | SAMN56409520 | METAGENOMIC | PCR | OXFORD_NANOPORE | GC0167A.fastq.gz | gastric mucosa biopsy | 2019 | Chile: Punta Arenas | 53.1638 S 70.9171 W |
| 91 | GC0173A | SRR37550575 | SRP682544 | PRJNA1434549 | SAMN56409521 | METAGENOMIC | PCR | OXFORD_NANOPORE | GC0173A.fastq.gz | gastric mucosa biopsy | 2019 | Chile: Punta Arenas | 53.1638 S 70.9171 W |
| 92 | GC0173C | SRR37550574 | SRP682544 | PRJNA1434549 | SAMN56409522 | METAGENOMIC | PCR | OXFORD_NANOPORE | GC0173C.fastq.gz | gastric mucosa biopsy | 2019 | Chile: Punta Arenas | 53.1638 S 70.9171 W |
| 93 | GC0194A | SRR37550573 | SRP682544 | PRJNA1434549 | SAMN56409523 | METAGENOMIC | PCR | OXFORD_NANOPORE | GC0194A.fastq.gz | gastric mucosa biopsy | 2019 | Chile: Punta Arenas | 53.1638 S 70.9171 W |
| 94 | GC0194C | SRR37550572 | SRP682544 | PRJNA1434549 | SAMN56409524 | METAGENOMIC | PCR | OXFORD_NANOPORE | GC0194C.fastq.gz | gastric mucosa biopsy | 2019 | Chile: Punta Arenas | 53.1638 S 70.9171 W |
| 95 | GC0206A | SRR37550571 | SRP682544 | PRJNA1434549 | SAMN56409525 | METAGENOMIC | PCR | OXFORD_NANOPORE | GC0206A.fastq.gz | gastric mucosa biopsy | 2019 | Chile: Punta Arenas | 53.1638 S 70.9171 W |
| 96 | GC0206C | SRR37550570 | SRP682544 | PRJNA1434549 | SAMN56409526 | METAGENOMIC | PCR | OXFORD_NANOPORE | GC0206C.fastq.gz | gastric mucosa biopsy | 2019 | Chile: Punta Arenas | 53.1638 S 70.9171 W |
| 97 | GC0218A | SRR37550569 | SRP682544 | PRJNA1434549 | SAMN56409527 | METAGENOMIC | PCR | OXFORD_NANOPORE | GC0218A.fastq.gz | gastric mucosa biopsy | 2019 | Chile: Punta Arenas | 53.1638 S 70.9171 W |
| 98 | GC0218C | SRR37550568 | SRP682544 | PRJNA1434549 | SAMN56409528 | METAGENOMIC | PCR | OXFORD_NANOPORE | GC0218C.fastq.gz | gastric mucosa biopsy | 2019 | Chile: Punta Arenas | 53.1638 S 70.9171 W |
| 99 | GC0227A | SRR37550633 | SRP682544 | PRJNA1434549 | SAMN56409529 | METAGENOMIC | PCR | OXFORD_NANOPORE | GC0227A.fastq.gz | gastric mucosa biopsy | 2019 | Chile: Punta Arenas | 53.1638 S 70.9171 W |
| 100 | GC0227C | SRR37550632 | SRP682544 | PRJNA1434549 | SAMN56409530 | METAGENOMIC | PCR | OXFORD_NANOPORE | GC0227C.fastq.gz | gastric mucosa biopsy | 2019 | Chile: Punta Arenas | 53.1638 S 70.9171 W |
| 101 | GC0232A | SRR37550726 | SRP682544 | PRJNA1434549 | SAMN56409531 | METAGENOMIC | PCR | OXFORD_NANOPORE | GC0232A.fastq.gz | gastric mucosa biopsy | 2019 | Chile: Punta Arenas | 53.1638 S 70.9171 W |
| 102 | GC0232C | SRR37550725 | SRP682544 | PRJNA1434549 | SAMN56409532 | METAGENOMIC | PCR | OXFORD_NANOPORE | GC0232C.fastq.gz | gastric mucosa biopsy | 2019 | Chile: Punta Arenas | 53.1638 S 70.9171 W |
| 103 | GC0234A | SRR37550724 | SRP682544 | PRJNA1434549 | SAMN56409533 | METAGENOMIC | PCR | OXFORD_NANOPORE | GC0234A.fastq.gz | gastric mucosa biopsy | 2019 | Chile: Punta Arenas | 53.1638 S 70.9171 W |
| 104 | GC0234C | SRR37550723 | SRP682544 | PRJNA1434549 | SAMN56409534 | METAGENOMIC | PCR | OXFORD_NANOPORE | GC0234C.fastq.gz | gastric mucosa biopsy | 2019 | Chile: Punta Arenas | 53.1638 S 70.9171 W |
| 105 | GC0246A | SRR37550722 | SRP682544 | PRJNA1434549 | SAMN56409535 | METAGENOMIC | PCR | OXFORD_NANOPORE | GC0246A.fastq.gz | gastric mucosa biopsy | 2019 | Chile: Punta Arenas | 53.1638 S 70.9171 W |
| 106 | GC0246C | SRR37550721 | SRP682544 | PRJNA1434549 | SAMN56409536 | METAGENOMIC | PCR | OXFORD_NANOPORE | GC0246C.fastq.gz | gastric mucosa biopsy | 2019 | Chile: Punta Arenas | 53.1638 S 70.9171 W |
| 107 | GC0250A | SRR37550720 | SRP682544 | PRJNA1434549 | SAMN56409537 | METAGENOMIC | PCR | OXFORD_NANOPORE | GC0250A.fastq.gz | gastric mucosa biopsy | 2019 | Chile: Punta Arenas | 53.1638 S 70.9171 W |
| 108 | GC0250C | SRR37550719 | SRP682544 | PRJNA1434549 | SAMN56409538 | METAGENOMIC | PCR | OXFORD_NANOPORE | GC0250C.fastq.gz | gastric mucosa biopsy | 2019 | Chile: Punta Arenas | 53.1638 S 70.9171 W |
| 109 | GC0256N | SRR37550718 | SRP682544 | PRJNA1434549 | SAMN56409539 | METAGENOMIC | PCR | OXFORD_NANOPORE | GC0256N.fastq.gz | gastrectomy | 2019 | Chile: Punta Arenas | 53.1638 S 70.9171 W |
| 110 | GC0256T | SRR37550717 | SRP682544 | PRJNA1434549 | SAMN56409540 | METAGENOMIC | PCR | OXFORD_NANOPORE | GC0256T.fastq.gz | gastrectomy | 2019 | Chile: Punta Arenas | 53.1638 S 70.9171 W |
| 111 | GC0271A | SRR37550715 | SRP682544 | PRJNA1434549 | SAMN56409541 | METAGENOMIC | PCR | OXFORD_NANOPORE | GC0271A.fastq.gz | gastric mucosa biopsy | 2019 | Chile: Punta Arenas | 53.1638 S 70.9171 W |
| 112 | GC0271C | SRR37550714 | SRP682544 | PRJNA1434549 | SAMN56409542 | METAGENOMIC | PCR | OXFORD_NANOPORE | GC0271C.fastq.gz | gastric mucosa biopsy | 2019 | Chile: Punta Arenas | 53.1638 S 70.9171 W |
| 113 | GC0272A | SRR37550713 | SRP682544 | PRJNA1434549 | SAMN56409543 | METAGENOMIC | PCR | OXFORD_NANOPORE | GC0272A.fastq.gz | gastric mucosa biopsy | 2019 | Chile: Punta Arenas | 53.1638 S 70.9171 W |
| 114 | GC0272C | SRR37550712 | SRP682544 | PRJNA1434549 | SAMN56409544 | METAGENOMIC | PCR | OXFORD_NANOPORE | GC0272C.fastq.gz | gastric mucosa biopsy | 2019 | Chile: Punta Arenas | 53.1638 S 70.9171 W |
| 115 | GC0280A | SRR37550711 | SRP682544 | PRJNA1434549 | SAMN56409545 | METAGENOMIC | PCR | OXFORD_NANOPORE | GC0280A.fastq.gz | gastric mucosa biopsy | 2019 | Chile: Punta Arenas | 53.1638 S 70.9171 W |
| 116 | GC0280C | SRR37550710 | SRP682544 | PRJNA1434549 | SAMN56409546 | METAGENOMIC | PCR | OXFORD_NANOPORE | GC0280C.fastq.gz | gastric mucosa biopsy | 2019 | Chile: Punta Arenas | 53.1638 S 70.9171 W |
| 117 | GC0281A | SRR37550709 | SRP682544 | PRJNA1434549 | SAMN56409547 | METAGENOMIC | PCR | OXFORD_NANOPORE | GC0281A.fastq.gz | gastric mucosa biopsy | 2019 | Chile: Punta Arenas | 53.1638 S 70.9171 W |
| 118 | GC0281C | SRR37550708 | SRP682544 | PRJNA1434549 | SAMN56409548 | METAGENOMIC | PCR | OXFORD_NANOPORE | GC0281C.fastq.gz | gastric mucosa biopsy | 2019 | Chile: Punta Arenas | 53.1638 S 70.9171 W |
| 119 | GC0287A | SRR37550707 | SRP682544 | PRJNA1434549 | SAMN56409549 | METAGENOMIC | PCR | OXFORD_NANOPORE | GC0287A.fastq.gz | gastric mucosa biopsy | 2019 | Chile: Punta Arenas | 53.1638 S 70.9171 W |
| 120 | GC0287C | SRR37550706 | SRP682544 | PRJNA1434549 | SAMN56409550 | METAGENOMIC | PCR | OXFORD_NANOPORE | GC0287C.fastq.gz | gastric mucosa biopsy | 2019 | Chile: Punta Arenas | 53.1638 S 70.9171 W |
| 121 | GC0300A | SRR37550704 | SRP682544 | PRJNA1434549 | SAMN56409551 | METAGENOMIC | PCR | OXFORD_NANOPORE | GC0300A.fastq.gz | gastric mucosa biopsy | 2019 | Chile: Punta Arenas | 53.1638 S 70.9171 W |
| 122 | GC0300C | SRR37550703 | SRP682544 | PRJNA1434549 | SAMN56409552 | METAGENOMIC | PCR | OXFORD_NANOPORE | GC0300C.fastq.gz | gastric mucosa biopsy | 2019 | Chile: Punta Arenas | 53.1638 S 70.9171 W |
| 123 | GC0308N | SRR37550702 | SRP682544 | PRJNA1434549 | SAMN56409553 | METAGENOMIC | PCR | OXFORD_NANOPORE | GC0308N.fastq.gz | gastrectomy | 2019 | Chile: Punta Arenas | 53.1638 S 70.9171 W |
| 124 | GC0308T | SRR37550701 | SRP682544 | PRJNA1434549 | SAMN56409554 | METAGENOMIC | PCR | OXFORD_NANOPORE | GC0308T.fastq.gz | gastrectomy | 2019 | Chile: Punta Arenas | 53.1638 S 70.9171 W |
| 125 | GC0312A | SRR37550700 | SRP682544 | PRJNA1434549 | SAMN56409555 | METAGENOMIC | PCR | OXFORD_NANOPORE | GC0312A.fastq.gz | gastric mucosa biopsy | 2019 | Chile: Punta Arenas | 53.1638 S 70.9171 W |
| 126 | GC0312C | SRR37550699 | SRP682544 | PRJNA1434549 | SAMN56409556 | METAGENOMIC | PCR | OXFORD_NANOPORE | GC0312C.fastq.gz | gastric mucosa biopsy | 2019 | Chile: Punta Arenas | 53.1638 S 70.9171 W |
| 127 | GC0323A | SRR37550698 | SRP682544 | PRJNA1434549 | SAMN56409557 | METAGENOMIC | PCR | OXFORD_NANOPORE | GC0323A.fastq.gz | gastric mucosa biopsy | 2019 | Chile: Punta Arenas | 53.1638 S 70.9171 W |
| 128 | GC0323C | SRR37550665 | SRP682544 | PRJNA1434549 | SAMN56409558 | METAGENOMIC | PCR | OXFORD_NANOPORE | GC0323C.fastq.gz | gastric mucosa biopsy | 2019 | Chile: Punta Arenas | 53.1638 S 70.9171 W |
| 129 | GC0332A | SRR37550664 | SRP682544 | PRJNA1434549 | SAMN56409559 | METAGENOMIC | PCR | OXFORD_NANOPORE | GC0332A.fastq.gz | gastric mucosa biopsy | 2019 | Chile: Punta Arenas | 53.1638 S 70.9171 W |
| 130 | GC0332C | SRR37550663 | SRP682544 | PRJNA1434549 | SAMN56409560 | METAGENOMIC | PCR | OXFORD_NANOPORE | GC0332C.fastq.gz | gastric mucosa biopsy | 2019 | Chile: Punta Arenas | 53.1638 S 70.9171 W |
| 131 | GC0340A | SRR37550661 | SRP682544 | PRJNA1434549 | SAMN56409561 | METAGENOMIC | PCR | OXFORD_NANOPORE | GC0340A.fastq.gz | gastric mucosa biopsy | 2019 | Chile: Punta Arenas | 53.1638 S 70.9171 W |
| 132 | GC0340C | SRR37550660 | SRP682544 | PRJNA1434549 | SAMN56409562 | METAGENOMIC | PCR | OXFORD_NANOPORE | GC0340C.fastq.gz | gastric mucosa biopsy | 2019 | Chile: Punta Arenas | 53.1638 S 70.9171 W |
| 133 | GC0343A | SRR37550659 | SRP682544 | PRJNA1434549 | SAMN56409563 | METAGENOMIC | PCR | OXFORD_NANOPORE | GC0343A.fastq.gz | gastric mucosa biopsy | 2019 | Chile: Punta Arenas | 53.1638 S 70.9171 W |
| 134 | GC0343C | SRR37550658 | SRP682544 | PRJNA1434549 | SAMN56409564 | METAGENOMIC | PCR | OXFORD_NANOPORE | GC0343C.fastq.gz | gastric mucosa biopsy | 2019 | Chile: Punta Arenas | 53.1638 S 70.9171 W |
| 135 | GC0355A | SRR37550657 | SRP682544 | PRJNA1434549 | SAMN56409565 | METAGENOMIC | PCR | OXFORD_NANOPORE | GC0355A.fastq.gz | gastric mucosa biopsy | 2019 | Chile: Punta Arenas | 53.1638 S 70.9171 W |
| 136 | GC0355C | SRR37550656 | SRP682544 | PRJNA1434549 | SAMN56409566 | METAGENOMIC | PCR | OXFORD_NANOPORE | GC0355C.fastq.gz | gastric mucosa biopsy | 2019 | Chile: Punta Arenas | 53.1638 S 70.9171 W |
| 137 | GC0357N | SRR37550655 | SRP682544 | PRJNA1434549 | SAMN56409567 | METAGENOMIC | PCR | OXFORD_NANOPORE | GC0357N.fastq.gz | gastrectomy | 2019 | Chile: Punta Arenas | 53.1638 S 70.9171 W |
| 138 | GC0357T | SRR37550654 | SRP682544 | PRJNA1434549 | SAMN56409568 | METAGENOMIC | PCR | OXFORD_NANOPORE | GC0357T.fastq.gz | gastrectomy | 2019 | Chile: Punta Arenas | 53.1638 S 70.9171 W |
| 139 | GC0366A | SRR37550653 | SRP682544 | PRJNA1434549 | SAMN56409569 | METAGENOMIC | PCR | OXFORD_NANOPORE | GC0366A.fastq.gz | gastric mucosa biopsy | 2019 | Chile: Punta Arenas | 53.1638 S 70.9171 W |
| 140 | GC0366C | SRR37550652 | SRP682544 | PRJNA1434549 | SAMN56409570 | METAGENOMIC | PCR | OXFORD_NANOPORE | GC0366C.fastq.gz | gastric mucosa biopsy | 2019 | Chile: Punta Arenas | 53.1638 S 70.9171 W |
| 141 | GC0366N | SRR37550650 | SRP682544 | PRJNA1434549 | SAMN56409571 | METAGENOMIC | PCR | OXFORD_NANOPORE | GC0366N.fastq.gz | gastrectomy | 2019 | Chile: Punta Arenas | 53.1638 S 70.9171 W |
| 142 | GC0366T | SRR37550649 | SRP682544 | PRJNA1434549 | SAMN56409572 | METAGENOMIC | PCR | OXFORD_NANOPORE | GC0366T.fastq.gz | gastrectomy | 2019 | Chile: Punta Arenas | 53.1638 S 70.9171 W |
| 143 | GC0373A | SRR37550648 | SRP682544 | PRJNA1434549 | SAMN56409573 | METAGENOMIC | PCR | OXFORD_NANOPORE | GC0373A.fastq.gz | gastric mucosa biopsy | 2019 | Chile: Punta Arenas | 53.1638 S 70.9171 W |
| 144 | GC0373C | SRR37550647 | SRP682544 | PRJNA1434549 | SAMN56409574 | METAGENOMIC | PCR | OXFORD_NANOPORE | GC0373C.fastq.gz | gastric mucosa biopsy | 2019 | Chile: Punta Arenas | 53.1638 S 70.9171 W |
| 145 | GC0385A | SRR37550646 | SRP682544 | PRJNA1434549 | SAMN56409575 | METAGENOMIC | PCR | OXFORD_NANOPORE | GC0385A.fastq.gz | gastric mucosa biopsy | 2019 | Chile: Punta Arenas | 53.1638 S 70.9171 W |
| 146 | GC0385C | SRR37550645 | SRP682544 | PRJNA1434549 | SAMN56409576 | METAGENOMIC | PCR | OXFORD_NANOPORE | GC0385C.fastq.gz | gastric mucosa biopsy | 2019 | Chile: Punta Arenas | 53.1638 S 70.9171 W |
| 147 | GC0431N | SRR37550644 | SRP682544 | PRJNA1434549 | SAMN56409577 | METAGENOMIC | PCR | OXFORD_NANOPORE | GC0431N.fastq.gz | gastrectomy | 2021 | Chile: Punta Arenas | 53.1638 S 70.9171 W |
| 148 | GC0431T | SRR37550643 | SRP682544 | PRJNA1434549 | SAMN56409578 | METAGENOMIC | PCR | OXFORD_NANOPORE | GC0431T.fastq.gz | gastrectomy | 2021 | Chile: Punta Arenas | 53.1638 S 70.9171 W |
| 149 | GC0448A | SRR37550642 | SRP682544 | PRJNA1434549 | SAMN56409579 | METAGENOMIC | PCR | OXFORD_NANOPORE | GC0448A.fastq.gz | gastric mucosa biopsy | 2021 | Chile: Punta Arenas | 53.1638 S 70.9171 W |
| 150 | GC0448C | SRR37550641 | SRP682544 | PRJNA1434549 | SAMN56409580 | METAGENOMIC | PCR | OXFORD_NANOPORE | GC0448C.fastq.gz | gastric mucosa biopsy | 2021 | Chile: Punta Arenas | 53.1638 S 70.9171 W |
| 151 | GC0462A | SRR37550639 | SRP682544 | PRJNA1434549 | SAMN56409581 | METAGENOMIC | PCR | OXFORD_NANOPORE | GC0462A.fastq.gz | gastric mucosa biopsy | 2021 | Chile: Punta Arenas | 53.1638 S 70.9171 W |
| 152 | GC0462C | SRR37550638 | SRP682544 | PRJNA1434549 | SAMN56409582 | METAGENOMIC | PCR | OXFORD_NANOPORE | GC0462C.fastq.gz | gastric mucosa biopsy | 2021 | Chile: Punta Arenas | 53.1638 S 70.9171 W |
| 153 | GC0466N | SRR37550637 | SRP682544 | PRJNA1434549 | SAMN56409583 | METAGENOMIC | PCR | OXFORD_NANOPORE | GC0466N.fastq.gz | gastrectomy | 2022 | Chile: Punta Arenas | 53.1638 S 70.9171 W |
| 154 | GC0466T | SRR37550636 | SRP682544 | PRJNA1434549 | SAMN56409584 | METAGENOMIC | PCR | OXFORD_NANOPORE | GC0466T.fastq.gz | gastrectomy | 2022 | Chile: Punta Arenas | 53.1638 S 70.9171 W |
| 155 | GC0470N | SRR37550635 | SRP682544 | PRJNA1434549 | SAMN56409585 | METAGENOMIC | PCR | OXFORD_NANOPORE | GC0470N.fastq.gz | gastrectomy | 2022 | Chile: Punta Arenas | 53.1638 S 70.9171 W |
| 156 | GC0470T | SRR37550634 | SRP682544 | PRJNA1434549 | SAMN56409586 | METAGENOMIC | PCR | OXFORD_NANOPORE | GC0470T.fastq.gz | gastrectomy | 2022 | Chile: Punta Arenas | 53.1638 S 70.9171 W |
| 157 | GC0487N | SRR37550697 | SRP682544 | PRJNA1434549 | SAMN56409587 | METAGENOMIC | PCR | OXFORD_NANOPORE | GC0487N.fastq.gz | gastrectomy | 2022 | Chile: Punta Arenas | 53.1638 S 70.9171 W |
| 158 | GC0487T | SRR37550696 | SRP682544 | PRJNA1434549 | SAMN56409588 | METAGENOMIC | PCR | OXFORD_NANOPORE | GC0487T.fastq.gz | gastrectomy | 2022 | Chile: Punta Arenas | 53.1638 S 70.9171 W |
| 159 | GC0546N | SRR37550695 | SRP682544 | PRJNA1434549 | SAMN56409589 | METAGENOMIC | PCR | OXFORD_NANOPORE | GC0546N.fastq.gz | gastrectomy | 2022 | Chile: Punta Arenas | 53.1638 S 70.9171 W |
| 160 | GC0546T | SRR37550694 | SRP682544 | PRJNA1434549 | SAMN56409590 | METAGENOMIC | PCR | OXFORD_NANOPORE | GC0546T.fastq.gz | gastrectomy | 2022 | Chile: Punta Arenas | 53.1638 S 70.9171 W |
| 161 | GC0558N | SRR37550692 | SRP682544 | PRJNA1434549 | SAMN56409591 | METAGENOMIC | PCR | OXFORD_NANOPORE | GC0558N.fastq.gz | gastrectomy | 2022 | Chile: Punta Arenas | 53.1638 S 70.9171 W |
| 162 | GC0558T | SRR37550691 | SRP682544 | PRJNA1434549 | SAMN56409592 | METAGENOMIC | PCR | OXFORD_NANOPORE | GC0558T.fastq.gz | gastrectomy | 2022 | Chile: Punta Arenas | 53.1638 S 70.9171 W |

*The table includes tissue identifiers, sequencing library information, SRA accession numbers, BioProject and BioSample accessions, sequencing and platform details, tissue origin, collection metadata, and processing information for all tissues included in this study. n, tissue number; tissue_name, tissue identifier; accession, SRA run accession number; study, SRA study identifier; bioproject_accession, BioProject accession number; biosample_accession, BioSample accession number; library_source, source material type; library_selection, library selection method; platform, sequencing platform; filename, raw sequence file name; organism, organism name; host, host organism; isolation_source, biological source of the tissue; collection_date, tissue collection date; geo_loc_name, geographic location of tissue collection; lat_lon, geographic coordinates; samp_mat_process, sample material processing description. Full-length bacterial 16S rRNA genes were amplified using primers 8F and 1492R and sequenced using Oxford Nanopore Technology with the ligation sequencing amplicons kit (SQK-NBD114.96). The dataset corresponds to full-length 16S rRNA sequencing of the gastric microbiome from human stomach tissues. Host organism: Homo sapiens.*
